# Genomic identification and expression profiling of *WRKY* genes in alfalfa (*Medicago sativa*) elucidate their responsiveness to seed vigor

**DOI:** 10.1186/s12870-023-04597-x

**Published:** 2023-11-16

**Authors:** Shoujiang Sun, Wen Ma, Peisheng Mao

**Affiliations:** College of Grassland Science and Technology, China Agricultural University, Beijing, 100193 China

**Keywords:** Alfalfa, WRKY gene family, Seed vigor, Seed aging, Expression profiling

## Abstract

**Background:**

Seed aging is a critical factor contributing to vigor loss, leading to delayed forage seed germination and seedling growth. Numerous studies have revealed the regulatory role of WRKY transcription factors in seed development, germination, and seed vigor. However, a comprehensive genome-wide analysis of *WRKY* genes in Zhongmu No.1 alfalfa has not yet been conducted.

**Results:**

In this study, a total of 91 *MsWRKY* genes were identified from the genome of alfalfa. Phylogenetic analysis revealed that these *MsWRKY* genes could be categorized into seven distinct subgroups. Furthermore, 88 *MsWRKY* genes were unevenly mapped on eight chromosomes in alfalfa. Gene duplication analysis revealed segmental duplication as the principal driving force for the expansion of this gene family during the course of evolution. Expression analysis of the 91 *MsWRKY* genes across various tissues and during seed germination exhibited differential expression patterns. Subsequent RT-qPCR analysis highlighted significant induction of nine selected *MsWRKY* genes in response to seed aging treatment, suggesting their potential roles in regulating seed vigor.

**Conclusion:**

This study investigated *WRKY* genes in alfalfa and identified nine candidate WRKY transcription factors involved in the regulation of seed vigor. While this finding provides valuable insights into understanding the molecular mechanisms underlying vigor loss and developing new strategies to enhance alfalfa seed germinability, further research is required to comprehensively elucidate the precise pathways through which the *MsWRKY* genes modulate seed vigor.

**Supplementary Information:**

The online version contains supplementary material available at 10.1186/s12870-023-04597-x.

## Introduction

Transcription factors (TFs) are essential regulatory molecules that modulate gene expression by binding specifically to DNA sequences within cis-regulatory elements, either activating or repressing target genes. TFs also play a critical role in vital processes like hormone signaling responses, plant growth and development, and defenses against various stresses, such as biotic and abiotic stresses [1, 2]. Numerous TFs have been identified, each with diverse functionalities, including well-studied families like MADS, GRAS, and MYB [3–5]. Recent scientific attention has been directed towards investigating the role of WRKY TFs in plant growth and development, highlighting their fundamental importance in these biological processes. The *WRKY* gene family has been categorized into distinct subfamilies based on phylogenetic relationships, providing valuable insights into their evolutionary heritage [6].

WRKY proteins may contain multiple domains, whereas others contain only highly conserved WRKY DNA-binding domains, which are a common feature of WRKY TFs [7, 8]. The most prominent features of the WRKY family include an N-terminus that harbors a WRKYGQK heptapeptide sequence domain and a C-terminus that is a zinc finger structure composed of C-X4–5-C-X22–23-HXH (C_2_H_2_) or C-X7-C-X23-HXC (C2HC) [9]. The WRKY protein generally has one or two WRKY domains and, based on this number, can be classified into three groups (I–III). Group I proteins contain two WRKY domains and a C_2_H_2_ motif; Group II and III proteins only have a single WRKY domain and have a distinct zinc finger motif. The former has the C_2_H_2_ motif and is further divided into five subgroups (IIa-e).

WRKY TFs play diverse roles in plant growth and development, including plant flowering time regulation, seed development, dormancy, germination, and size regulation [10]. In recent years, more and more studies have revealed the key role of the *WRKY* gene family in the regulation of seed germination. These genes regulate multiple physiological and cellular processes, including energy metabolism, transcriptional regulation, protein synthesis, activation of ABA, and gibberellin (GA) signaling, ultimately contributing to successful seed germination [10]. For instance, certain members of the *AtWRKY* family, such as *AtWRKY2*, *AtWRKY40*, and *AtWRKY63*, play a crucial role in promoting seed germination by dampening ABA signaling. This effect is achieved by downregulating ABA-responsive genes, such as *AtEM1*, *AtEM6*, *AtABI3*, *AtABI4*, and *AtABI5* [11–13]. In contrast, various other Arabidopsis WRKY transcription factors, including *AtWRKY6*, *AtWRKY18*, *AtWRKY43*, and *AtWRKY60*, function as inhibitors of seed germination by promoting the expression of ABA-responsive genes [12, 14, 15].

Abiotic stresses, such as drought, saline–alkali stress, and seed aging significantly impact seed germination. In recent scientific investigations, there is growing evidence indicating that *WRKY* genes play multifaceted roles in enhancing plant tolerance to seed aging. Specifically, the MAPK signaling pathway, involving key components such as *WRKY6* (WRKY domain-containing protein 6), *MPK3* (mitogen-activated protein kinase 3), and *PLY4* (putative plastid lipoyltransferase 4), has been found to exhibit differential expression patterns during seed aging, suggesting their involvement in the response to seed aging [16]. Despite the important findings, the thorough identification and characterization of WRKY TFs governing seed vigor in alfalfa (*Medicago sativa*) are still ongoing areas of investigation. The exact roles of these TFs in seed germination, storage, and vigor regulation remain incompletely understood, leaving important gaps in our current understanding of these essential processes. Further research in this direction is warranted to unravel the intricate mechanisms underlying the contribution of WRKY TFs to seed-related aspects in alfalfa and their potential applications in enhancing seed quality and stress resilience.

Alfalfa is a vital forage species with extensive cultivation areas that encompass approximately 32 million hectares across North America, Europe, and Oceania [17]. In China, alfalfa is extensively cultivated across approximately four million hectares in the arid regions of the north [18]. However, the vigor of seed tends to decline during storage, leading to significant reductions in seed vitality and delays in seedling emergence in the field, ultimately resulting in decreased hay yield and economic losses. Therefore, the production of high-quality alfalfa seeds assumes a pivotal role in optimizing efficient hay production. In 2020, the release of the Zhongmu No.1 alfalfa genome provided a valuable resource for investigating the entire *WRKY* gene family in alfalfa [19]. Given the vital roles of WRKY TFs in plants and the lack of prior information on the alfalfa WRKY gene family, this study aims to identify *WRKY* genes that potentially regulate seed vigor. The research encompasses diverse analyses, such as chromosome mapping, phylogenetic assessments, conserved domain examination, prediction of cis-regulatory elements and subcellular localization, as well as tissue-specific expression profiling. Furthermore, *MsWRKY* genes, which demonstrate significant response to seed aging, will undergo RT-qPCR expression analysis. The comprehensive investigation on the *WRKY* gene family of alfalfa will provide an important basis for improving seed vigor and stress resistance.

## Results

### Identification and multiple sequence analysis of *WRKY* genes in alfalfa

**Fig. 1:**
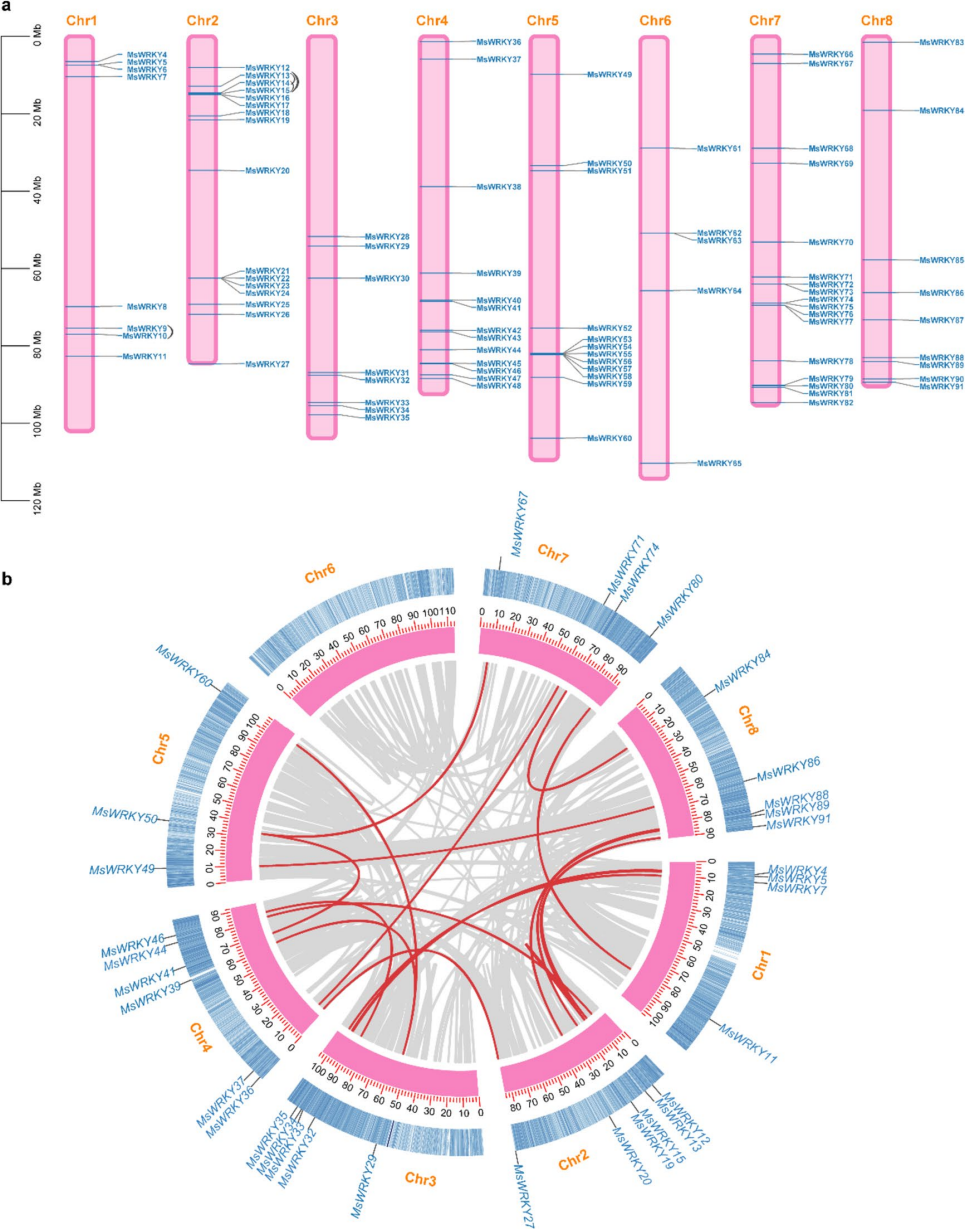
Chromosomal distribution and duplication events of MsWRKY proteins. **a** The tandem duplicated genes are marked by black arc trajectory. **b** The segmentally duplicated genes are connected by red lines, referring to the 33 genes highlighted in blue

To investigate the presence and distribution of *MsWRKY* genes in alfalfa, we conducted a thorough screening of the Zhongmu No.1 alfalfa genome using a combination of the hidden Markov model (HMM) profile and BLAST searches. We employed 358 WRKY protein sequences from *Arabidopsis thaliana*, *Oryza sativa*, and *Medicago truncatula* as queries. Subsequently, a total of 91 putative MsWRKY proteins were identified and confirmed using the Pfam database (http://pfam.xfam.org) based on the presence of the conserved WRKY domain (PF03106). The assigned nomenclature for these proteins ranged from MsWRKY01 to MsWRKY91 (Table S1), facilitating systematic reference. Genomic locations of the 91 identified *MsWRKY* genes were subsequently mapped onto the eight chromosomes of alfalfa (Chr1–8), as illustrated in Fig. 1a. The distribution of the *MsWRKY* genes exhibited unevenness across the eight chromosomes. Chr6 contained six *MsWRKY* genes (6.6% of the total), namely, *MsWRKY61*, *MsWRKY62*, *MsWRKY63*, *MsWRKY64*, and *MsWRKY65*. Conversely, the majority of *WRKY* genes were localized on Chr7 (*n* = 17, 18.7%), followed by Chr1 (*n* = 8, 8.8%), Chr2 (*n* = 16, 17.6%), Chr3 (*n* = 8, 8.8%), Chr4 (*n* = 13, 14.3%), Chr5 (*n* = 12, 13.2%), and Chr8 (*n* = 9, 9.9%). In this study, we found that four pairs of *WRKY* genes were evidence of tandem duplication events, i.e., *MsWRKY9* vs. *MsWRKY10*, *MsWRKY13* vs. *MsWRKY14*, *MsWRKY13* vs. *MsWRKY15*, and *MsWRKY14* vs. *MsWRKY15*, which are located on chromosomes 1 and 2 (Fig. 1a and Table S2). In addition to tandem duplication events, 18 segmental duplication events were also identified for 33 *MsWRKY* genes, which are located on chromosomes 1, 2, 3, 4, 5, 7, and 8 (Fig. 1b and Table S2). Through multiple comparisons, we found that all duplication genes were full-length duplications. At present, the definition of promoter region is not clear, and most genomes do not have promoter annotation. Therefore, we conducted multiple comparisons of the 2000 bp sequences upstream of the start sites of these duplication genes and found that these gene promoter regions were full-length duplications. These duplication events may indicate potential gene expansion, and functional diversification within the *MsWRKY* gene family was dominated by segmental duplication in alfalfa.

**Fig. 2:**
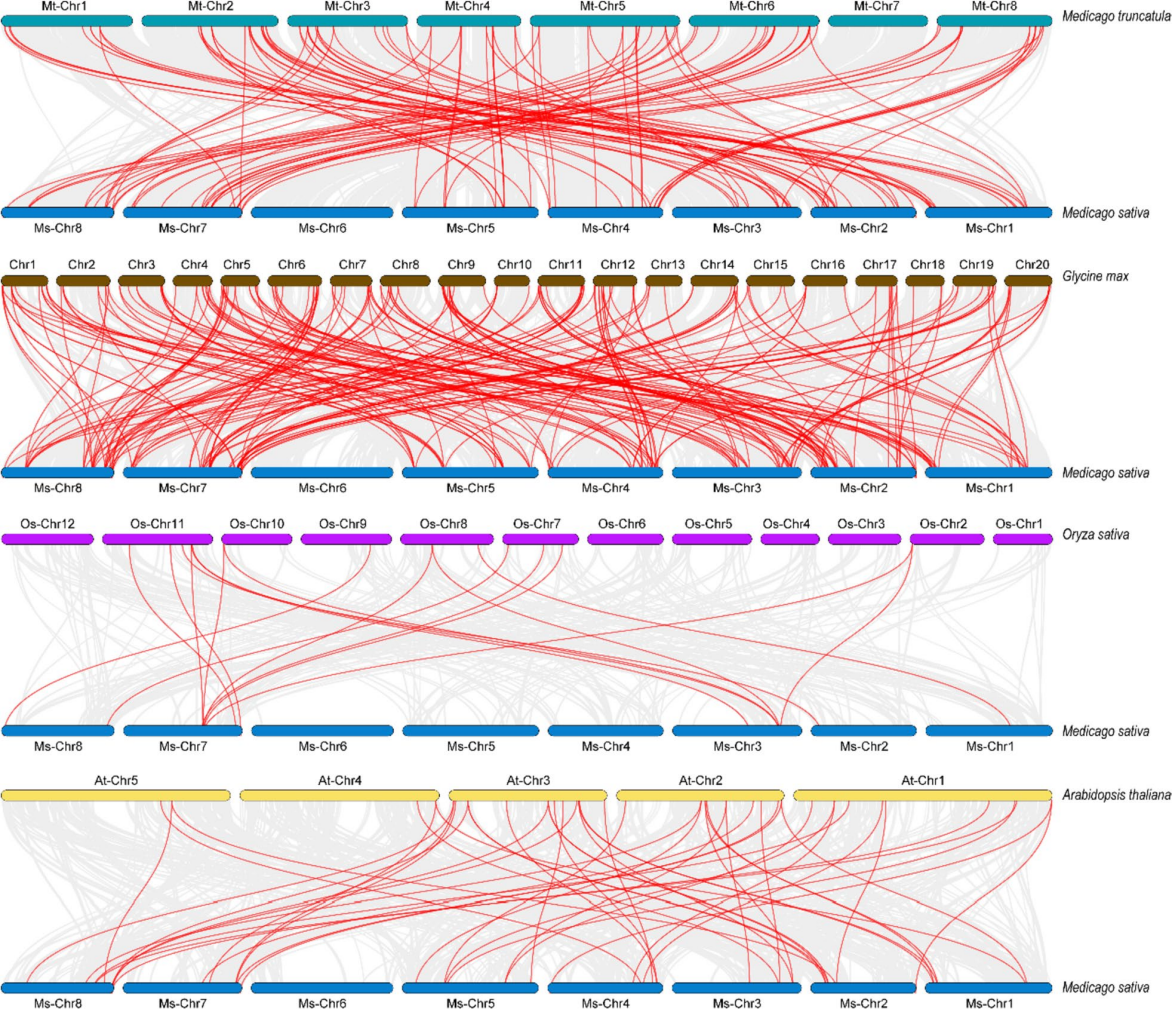
Syntenic analysis of *MsWRKY* genes in *M. sativa* in comparison with those in four plant species (*M. truncatula*, *G. max*, *O. sativa*, and *A. thaliana*)

To further investigate the evolutionary relationships within the WRKY gene family, we performed a collinearity analysis encompassing *MsWRKY* genes from four plant species: a monocotyledonous plant, *Oryza sativa*, and three dicotyledonous plants, *Arabidopsis thaliana*, *Glycine max*, and *Medicago truncatula*, as depicted in Fig. 2. The collinearity analysis allowed us to ascertain the potential orthologous gene pairs among these species, shedding light on the conservation and divergence of *WRKY* genes across different plants. The results of our analysis revealed the presence of homologous gene pairs between *M. truncatula* and *M. sativa* (107 pairs), *Glycine max* and *M. sativa* (212 pairs), *O. sativa* and *M*. *sativa* (17 pairs), and *A. thaliana* and *M. sativa* (47 pairs). *MsWRKY* genes displayed various numbers of syntenic lines with four species. A total of 65 *MsWRKY* genes showed a syntenic relationship with those in *G. max*, and 70 corresponding orthologs were identified between *M. sativa* and *M. truncatula*. Meanwhile, only 10 and 33 *MsWRKY* genes showed syntenic relationships with those in *O. sativa* and *A. thaliana*, respectively (Fig. 2 and Table S3).

The results of sequence analyses revealed considerable variation in the size of the MsWRKY proteins, with lengths ranging from 68 amino acids (aa) in MsWRKY64 to 976 aa in MsWRKY55. The molecular weight of these proteins exhibited a broad range, spanning from 7.98 kilodaltons (kD) in MsWRKY9 to 112.05 kD in MsWRKY55. Additionally, the isoelectric point (pI) of the MsWRKY proteins displayed diversity, varying from 4.3 in MsWRKY33 to 11.13 in MsWRKY35. Regarding subcellular localization, the prediction results indicated that the majority of MsWRKY proteins (76 out of 91) were located within the nucleus, which is consistent with their role as transcription factors. However, a subset of MsWRKY proteins displayed diverse subcellular localization patterns, with two proteins in the mitochondria, four in the chloroplast, four in the cytoplasm, and five in the plasma membrane (Table S4). This diversity in subcellular localization implies that MsWRKY proteins may participate in various microcellular environments, potentially playing distinct roles beyond transcriptional regulation.

### Phylogenetic analysis, gene structures, and motif composition of *MsWRKY* genes

**Fig. 3:**
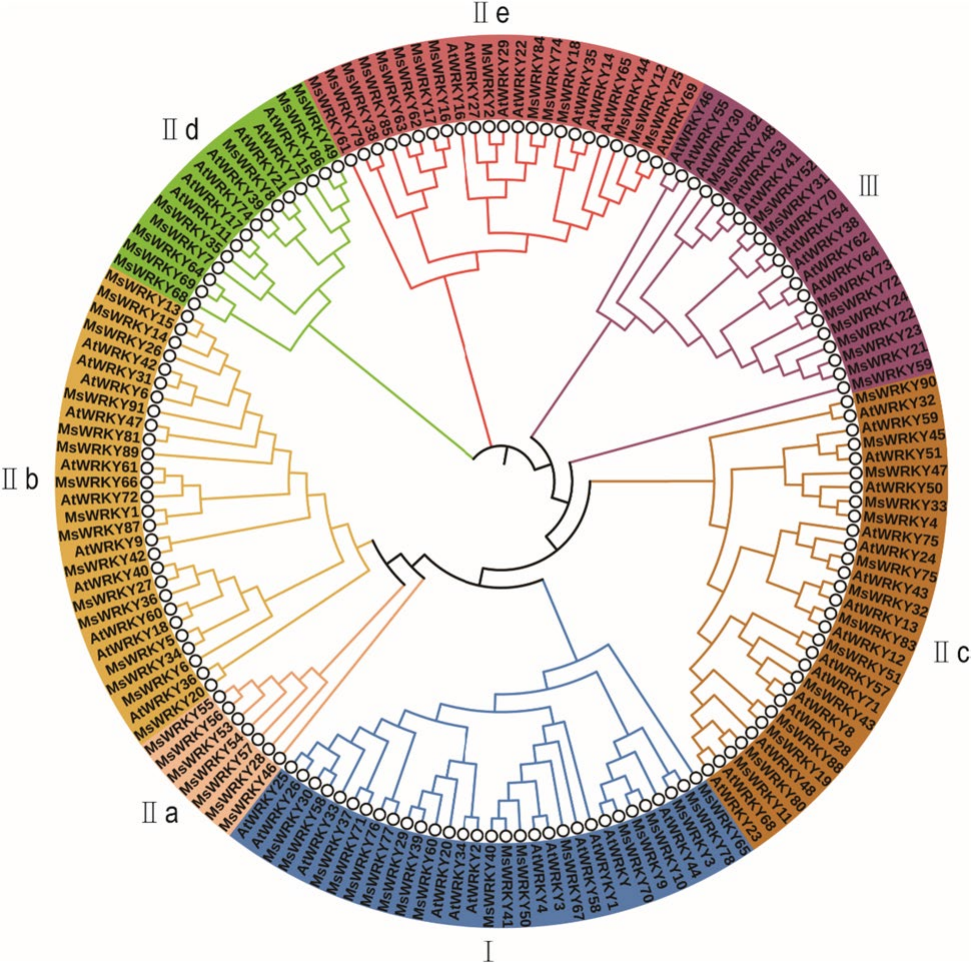
Phylogenetic tree of WRKY members in alfalfa and Arabidopsis. MsWRKY and AtWRKY protein sequences were aligned with ClustalW, and a phylogenetic tree was constructed with MEGA7.0 using the neighbor-joining method and 1000 bootstrap replicates. The members were divided into Groups I, II, and III, and Group II was further divided into Subgroups IIa, IIb, IIc, IId, and IIe

To comprehensively unravel the evolutionary process of the *WRKY* genes in alfalfa, a comparative analysis was performed between the 91 identified *MsWRKY* genes and 75 *WRKY* genes from Arabidopsis (https://www.arabidopsis.org/index.jsp). According to previous studies on WRKY genes, their phylogenetic relationships, and WRKY domain characteristics of *A*. *thaliana*, the WRKY genes of alfalfa and Arabidopsis could be divided into three groups, namely, I, II, and III (Fig. 3). This classification is consistent with the known organization of *WRKY* genes in *A. thaliana* [20] and *M. truncatula* [21].

**Fig. 4:**
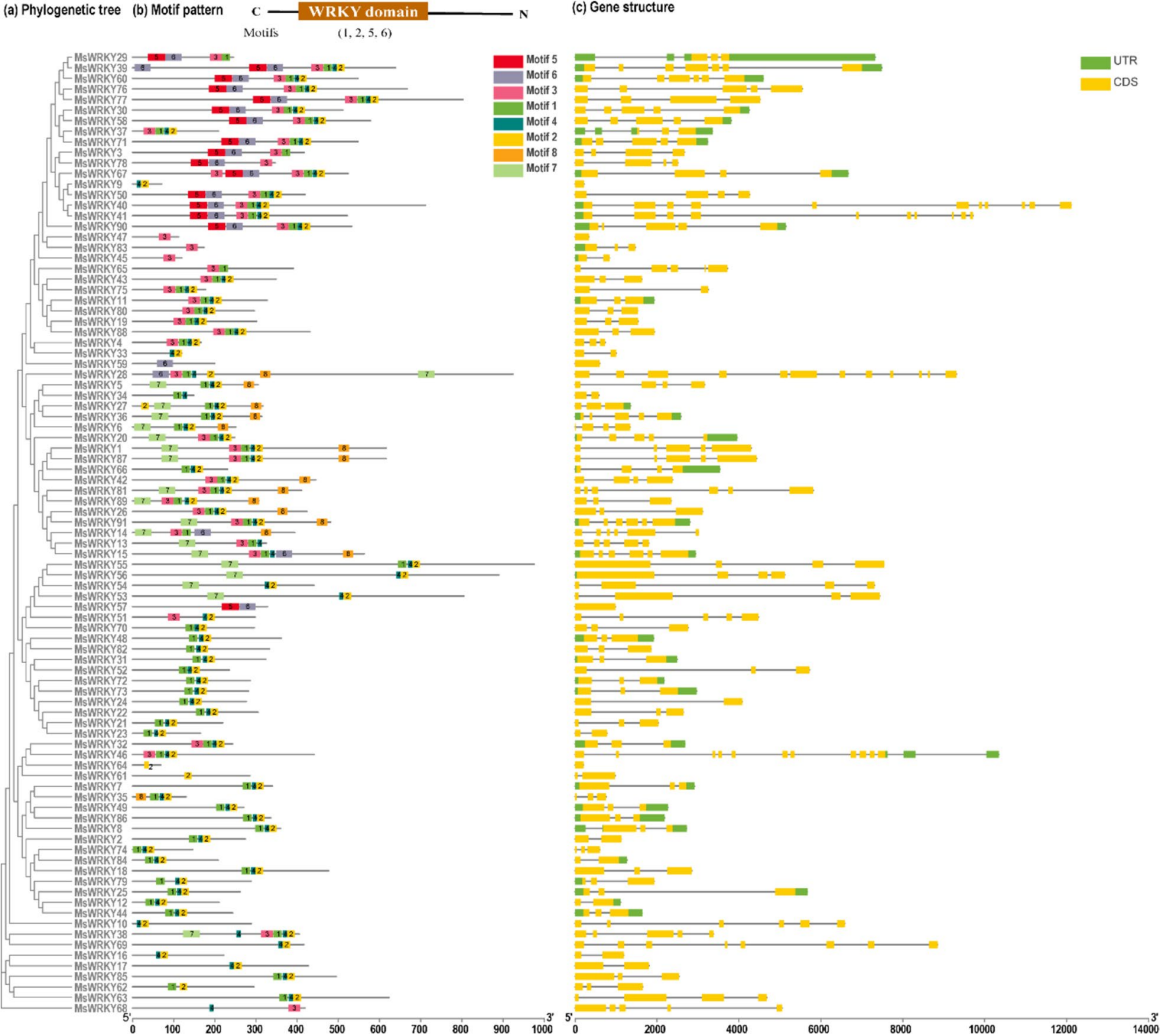
Phylogenetic tree, conserved motif, and gene structure of MsWRKY family members. **a** Phylogenetic tree of *MsWRKY* genes was constructed using MEGA-X and the neighbor-joining (NJ) method. **b** Conserved motif of MsWRKY proteins was analyzed on the MEME tool, and the results were visualized in TBtools. The motifs, labeled as 1–8 and represented by different colored boxes, demonstrate the conserved patterns in the protein sequences. The scale at the bottom allows for estimation of protein lengths. **c** The gene structure of *MsWRKY* genes was determined, with the coding regions for the MsWRKY domain highlighted in yellow. Regions labeled as CDS without the region coding for the MsWRKY domain, indicated in green

To gain deeper insights into the diversity and conserved motifs of MsWRKY proteins, a comprehensive analysis of conserved motifs was conducted using the MEME tool. As a result, a total of eight distinct conserved motifs, designated as Motif 1 to Motif 8, were identified and annotated in 91 MsWRKY proteins, with motif lengths ranging from 11 to 43 amino acids (Fig. 4a and Table S5). As shown in Fig. 4b, within the same group, MsWRKY members usually have a similar motif composition that ranged from one to eight. Except for MsWRKY47/83/45/59/68, almost all proteins contain Motifs 1and 2 that define WRKY domains and zinc finger structures, respectively. Class III members typically exhibiting a combination of Motif 1, Motif 2, and Motif 4. Interestingly, Class I family members displayed relatively shorter WRKY protein sequences and showcased a more pronounced conserved motif composition when compared to other subfamilies. These results indicate that proteins with the same or similar structures may be functionally or evolutionarily similar, and these results demonstrate the reliability of the classification (Fig. 4b).

In the investigation of conserved domains, it became apparent that the exon–intron architecture of *WRKY* genes played a significant role in their functional diversity. The analysis of intron–exon structures revealed that the vast majority of *MsWRKY* genes exhibited a paucity of introns, with many genes displaying either very few or no introns at all. Notably, a subset of *MsWRKY* genes, including *MsWRKY66*, *MsWRKY2*, *MsWRKY42*, and *MsWRKY1*, contained four introns, while *MsWRKY10* possessed two introns (Fig. 4c). This variation in the intron–exon structure highlights the potential contribution of these structural features to the functional divergence and specificity of certain *MsWRKY* genes.

### Cis-regulatory element analysis of *MsWRKY* gene promoters

To gain deeper insights into the regulatory mechanisms governing the alfalfa WRKY gene family, we extracted the 2000 bp promoter regions upstream of all *MsWRKY* genes from the alfalfa genome assembly to identify cis-acting elements using the online tool PlantCARE. Thus, various cis-acting regulatory elements were found in all MsWRKY gene promoter regions. We identified fifteen distinct types of cis-regulatory elements. These cis-regulatory elements encompassed a range of functional categories, including methyl jasmonate (MeJA), multiple light-responsive elements, ABA-responsive element (ABRE), salicylic acid-responsive element (TCA-element), gibberellin-responsive element (GARE-motif), LTR (low-temperature responsive element), MBS (an MYB transcription factor binding site involved in drought inducibility), and TC-rich repeats (defense-responsive and stress-responsive elements). However, we also identified seed-specific regulation, circadian control, and GC-motif (anoxic specific inducibility element). The analyses of cis-regulatory elements revealed that light responsiveness was the most prevalent type, followed by salicylic acid responsiveness, abscisic acid responsiveness, drought inducibility, and anaerobic induction. The promoter-related and binding sites elements found included TATA-box, CAAT-box, A-box, HD-Zip, and W-box (a classic WRKY DNA-binding motif). These observations suggest that these cis-regulatory elements likely play vital roles in mediating the transcriptional regulation of the *MsWRKY* genes in response to various environmental cues and stress conditions. Furthermore, TATA and CAAT-box cis-regulatory elements were identified in promoter regions of all *MsWRKY* genes, indicating their widespread involvement in transcriptional regulation. These ubiquitous regulatory elements suggest that the *MsWRKY* genes are extensively engaged in controlling various biological processes and stress responses through transcriptional regulation (Table S6).

### Secondary structure analysis of *MsWRKY* proteins

The investigation of protein secondary structure is of paramount importance for gaining insights into protein function. In this study, the results revealed that among the various secondary structure elements, the random coil constituted the largest proportion, ranging from 19.12% to 78.10% across the MsWRKY proteins. Following the random coil, the α-helix was the next most-prominent secondary structure, ranging from 6.93% to 56.18%. The extended strand, another key secondary structure element, accounted for 6.08% to 26.44% of the protein sequences, while the β-turn was present at lower proportions, ranging from 1.53% to 11.76% (Table S7).

### Analysis of the expression patterns of *MsWRKY* genes in various alfalfa tissues

**Fig. 5:**
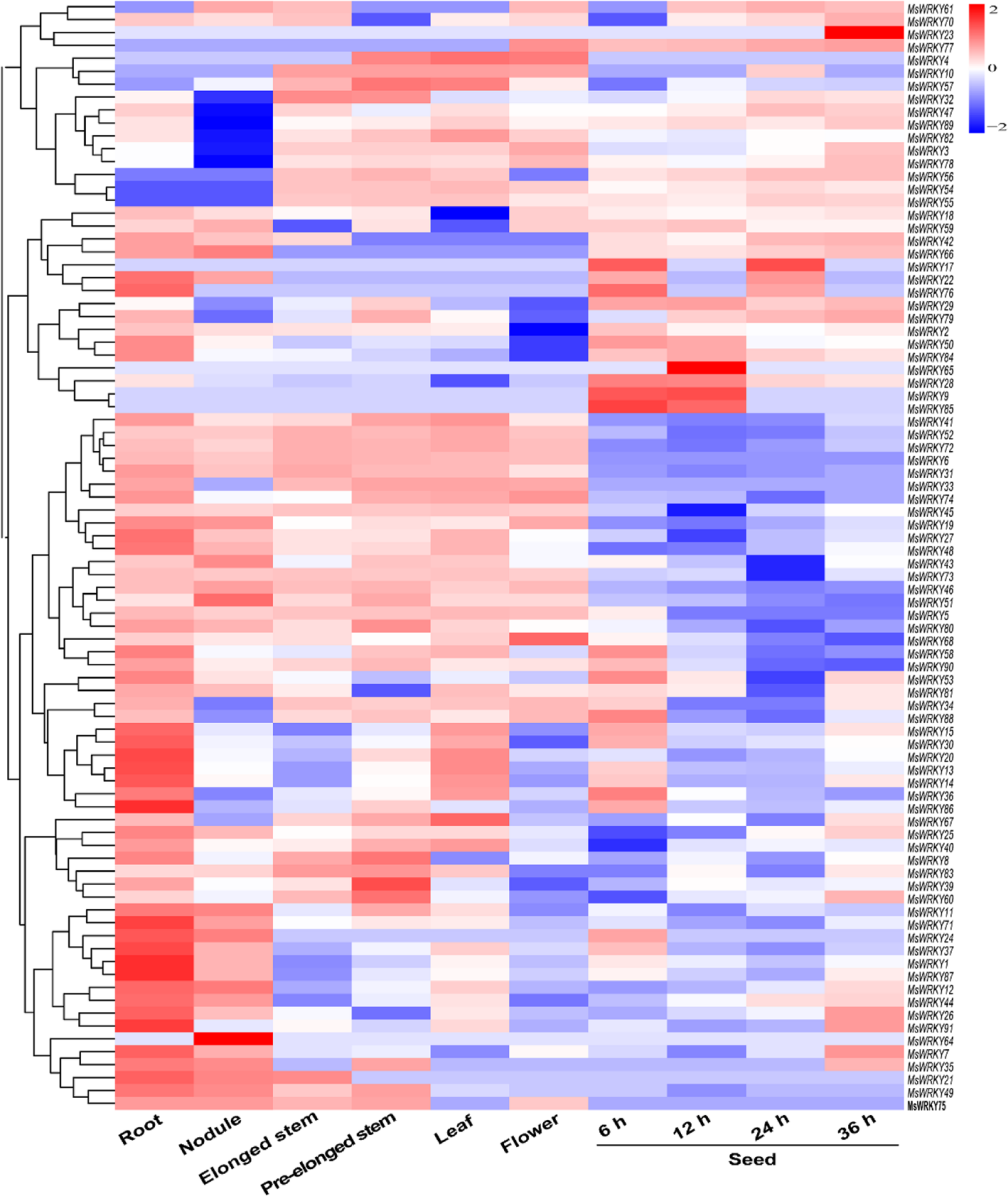
Tissue-specific expression analysis of *MsWRKY* genes

Temporal and spatial expression analysis of genes is pivotal for comprehending the specific functions of *MsWRKY* genes in various tissues of alfalfa. The RNA-Seq data, which were downloaded from Noble Research Institute database (https://www.alfalfatoolbox.org), were used to evaluate transcript abundance profiles of *MsWRKY* encoding genes across seven tissues, namely, leaves, flowers, pre-elongated stems, elongated stems, roots, nodules, and seed imbibition at different times (6, 12, 24, and 36 h) (Table S8). To visually represent these expression patterns, a heatmap of the expression profile was constructed using TBtools software (Fig. 5). The analysis of gene expression patterns revealed distinct transcript abundance profiles of *MsWRKY* genes in different tissues. Notably, *MsWRKY71*, *MsWRKY24*, *MsWRKY37*, *MsWRKY1*, *MsWRKY87*, *MsWRKY12*, and *MsWRKY86* exhibited the highest transcript accumulation levels in the root tissue. Conversely, *MsWRKY64* was found to be specifically expressed in nodules. Among other genes, *MsWRKY67*, *MsWRKY8*, *MsWRKY39*, and *MsWRKY60* showed the highest transcript accumulation in elongated stems, while *MsWRKY4*, *MsWRKY57*, and *MsWRKY40* displayed pronounced expression in leaves. Additionally, during seed germination, *MsWRKY23*, *MsWRKY17*, *MsWRKY76*, *MsWRKY65*, *MsWRKY28*, *MsWRKY9*, and *MsWRKY85* exhibited prominent transcript accumulation.

**Fig. 6:**
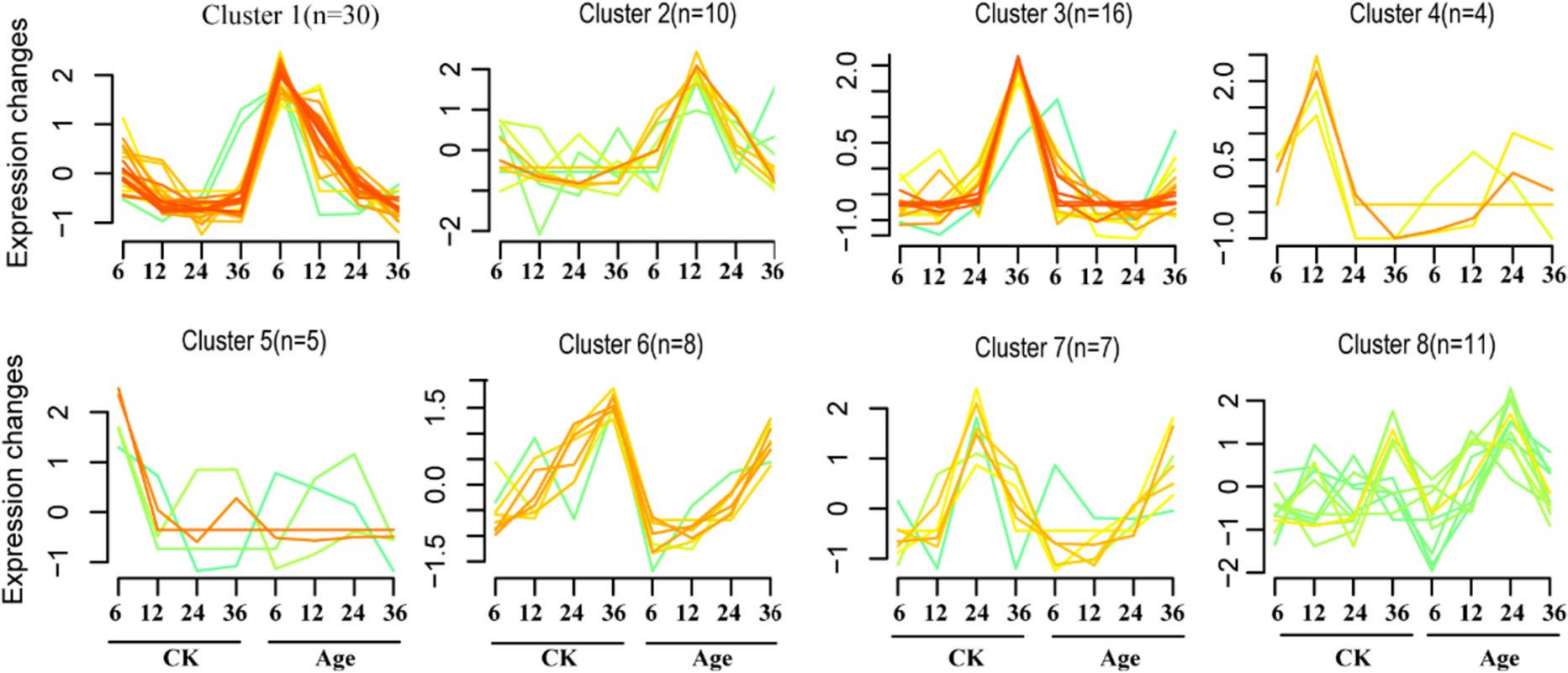
Expression profiling of *MsWRKY* genes during seed imbibition for 6, 12, 24, and 36 h in seeds aged for 0 days (CK) and aged for 10 days (Age). Fuzzy c-means clustering identified eight distinct temporal patterns of gene expression. The x-axis represents imbibition time, while the y-axis represents log_2_-transformed, normalized intensity ratios in each stage

To identify key WRKY transcription factors that regulate seed germination and respond to seed aging, a comprehensive clustering analysis was conducted using the R package Mfuzz (http://mfuzz.sysbiolab.eu) (Fig. 6). In this analysis, the expression patterns of all 91 *MsWRKY* genes during seed (CK and aged) germination were categorized into six distinct clusters. Cluster 1 exhibited the largest number of *MsWRKY* genes (30 in total), and their expression levels decreased with the progression of seed germination in the control (CK) but showed an opposite trend with increased expression during aged seed germination (Age). This dynamic expression pattern suggests that the genes in Cluster 1 might be involved in the response to seed aging, potentially playing a role in mitigating the adverse effects of aging on seed vigor. Cluster 2 comprised 10 *MsWRKY* genes, characterized by a significant increase in their expression levels during aged seed imbibition for 12 h. This observation implies that these genes may possess a positive regulatory function in the aging process of seeds. Conversely, Cluster 4 encompassed four *MsWRKY* genes exhibiting a substantial decrease in expression levels during aged seed germination. This decline suggests that these genes may be suppressed or inhibited by seed aging.

### RNA-Seq data validation

**Fig. 7:**
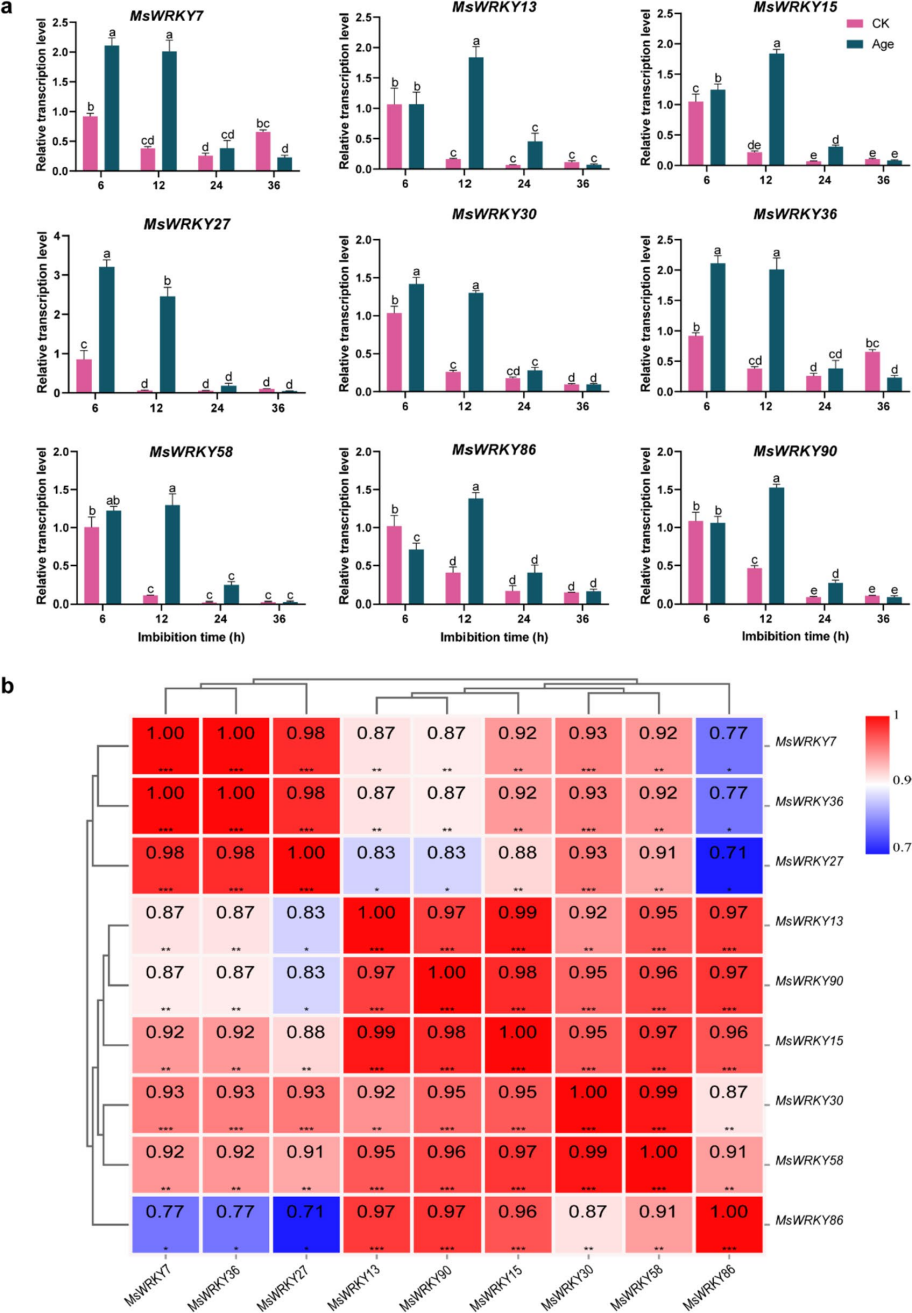
Gene expression analysis of nine selected *MsWRKY* genes using RT-qPCR assay. **a** Gene expression levels of nine *MsWRKY* genes during seed (CK and aged) imbibition for 6, 12, 24, and 36 h after aging treatment, as determined with RT-qPCR assay. The data represent the means of three biological replicates ± SEM. Different lowercase letters indicate significant differences. **b** Correlation analysis of nine *MsWRKY* genes based on the relative expression of genes. The numbers in the box represent the Pearson’s correlation coefficient, and *, **, and *** indicate correlations at the 0.05, 0.01, and 0.001 levels, respectively. Red indicates a positive correlation, and blue indicates a negative correlation

Based on RNA-Seq data, nine *MsWRKY* genes (*MsWRKY7*, 13, 15, 27, 30, 36, 58, 86, and 90), which exhibited significant induction in response to seed aging treatment, were selected to validate the credibility of the RNA-Seq data (Fig. 7a). The results demonstrated that all nine *MsWRKY* genes exhibited upregulation during seed germination, consistent with the RNA-Seq findings. Specifically, *MsWRKY7*, *MsWRKY15*, *MsWRKY27*, *MsWRKY30*, and *MsWRKY36* showed significant upregulation at 6 and 12 h of aged seed imbibition (*p* < 0.05). Furthermore, the expression of *MsWRKY7* and *MsWRKY36* significantly decreased at 36 h of aged seed imbibition (*p* < 0.05), indicating their potential role as negative regulators. In summary, most of the *MsWRKY* genes showed consistent expression patterns in both the RT-qPCR experiment and the RNA-Seq analysis. Moreover, a correlation analysis was conducted on the expression levels of the nine *MsWRKY* genes, revealing noteworthy positive correlations among the majority of the genes, thereby providing additional support for the reliability and consistency of the expression data (Fig. 7b).

## Discussion

WRKY TFs play diverse roles in plant growth and development, including plant flowering time regulation, seed development, dormancy, germination, and size regulation [10]. WRKY TFs have been extensively studied in many plants, such as *Sorghum bicolor* [22], *A. thaliana* [20], *Hordeum vulgare* [23], *M. truncatula* [21], and *Solanum lycopersicum* [24]. In this study, a total of 91 *MsWRKY* genes were successfully identified from the Zhongmu No.1 alfalfa genome. The diverse molecular weights, amino acid sequence lengths, and theoretical isoelectric points observed among the 91 *MsWRKY* genes revealed the complexity of the alfalfa genome and highlighted the functional divergence of *MsWRKY* genes in response to various stresses. Interestingly, the number of WRKY genes in alfalfa (91) surpassed those found in *A. thaliana* (75), *S. lycopersicum* (81), *Phaseolus vulgaris* (90), *Lotus japonicus* (83), and *Cicer arietinum* (61), underscoring the genomic expansion and complexity in the alfalfa WRKY gene family. Furthermore, the distribution of *MsWRKY* genes on the eight chromosomes provided insights into gene duplication events. About 5.5% (5/91) of *MsWRKY* genes were found to evolve from tandem gene duplication (Fig. 1a), and 36% (33/91) were found to evolve from segmental gene duplication (Fig. 1b), suggesting that segmental gene duplication probably played a pivotal role in WRKY gene expansion in alfalfa genome. The occurrence of gene replication events significantly contributed to the evolutionary trajectory of the species, offering potential raw materials for generating novel gene functions and expanding gene families.

The topology of our phylogenetic tree obtained from WRKYs in two species (Arabidopsis and alfalfa) is largely consistent with that derived from two species (Arabidopsis and rice) [25]. In terms of evolutionary relationships, phylogenetic analysis effectively classified the *MsWRKY* genes into seven subgroups, specifically named as I, II a, II b, II c, II d, II e, and III. This classification showed a closer similarity to the classifications found in *A. thaliana* [26], *O. sativa* [27], and *G. max* [28]. Such comprehensive phylogenetic insights shed light on the evolutionary dynamics of the *MsWRKY* gene family and its relationship to other well-studied plant species. Protein secondary structure is one of the key factors for predicting protein function. It directly influences the stability of the protein [29]. The findings of this investigation indicate that *MsWRKY* proteins predominantly feature random coil elements, with proportions ranging from 19.12% to 78.10%. Following the random coil, α-helix structures are also prominent, with percentages varying from 6.93% to 56.18%. Extended strand structures constitute a significant portion, accounting for 6.08% to 26.44% of the protein sequences, while β-turn structures are present at lower levels, ranging from 1.53% to 11.76% (Table S7). The study highlights the significance of analyzing protein secondary structure, as it provides valuable insights into protein functionality.

WRKY proteins are typically found in the cell nucleus and belong to a class of nuclear-localized transcription factors that regulate gene expression in plants [30]. They carry out their functions, such as controlling plant growth and response mechanisms, within the cell nucleus [31]. However, research has shown that, in some cases, WRKY proteins can also translocate from the cell nucleus to the cytoplasm, which may involve the regulation and interactions of their subcellular localization, especially in plant responses to stress conditions [12]. In this study, the silico subcellular localization prediction results indicated that the majority of *MsWRKY* proteins were located within the nucleus, which is consistent with their role as transcription factors. However, a subset of *MsWRKY* proteins displayed diverse subcellular localization patterns, with two proteins in the mitochondria, four in the chloroplast, four in the cytoplasm, and five in the plasma membrane (Table S4). These results suggest that these transcription factors may have multiple stress response mechanisms. Rushton et al. [30] also found that during seed germination and post-germination growth, ABA receptor (ABAR) and *AtWRKY40*, *AtWRKY18*, and *AtWRKY60* regulate ABA responses by a derepression mechanism that removes the WRKY repressor proteins from the nucleus. Binding of ABA by ABAR recruits AtWRKY40, AtWRKY18, and AtWRKY60 from the nucleus to the cytoplasm [8]. Some WRKY proteins lack nuclear localization signals and may require the assistance of nuclear localization proteins to enter the cell nucleus in order to carry out their gene expression regulation functions [30]. The specific WRKY proteins that require these auxiliary factors and the types of auxiliary factors they depend on may vary among different WRKY proteins. These factors are often influenced by the structure and function of the proteins as well as the cellular environment. Therefore, specific circumstances may vary, and further research is needed to determine which WRKY proteins require these auxiliary factors to enter the cell nucleus.

The diverse regulatory functions of TFs are governed by various cis-acting sites, leading to intricate gene regulatory networks [32]. WRKY proteins, as evidenced by multiple studies, exhibit functional diversity in the regulation of plant growth and stress responses. Many researches have uncovered hormone-responsive and stress-responsive elements in the promoter regions of *WRKY* genes in soybean and rice [6, 10, 20, 33]. In this study, an abundance of cis-regulatory elements related to hormones and stress responses were identified in the promoter regions of the *MsWRKY* genes. Notably, elements associated with hormone responses, such as gibberellin responsiveness, abscisic acid responsiveness, salicylic acid responsiveness, MeJA, auxin responsiveness, and GARE-motif, were frequently detected. Additionally, seed-specific regulatory elements were discovered, specifically occurring in eight *MsWRKY* genes belonging to the subfamily, which were significantly induced by seed aging treatment. This observation suggests that these specific genes may have essential roles in regulating seed vigor and contribute to maintaining seed quality under unfavorable conditions.

The WRKY gene family is involved in the regulation of plant responses to various abiotic and biotic stresses [33]. Gene expression profiles play a crucial role in predicting the potential functions of genes [10]. The observation revealed that 11 *MsWRKY* genes exhibited consistent expression patterns across all six tissues, suggesting their potential involvement in fundamental biological processes shared by these tissues. These findings offer valuable insights into the tissue-specific roles of *MsWRKY* genes and contribute to the comprehensive understanding of their functions in alfalfa.

WRKY protein plays an important role in the regulation of seed germination, positively or negatively regulating seed dormancy and germination, likely as part of a precise regulatory network that appropriately mediates the initiation of life cycles in response to changes in internal and external conditions [10]. In this investigation, we identified several *MsWRKY* genes, including *MsWRKY7*, *MsWRKY15*, *MsWRKY27*, *MsWRKY30*, *MsWRKY36*, *MsWRKY7* and *MsWRKY36*, that were significantly induced by seed aging treatment. *MsWRKY7and MsWRKY27*, which may be negative regulators of seed germination, were inhibited in the early stage of seed germination. This observation suggests that these genes might function as potential regulators of seed vigor during the aging process. The differential expression of key genes, such as MAPK signal pathway components MPK3, *WRKY6*, and PLY4, during seed aging further supports the crucial roles of *WRKY* genes in the response to seed aging [16]. These findings contribute to our understanding of the regulatory mechanisms underlying seed aging tolerance, shedding light on the potential applications of *MsWRKY* genes in improving seed vigor in alfalfa.

## Conclusion

In summary, our investigation identified 91 distinct *MsWRKY* genes and successfully mapped them onto the eight chromosomes of alfalfa. Phylogenetic analysis revealed their classification into seven distinct subfamilies, and subcellular localization prediction indicated that the majority of WRKY proteins are located within the nucleus. Structure analysis further demonstrated the high conservation of *MsWRKY* genes, with most of them possessing minimal or no introns. Among the 91 *MsWRKY* genes, nine were found to be significantly induced in response to seed aging, implying their potential involvement in maintaining seed vigor. Interestingly, their responses to aging treatments varied during seed imbibition. Notably, *MsWRKY7*, *MsWRKY27*, *MsWRKY36*, and *MsWRKY86* exhibited particularly pronounced responses, especially under aging stress. This indicates that these genes likely play a pivotal role in maintaining redox homeostasis. Our findings offer comprehensive insights into the molecular basis of *WRKY* gene families in alfalfa. However, the precise regulatory mechanisms underlying seed vigor and governed by these WRKY genes remain unclear. Hence, future studies focused on elucidating the individual functions of *MsWRKY* genes are of great interest. For instance, we hypothesize that *MsWRKY7* may contribute to oxidative stress tolerance during alfalfa seed storage, *MsWRKY27* might be involved in abscisic acid signal transduction, and *MsWRKY36* could play a pivotal role in the DNA repair pathway during alfalfa seed germination. Nevertheless, this study provides valuable perspectives for unveiling the factors contributing to the decline in seed vigor and presents new strategies for improving alfalfa seed vigor.

## Materials and methods

### Genomic identification of *WRKY* genes in alfalfa

In order to comprehensively identify the *MsWRKY* gene family in alfalfa, the genomic information of the Zhongmu No.1 alfalfa variety was retrieved from the figshare website. https://figshare.com/articles/dataset/Medicago_sativa_genome_and_annotation_files. Accessed 17 February 2023. To enhance the precision of this identification process, the WRKY protein sequences of Arabidopsis were obtained from the PlanetTFDB database website. Accessed 10 March 2023. and used as queries to search for possible WRKY proteins within the alfalfa genome using Blast P with a stringent E-value cutoff of 1.0 × 10^−10^. Furthermore, the identified WRKY protein sequences in alfalfa were subjected to multiple sequence alignment using the HMM model of HMMER3.0. Accessed 18 March 2023 with an E-value of 0.001. Redundant sequences were removed from the alignment to ensure accuracy. The hidden Markov model (HMM) profile (PF03634) from the Pfam database (http://pfam.xfam.org, accessed 20 March 2023) [34] was utilized to verify the presence of the putative WRKY domain in the identified sequences. Nonredundant *MsWRKY* genes were designated as *MsWRKY1* to *MsWRKY91*, corresponding to their order of appearance in the genome file. The physical and chemical properties of the MsWRKY proteins were analyzed using the ProtParam Tool (https://web.expasy.org/protparam, accessed 28 March 2023). Additionally, the subcellular localization was predicted using the WoLF-PSORT tool (https://www.genscript.com/wolf-psort.html, accessed 3 April 2023). The secondary structure prediction of MsWRKY proteins was performed through the online tool SOPMA (https://npsa-prabi.ibcp.fr/cgi-bin/npsa_automat.pl?page=/NPSA/npsa_sopm.html, accessed 10 May 2023).

### Phylogenetic, gene structure, and conserved motif analyses of the *MsWRKY* genes

To explore the evolutionary relationships of *MsWRKY* genes in alfalfa, a multiple sequence alignment was performed using Clustal W. Accessed 10 April 2023. An unrooted phylogenetic tree was then constructed to compare the *WRKY* genes of alfalfa (91) and Arabidopsis (75). This was achieved using the online tool Interactive Tree Of Life (iTOL) v5 [35] and the neighbor-joining (NJ) method with 1000 bootstrap replicates. In addition to the NJ method, other phylogenetic tree construction methods were also employed to ensure robustness and reliability in the tree topology [36]. To classify the *MsWRKY* genes based on their evolutionary relationships with Arabidopsis *WRKY* genes, a thorough analysis of the phylogenetic tree was carried out. Furthermore, conserved motifs in the *MsWRKY* proteins were identified using the MEME tool. Accessed 18 April 2023 [37].

### Chromosome localization and gene duplication events

The physical locations in the alfalfa genome of *MsWRKY* genes were extracted from the Zhongmu No.1 alfalfa genome database. Subsequently, the chromosomal positions of the identified *WRKY genes* were determined from the alfalfa genomic annotation file. Visualization of the chromosomal distribution of *MsWRKY* genes was accomplished using the TBtools software [38]. To facilitate the identification and organization of the putative *WRKY* genes in alfalfa, they were systematically renamed according to their specific chromosomal locations. To assess gene duplication events within the *MsWRKY* gene set, multiple collinear scanning toolkits (MCScanX) were employed with an E-value of 10^–5^ [39]. Furthermore, to gain insights into the evolutionary conservation and relationships of *MsWRKY* genes with other plant species, synteny analysis was conducted between Zhongmu No.1 alfalfa and *Medicago truncatula*, *Glycine max*, *Oryza sativa*, and *Arabidopsis thaliana*. The exon–intron structures of the *MsWRKY* genes were visually represented by drawing comparisons between their coding sequences (CDS) and corresponding full-length sequences using the TBtools software [38].

### Cis-regulatory element analysis

This study aims to investigate the functional roles of *MsWRKY* genes in response to seed aging stress in plants by conducting a detailed analysis of the cis-regulatory elements. The 2000 bp upstream sequences from the translation start site of all *MsWRKY* genes were analyzed using the PlantCARE database. Accessed 10 May 2023 [40].

### Tissue-specific expression analysis of *MsWRKY* genes in alfalfa

The characterization of tissue-specific expression patterns of *MsWRKY* genes in alfalfa is pivotal in deciphering their functional roles within specific plant tissues. The RNA-Seq data, which have been published by Dong et al. [41], were downloaded from the Noble Research Institute database to analyze tissue-specific expression patterns of *MsWRKY* genes. Accessed 10 May 2023. Additionally, RNA-Seq data from previous experiments involving aged seed imbibition at time points of 6, 12, 24, and 36 h were utilized to augment genetic information concerning the response of *MsWRKY* genes to seed aging. These datasets were used to investigate the expression profiles of *MsWRKY* genes across various tissues, and the expression patterns of all genes were visualized with the TBtools software [38]. Furthermore, a correlation analysis was performed using the Corrplot Package in R.

### Plant materials and seed aging treatments

**Fig. 8:**
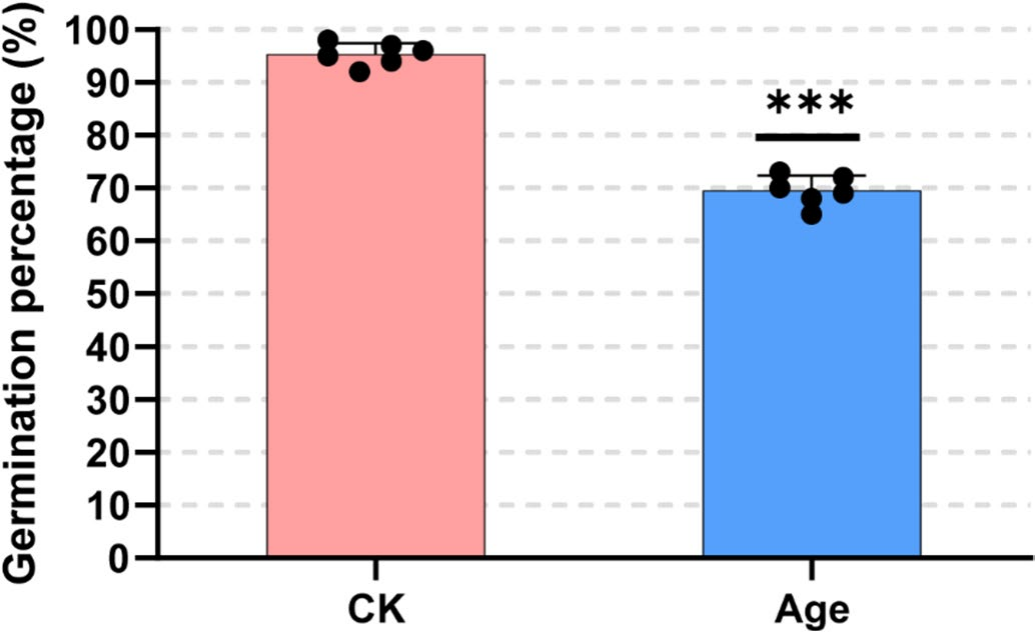
Effect of aging treatments on alfalfa seed germination

Alfalfa (cv. Zhongmu No.1) seeds were selected for uniform shape as the experiment materials in this study. Seed aging treatment was carried out following the established methodology outlined by Xia et al. [42]. Briefly, seeds pre-adjusted to 10% moisture content on a fresh-weight basis were immediately sealed in an aluminum foil bag (0.12 × 0.17 m2, approx. 40 g in each bag) at 45 °C in a water bath. The germination percentage declined to 70% on the 10th day (Fig. 8). The 0 d treatment was marked as the control (CK). Both seed samples for CK and aged for 10 d (Age) were collected at a certain time after imbibition, including 6, 12, 24 and 36 h. Each sample comprised four biological replicates, and each replicate consisted of seeds collected from at least 10 individual seeds. Following collection, the samples were rapidly frozen and ground to a fine powder using liquid nitrogen to preserve the molecular integrity.

### RNA-Seq data validation

Based on RNA-Seq data, some *MsWRKY* genes which exhibited significant induction in response to seed aging treatment were selected to validate the credibility of RNA-Seq data using an RT-qPCR assay. The total RNA of alfalfa seed was extracted by utilizing the RNA extraction Kit (Huayueyang Biotech Co., Ltd., China). Subsequently, the assay of inverse transcription was conducted using SuperMix with the qPCR Kit (TransGen Biotech, China). The qRT-PCR was conducted on a CFX96 Real-Time System. The cycle program employed for qRT-PCR consisted of an initial step at 95 °C for 3 min, followed by 40 cycles of 95 °C for 15 s, and 60 °C for 30 s. RT-qPCR specific primers were designed by Primer Premier 6.0 software (Premier Biosoft International, Palo Alto, CA, USA), and detailed information is shown in Table S9. The expression levels of *MsWRKY* genes were calculated using the 2^−ΔΔCt^ method [43]. The results were visualized using GraphPad Prism version 8.0. Accessed 10 May 2023.

### Supplementary Information

**Additional file 1:** **Table S1. **List of the *MsWRKY* genes name and ID in alfalfa.

**Additional file 2:** **Table S2. **List of the *MsWRKY* genes duplication events.

**Additional file 3:** **Table S3.** The collinearity relationships of the *MsWRKY* genes in four species

**Additional file 4:** **Table S4. **Protein property of MsWRKY proteins.

**Additional file 5:** **Table S5.** The structural features of motif 1-8.

**Additional file 6:** **Table S6.** Cis-regulatory elements in the promoters of MsWRKY gene families.

**Additional file 7:** **Table S7.** The secondary structure of MsWRKY proteins.

**Additional file 8:** **Table S8.** Expression (FPKM) of *MsWRKY* genes in different tissues.

**Additional file 9:** **Table S9.** Sequences of primers used in RT-qPCR.

## Data Availability

Data are contained within the article and Supplementary Materials. Raw sequencing data of the transcriptome used in the current study are available in the NCBI’s Sequence Read Archive (SRA, https://www.ncbi.nlm.nih.gov/sra) under the BioProject PRJNA1012365. The genomic information of the Zhongmu No.1 alfalfa variety was retrieved from the figshare website (https://figshare.com/articles/dataset/Medicago_sativa_genome_and_annottion_files). The RNA-Seq data, downloaded from the Noble Research Institute database (https://www.alfalfatoolbox.org), were used to evaluate the transcript abundance profiles of *MsWRKY* encoding genes across seven tissues, namely, leaves, flowers, pre-elongated stems, elongated stems, roots, and nodules.

## Abbreviations

TFs: Transcription factors
ABA: Abscisic acid
GA: Gibberellin
HMM: Hidden Markov model
NJ: Neighbor-joining
FPKM: Fragments per kilobase of exon model per million mapped fragments
