# Supplementary material for: Genomic identification and expression profiling of WRKY genes in alfalfa (Medicago sativa) elucidate their responsiveness to seed vigor
Source: BMC Plant Biol. 2023 Nov 16;23:568. doi: 10.1186/s12870-023-04597-x (PMC10652462; doi:10.1186/s12870-023-04597-x)
Supplement: Supplementary file 1 — Additional file 1: Table S1. List of the MsWRKY genes name and ID in alfalfa. [file 12870_2023_4597_MOESM1_ESM.docx]

**Table S1: List of the *MsWRKY* genes name and ID in alfalfa**

| ***MsWRKY* name** | ***MsWRKY* gene ID** |
| --- | --- |
| *MtWRKY1* | MsG0080048036.01.T01 |
| *MtWRKY2* | MsG0080048967.01.T01 |
| *MtWRKY3* | MsG0080049021.01.T01 |
| *MtWRKY4* | MsG0180000474.01.T01 |
| *MtWRKY5* | MsG0180000525.01.T01 |
| *MtWRKY6* | MsG0180000526.01.T01 |
| *MtWRKY7* | MsG0180000738.01.T01 |
| *MtWRKY8* | MsG0180003898.01.T01 |
| *MtWRKY9* | MsG0180004240.01.T01 |
| *MtWRKY10* | MsG0180004365.01.T01 |
| *MtWRKY11* | MsG0180004777.01.T01 |
| *MtWRKY12* | MsG0280006932.01.T01 |
| *MtWRKY13* | MsG0280007272.01.T01 |
| *MtWRKY14* | MsG0280007369.01.T01 |
| *MtWRKY15* | MsG0280007391.01.T01 |
| *MtWRKY16* | MsG0280007412.01.T01 |
| *MtWRKY17* | MsG0280007413.01.T01 |
| *MtWRKY18* | MsG0280007786.01.T01 |
| *MtWRKY19* | MsG0280007840.01.T01 |
| *MtWRKY20* | MsG0280008601.01.T01 |
| *MtWRKY21* | MsG0280009986.01.T01 |
| *MtWRKY22* | MsG0280009987.01.T01 |
| *MtWRKY23* | MsG0280009989.01.T01 |
| *MtWRKY24* | MsG0280009990.01.T01 |
| *MtWRKY25* | MsG0280010417.01.T01 |
| *MtWRKY26* | MsG0280010588.01.T01 |
| *MtWRKY27* | MsG0280011473.01.T01 |
| *MtWRKY28* | MsG0380014238.01.T01 |
| *MtWRKY29* | MsG0380014401.01.T01 |
| *MtWRKY30* | MsG0380014920.01.T01 |
| *MtWRKY31* | MsG0380016708.01.T01 |
| *MtWRKY32* | MsG0380016765.01.T01 |
| *MtWRKY33* | MsG0380017296.01.T01 |
| *MtWRKY34* | MsG0380017368.01.T02 |
| *MtWRKY35* | MsG0380017553.01.T01 |
| *MtWRKY36* | MsG0480018188.01.T01 |
| *MtWRKY37* | MsG0480018500.01.T01 |
| *MtWRKY38* | MsG0480020316.01.T01 |
| *MtWRKY39* | MsG0480021643.01.T01 |
| *MtWRKY40* | MsG0480022099.01.T01 |
| *MtWRKY41* | MsG0480022120.01.T01 |
| *MtWRKY42* | MsG0480022721.01.T01 |
| *MtWRKY43* | MsG0480022760.01.T01 |
| *MtWRKY44* | MsG0480023102.01.T01 |
| *MtWRKY45* | MsG0480023383.01.T01 |
| *MtWRKY46* | MsG0480023394.01.T01 |
| *MtWRKY47* | MsG0480023599.01.T01 |
| *MtWRKY48* | MsG0480023675.01.T01 |
| *MtWRKY49* | MsG0580024796.01.T01 |
| *MtWRKY50* | MsG0580026252.01.T01 |
| *MtWRKY51* | MsG0580026322.01.T01 |
| *MtWRKY52* | MsG0580028140.01.T01 |
| *MtWRKY53* | MsG0580028530.01.T01 |
| *MtWRKY54* | MsG0580028539.01.T01 |
| *MtWRKY55* | MsG0580028541.01.T01 |
| *MtWRKY56* | MsG0580028547.01.T01 |
| *MtWRKY57* | MsG0580028553.01.T02 |
| *MtWRKY58* | MsG0580028560.01.T01 |
| *MtWRKY59* | MsG0580028889.01.T01 |
| *MtWRKY60* | MsG0580029904.01.T01 |
| *MtWRKY61* | MsG0680031870.01.T01 |
| *MtWRKY62* | MsG0680032825.01.T01 |
| *MtWRKY63* | MsG0680032826.01.T01 |
| *MtWRKY64* | MsG0680033446.01.T01 |
| *MtWRKY65* | MsG0680035649.01.T01 |
| *MtWRKY66* | MsG0780036199.01.T01 |
| *MtWRKY67* | MsG0780036361.01.T01 |
| *MtWRKY68* | MsG0780037590.01.T01 |
| *MtWRKY69* | MsG0780037760.01.T01 |
| *MtWRKY70* | MsG0780038789.01.T01 |
| *MtWRKY71* | MsG0780039334.01.T01 |
| *MtWRKY72* | MsG0780039432.01.T01 |
| *MtWRKY73* | MsG0780039433.01.T01 |
| *MtWRKY74* | MsG0780039770.01.T01 |
| *MtWRKY75* | MsG0780039773.01.T03 |
| *MtWRKY76* | MsG0780039824.01.T01 |
| *MtWRKY77* | MsG0780039825.01.T01 |
| *MtWRKY78* | MsG0780040890.01.T01 |
| *MtWRKY79* | MsG0780041366.01.T01 |
| *MtWRKY80* | MsG0780041380.01.T01 |
| *MtWRKY81* | MsG0780041425.01.T01 |
| *MtWRKY82* | MsG0780041742.01.T01 |
| *MtWRKY83* | MsG0880041942.01.T01 |
| *MtWRKY84* | MsG0880043109.01.T01 |
| *MtWRKY85* | MsG0880045340.01.T01 |
| *MtWRKY86* | MsG0880045907.01.T01 |
| *MtWRKY87* | MsG0880046429.01.T01 |
| *MtWRKY88* | MsG0880047174.01.T01 |
| *MtWRKY89* | MsG0880047271.01.T01 |
| *MtWRKY90* | MsG0880047597.01.T01 |
| *MtWRKY91* | MsG0880047665.01.T01 |
