# Supplementary material for: Genomic identification and expression profiling of WRKY genes in alfalfa (Medicago sativa) elucidate their responsiveness to seed vigor
Source: BMC Plant Biol. 2023 Nov 16;23:568. doi: 10.1186/s12870-023-04597-x (PMC10652462; doi:10.1186/s12870-023-04597-x)
Supplement: Supplementary file 2 — Additional file 2: Table S2. List of the MsWRKY genes duplication events. [file 12870_2023_4597_MOESM2_ESM.docx]

**Table S2. List of the *MsWRKY* genes duplication events**

| **Tandem duplication** | | | | | |
| --- | --- | --- | --- | --- | --- |
| **Chr** | **genes name and ID** | **genes name** | **Chr** | **genes name and ID** | **genes name** |
| Ms-Chr1 | MsG0180004240.01 | MsWRKY9 | Ms-Chr1 | MsG0180004365.01 | MsWRKY10 |
| Ms-Chr2 | MsG0280007272.01 | MsWRKY13 | Ms-Chr2 | MsG0280007391.01 | MsWRKY15 |
| Ms-Chr2 | MsG0280007369.01 | MsWRKY14 | Ms-Chr2 | MsG0280007391.01 | MsWRKY15 |
| Ms-Chr2 | MsG0280007272.01 | MsWRKY13 | Ms-Chr2 | MsG0280007369.01 | MsWRKY14 |

| **Segmental duplication** | | | | | |
| --- | --- | --- | --- | --- | --- |
| **Chr** | **genes name and ID** | **genes name** | **Chr** | **genes name and ID** | **genes name** |
| Ms-Chr1 | MsG0180000525.01 | MsWRKY5 | Ms-Chr3 | MsG0380017368.01 | MsWRKY34 |
| Ms-Chr1 | MsG0180000738.01 | MsWRKY7 | Ms-Chr3 | MsG0380017553.01 | MsWRKY35 |
| Ms-Chr1 | MsG0180000474.01 | MsWRKY4 | Ms-Chr3 | MsG0380017296.01 | MsWRKY33 |
| Ms-Chr1 | MsG0180004777.01 | MsWRKY11 | Ms-Chr7 | MsG0780041380.01 | MsWRKY80 |
| Ms-Chr2 | MsG0280006932.01 | MsWRKY12 | Ms-Chr4 | MsG0480023102.01 | MsWRKY44 |
| Ms-Chr2 | MsG0280011473.01 | MsWRKY27 | Ms-Chr4 | MsG0480018188.01 | MsWRKY36 |
| Ms-Chr2 | MsG0280007391.01 | MsWRKY15 | Ms-Chr8 | MsG0880047665.01 | MsWRKY91 |
| Ms-Chr2 | MsG0280007272.01 | MsWRKY13 | Ms-Chr8 | MsG0880047665.01 | MsWRKY91 |
| Ms-Chr2 | MsG0280007840.01 | MsWRKY19 | Ms-Chr8 | MsG0880047174.01 | MsWRKY88 |
| Ms-Chr2 | MsG0280008601.01 | MsWRKY20 | Ms-Chr8 | MsG0880047271.01 | MsWRKY89 |
| Ms-Chr3 | MsG0380016765.01 | MsWRKY32 | Ms-Chr4 | MsG0480023394.01 | MsWRKY46 |
| Ms-Chr3 | MsG0380014401.01 | MsWRKY29 | Ms-Chr4 | MsG0480021643.01 | MsWRKY39 |
| Ms-Chr3 | MsG0380014401.01 | MsWRKY29 | Ms-Chr5 | MsG0580029904.01 | MsWRKY60 |
| Ms-Chr4 | MsG0480022120.01 | MsWRKY41 | Ms-Chr5 | MsG0580026252.01 | MsWRKY50 |
| Ms-Chr4 | MsG0480018500.01 | MsWRKY37 | Ms-Chr7 | MsG0780039334.01 | MsWRKY71 |
| Ms-Chr5 | MsG0580026252.01 | MsWRKY50 | Ms-Chr7 | MsG0780036361.01 | MsWRKY67 |
| Ms-Chr5 | MsG0580024796.01 | MsWRKY49 | Ms-Chr8 | MsG0880045907.01 | MsWRKY86 |
| Ms-Chr7 | MsG0780039770.01 | MsWRKY74 | Ms-Chr8 | MsG0880043109.01 | MsWRKY84 |
