# Supplementary material for: Genomic identification and expression profiling of WRKY genes in alfalfa (Medicago sativa) elucidate their responsiveness to seed vigor
Source: BMC Plant Biol. 2023 Nov 16;23:568. doi: 10.1186/s12870-023-04597-x (PMC10652462; doi:10.1186/s12870-023-04597-x)
Supplement: Supplementary file 3 — Additional file 3: Table S3. The collinearity relationships of the MsWRKY genes in four species [file 12870_2023_4597_MOESM3_ESM.docx]

**Table S3: The collinearity relationships of the *MsWRKY* genes in four species**

| ***M. truncatula* and *M. sativa*** | | | | |
| --- | --- | --- | --- | --- |
| Chr8 | MsG0880047665.01.T01 | == | Chr2 | rna-XM_013607417.3 |
| Chr8 | MsG0880047271.01.T01 | == | Chr2 | rna-XM_003595383.4 |
| Chr8 | MsG0880047174.01.T01 | == | Chr2 | rna-XM_003594666.4 |
| Chr8 | MsG0880047174.01.T01 | == | Chr4 | rna-XM_003609749.4 |
| Chr8 | MsG0880047597.01.T01 | == | Chr4 | rna-XM_003610274.4 |
| Chr8 | MsG0880047665.01.T01 | == | Chr4 | rna-XM_003610415.4 |
| Chr8 | MsG0880046429.01.T01 | == | Chr4 | rna-XM_003608971.4 |
| Chr8 | MsG0880045907.01.T01 | == | Chr4 | rna-XM_013601992.3 |
| Chr8 | MsG0880045907.01.T01 | == | Chr5 | rna-XM_003611628.4 |
| Chr8 | MsG0880043109.01.T01 | == | Chr7 | rna-XM_003624033.4 |
| Chr8 | MsG0880043109.01.T01 | == | Chr8 | rna-XM_013589319.2 |
| Chr8 | MsG0880041942.01.T01 | == | Chr8 | rna-XM_013588418.3 |
| Chr8 | MsG0880043109.01.T01 | == | Chr8 | rna-XM_013591658.3 |
| Chr7 | MsG0780041380.01.T01 | == | Chr1 | rna-XM_003591333.4 |
| Chr7 | MsG0780041742.01.T01 | == | Chr3 | rna-XM_003602147.4 |
| Chr7 | MsG0780039334.01.T01 | == | Chr4 | rna-XM_013599330.3 |
| Chr7 | MsG0780036361.01.T01 | == | Chr5 | rna-XM_003613895.4 |
| Chr7 | MsG0780040890.01.T01 | == | Chr7 | rna-XM_024770116.2 |
| Chr7 | MsG0780041366.01.T01 | == | Chr7 | rna-XM_003625929.4 |
| Chr7 | MsG0780041380.01.T01 | == | Chr7 | rna-XM_003625946.4 |
| Chr7 | MsG0780041425.01.T01 | == | Chr7 | rna-XM_003626025.4 |
| Chr7 | MsG0780041742.01.T01 | == | Chr7 | rna-XM_003626511.4 |
| Chr7 | MsG0780038789.01.T01 | == | Chr7 | rna-XM_013593426.3 |
| Chr7 | MsG0780039770.01.T01 | == | Chr7 | rna-XM_003624033.4 |
| Chr7 | MsG0780039773.01.T03 | == | Chr7 | rna-XM_003624030.4 |
| Chr7 | MsG0780039824.01.T01 | == | Chr7 | rna-XM_039827381.1 |
| Chr7 | MsG0780036199.01.T01 | == | Chr7 | rna-XM_003621095.4 |
| Chr7 | MsG0780036361.01.T01 | == | Chr7 | rna-XM_003621269.4 |
| Chr7 | MsG0780039334.01.T01 | == | Chr7 | rna-XM_024770100.2 |
| Chr7 | MsG0780039432.01.T01 | == | Chr7 | rna-XM_003623586.4 |
| Chr7 | MsG0780037590.01.T01 | == | Chr7 | rna-XM_039827842.1 |
| Chr7 | MsG0780039770.01.T01 | == | Chr8 | rna-XM_013589319.2 |
| Chr7 | MsG0780041742.01.T01 | == | Chr8 | rna-XM_013589210.3 |
| Chr7 | MsG0780039432.01.T01 | == | Chr8 | rna-XM_003626630.3 |
| Chr5 | MsG0580028541.01.T01 | == | Chr3 | rna-XM_003599231.4 |
| Chr5 | MsG0580028560.01.T01 | == | Chr3 | rna-XM_039831315.1 |
| Chr5 | MsG0580029904.01.T01 | == | Chr3 | rna-XM_003600211.4 |
| Chr5 | MsG0580028140.01.T01 | == | Chr3 | rna-XM_013606040.3 |
| Chr5 | MsG0580026252.01.T01 | == | Chr4 | rna-XM_003607724.4 |
| Chr5 | MsG0580024796.01.T01 | == | Chr4 | rna-XM_013601992.3 |
| Chr5 | MsG0580024796.01.T01 | == | Chr5 | rna-XM_003611628.4 |
| Chr5 | MsG0580029904.01.T01 | == | Chr5 | rna-XM_003617647.4 |
| Chr5 | MsG0580026252.01.T01 | == | Chr5 | rna-XM_003613895.4 |
| Chr5 | MsG0580026322.01.T01 | == | Chr5 | rna-XM_039834840.1 |
| Chr5 | MsG0580028539.01.T01 | == | Chr5 | rna-XM_003615848.4 |
| Chr5 | MsG0580028553.01.T02 | == | Chr5 | rna-XM_039834169.1 |
| Chr5 | MsG0580028560.01.T01 | == | Chr5 | rna-XM_003615901.4 |
| Chr5 | MsG0580028140.01.T01 | == | Chr5 | rna-XM_003615367.3 |
| Chr4 | MsG0480023102.01.T01 | == | Chr2 | rna-XM_013607155.3 |
| Chr4 | MsG0480018188.01.T01 | == | Chr2 | rna-XM_013610149.3 |
| Chr4 | MsG0480023394.01.T01 | == | Chr3 | rna-XM_003602454.4 |
| Chr4 | MsG0480021643.01.T01 | == | Chr3 | rna-XM_003600211.4 |
| Chr4 | MsG0480018188.01.T01 | == | Chr4 | rna-XM_003604197.4 |
| Chr4 | MsG0480018500.01.T01 | == | Chr4 | rna-XM_013599330.3 |
| Chr4 | MsG0480020316.01.T01 | == | Chr4 | rna-XM_024780519.1 |
| Chr4 | MsG0480022721.01.T01 | == | Chr4 | rna-XM_039833951.1 |
| Chr4 | MsG0480022760.01.T01 | == | Chr4 | rna-XM_013601875.3 |
| Chr4 | MsG0480022120.01.T01 | == | Chr4 | rna-XM_003607724.4 |
| Chr4 | MsG0480023383.01.T01 | == | Chr8 | rna-XM_003630070.3 |
| Chr4 | MsG0480023394.01.T01 | == | Chr8 | rna-XM_003630083.4 |
| Chr4 | MsG0480023599.01.T01 | == | Chr8 | rna-XM_039828839.1 |
| Chr4 | MsG0480023675.01.T01 | == | Chr8 | rna-XM_013591724.3 |
| Chr4 | MsG0480023102.01.T01 | == | Chr8 | rna-XM_003629764.4 |
| Chr4 | MsG0480023675.01.T01 | == | Chr8 | rna-XM_013589210.3 |
| Chr3 | MsG0380017553.01.T01 | == | Chr1 | rna-XM_013610259.3 |
| Chr3 | MsG0380017553.01.T01 | == | Chr3 | rna-XM_003603499.3 |
| Chr3 | MsG0380017296.01.T01 | == | Chr3 | rna-XM_003603132.4 |
| Chr3 | MsG0380017368.01.T02 | == | Chr3 | rna-XM_003603252.4 |
| Chr3 | MsG0380014238.01.T01 | == | Chr3 | rna-XM_013604397.3 |
| Chr3 | MsG0380014401.01.T01 | == | Chr3 | rna-XM_003600211.4 |
| Chr3 | MsG0380016708.01.T01 | == | Chr3 | rna-XM_013606040.3 |
| Chr3 | MsG0380016765.01.T01 | == | Chr3 | rna-XM_003602454.4 |
| Chr3 | MsG0380014920.01.T01 | == | Chr3 | rna-XM_039831315.1 |
| Chr3 | MsG0380014920.01.T01 | == | Chr5 | rna-XM_003615901.4 |
| Chr3 | MsG0380014401.01.T01 | == | Chr5 | rna-XM_003617647.4 |
| Chr3 | MsG0380016708.01.T01 | == | Chr5 | rna-XM_003615365.3 |
| Chr3 | MsG0380016765.01.T01 | == | Chr8 | rna-XM_003630083.4 |
| Chr2 | MsG0280007840.01.T01 | == | Chr1 | rna-XM_003591333.4 |
| Chr2 | MsG0280010417.01.T01 | == | Chr2 | rna-XM_003596659.3 |
| Chr2 | MsG0280010588.01.T01 | == | Chr2 | rna-XM_003596902.4 |
| Chr2 | MsG0280007391.01.T01 | == | Chr2 | rna-XM_013607417.3 |
| Chr2 | MsG0280007412.01.T01 | == | Chr2 | rna-XM_013607443.2 |
| Chr2 | MsG0280007786.01.T01 | == | Chr2 | rna-XM_013607626.3 |
| Chr2 | MsG0280007840.01.T01 | == | Chr2 | rna-XM_003594666.4 |
| Chr2 | MsG0280009987.01.T01 | == | Chr2 | rna-XM_003596251.4 |
| Chr2 | MsG0280006932.01.T01 | == | Chr2 | rna-XM_013607155.3 |
| Chr2 | MsG0280007272.01.T01 | == | Chr2 | rna-XM_013607417.3 |
| Chr2 | MsG0280011473.01.T01 | == | Chr2 | rna-XM_013610149.3 |
| Chr2 | MsG0280008601.01.T01 | == | Chr2 | rna-XM_003595383.4 |
| Chr2 | MsG0280007369.01.T01 | == | Chr2 | rna-XM_013607417.3 |
| Chr2 | MsG0280007391.01.T01 | == | Chr4 | rna-XM_003610415.4 |
| Chr2 | MsG0280007840.01.T01 | == | Chr4 | rna-XM_003609749.4 |
| Chr2 | MsG0280010588.01.T01 | == | Chr4 | rna-XM_024780532.1 |
| Chr2 | MsG0280006932.01.T01 | == | Chr8 | rna-XM_003629764.4 |
| Chr1 | MsG0180004365.01.T01 | == | Chr1 | rna-XM_024781420.2 |
| Chr1 | MsG0180004777.01.T01 | == | Chr1 | rna-XM_003591333.4 |
| Chr1 | MsG0180003898.01.T01 | == | Chr1 | rna-XM_013613119.3 |
| Chr1 | MsG0180004240.01.T01 | == | Chr1 | rna-XM_013613408.3 |
| Chr1 | MsG0180000474.01.T01 | == | Chr1 | rna-XM_003588866.4 |
| Chr1 | MsG0180000525.01.T01 | == | Chr1 | rna-XM_003588783.4 |
| Chr1 | MsG0180000738.01.T01 | == | Chr1 | rna-XM_013610259.3 |
| Chr1 | MsG0180004777.01.T01 | == | Chr2 | rna-XM_003594666.4 |
| Chr1 | MsG0180000738.01.T01 | == | Chr3 | rna-XM_003603499.3 |
| Chr1 | MsG0180000525.01.T01 | == | Chr3 | rna-XM_003603252.4 |
| Chr1 | MsG0180000474.01.T01 | == | Chr3 | rna-XM_003603132.4 |
| Chr1 | MsG0180004777.01.T01 | == | Chr4 | rna-XM_003609749.4 |
| Chr1 | MsG0180004777.01.T01 | == | Chr7 | rna-XM_003625946.4 |

| ***M.sativa and Glycine max*** | | | | |
| --- | --- | --- | --- | --- |
| Chr8 | MsG0880045907.01.T01 | == | Chr1 | rna-XM_003517268.5 |
| Chr8 | MsG0880046429.01.T01 | == | Chr1 | rna-XM_014761980.2 |
| Chr8 | MsG0880043109.01.T01 | == | Chr1 | rna-XM_006573726.4 |
| Chr8 | MsG0880047597.01.T01 | == | Chr2 | rna-NM_001317552.1 |
| Chr8 | MsG0880046429.01.T01 | == | Chr2 | rna-XM_014764342.2 |
| Chr8 | MsG0880047271.01.T01 | == | Chr2 | rna-XM_041010094.1 |
| Chr8 | MsG0880041942.01.T01 | == | Chr3 | rna-XM_003521012.4 |
| Chr8 | MsG0880045907.01.T01 | == | Chr5 | rna-NM_001250742.2 |
| Chr8 | MsG0880046429.01.T01 | == | Chr5 | rna-XM_014775431.3 |
| Chr8 | MsG0880043109.01.T01 | == | Chr5 | rna-XM_006580488.4 |
| Chr8 | MsG0880046429.01.T01 | == | Chr6 | rna-XM_003528135.5 |
| Chr8 | MsG0880047665.01.T01 | == | Chr7 | rna-XM_003528645.5 |
| Chr8 | MsG0880041942.01.T01 | == | Chr7 | rna-XM_003528979.5 |
| Chr8 | MsG0880047174.01.T01 | == | Chr7 | rna-XM_003528565.5 |
| Chr8 | MsG0880043109.01.T01 | == | Chr8 | rna-XM_006584671.4 |
| Chr8 | MsG0880043109.01.T01 | == | Chr9 | rna-XM_003534491.5 |
| Chr8 | MsG0880047665.01.T01 | == | Chr9 | rna-NM_001361279.1 |
| Chr8 | MsG0880047174.01.T01 | == | Chr9 | rna-NM_001361358.1 |
| Chr8 | MsG0880043109.01.T01 | == | Chr11 | rna-XM_006591462.3 |
| Chr8 | MsG0880045907.01.T01 | == | Chr11 | rna-NM_001361262.1 |
| Chr8 | MsG0880047271.01.T01 | == | Chr13 | rna-XM_041008038.1 |
| Chr8 | MsG0880047665.01.T01 | == | Chr15 | rna-XM_041009986.1 |
| Chr8 | MsG0880047271.01.T01 | == | Chr15 | rna-XM_003546492.5 |
| Chr8 | MsG0880047174.01.T01 | == | Chr15 | rna-XM_003547306.5 |
| Chr8 | MsG0880043109.01.T01 | == | Chr16 | rna-XM_003548590.5 |
| Chr8 | MsG0880047174.01.T01 | == | Chr17 | rna-XM_003550805.4 |
| Chr8 | MsG0880047271.01.T01 | == | Chr17 | rna-XM_003550534.5 |
| Chr8 | MsG0880047597.01.T01 | == | Chr17 | rna-NM_001361357.1 |
| Chr8 | MsG0880046429.01.T01 | == | Chr17 | rna-XM_003550725.5 |
| Chr8 | MsG0880045907.01.T01 | == | Chr17 | rna-XM_003549996.5 |
| Chr8 | MsG0880047665.01.T01 | == | Chr17 | rna-XM_003549661.5 |
| Chr8 | MsG0880046429.01.T01 | == | Chr18 | rna-XM_003551676.5 |
| Chr8 | MsG0880043109.01.T01 | == | Chr18 | rna-NM_001250579.2 |
| Chr8 | MsG0880046429.01.T01 | == | Chr18 | rna-XM_014770787.3 |
| Chr8 | MsG0880046429.01.T01 | == | Chr19 | rna-XM_041012841.1 |
| Chr7 | MsG0780039334.01.T01 | == | Chr1 | rna-XM_006573325.4 |
| Chr7 | MsG0780036361.01.T01 | == | Chr2 | rna-XM_003520049.5 |
| Chr7 | MsG0780041425.01.T01 | == | Chr2 | rna-XM_041010094.1 |
| Chr7 | MsG0780041380.01.T01 | == | Chr2 | rna-NM_001255065.2 |
| Chr7 | MsG0780040890.01.T01 | == | Chr3 | rna-XM_006576935.4 |
| Chr7 | MsG0780041366.01.T01 | == | Chr3 | rna-NM_001361374.1 |
| Chr7 | MsG0780041380.01.T01 | == | Chr3 | rna-NM_001250493.2 |
| Chr7 | MsG0780041425.01.T01 | == | Chr3 | rna-XM_003521573.5 |
| Chr7 | MsG0780041742.01.T01 | == | Chr3 | rna-XM_003521756.5 |
| Chr7 | MsG0780039334.01.T01 | == | Chr3 | rna-XM_006576402.4 |
| Chr7 | MsG0780039432.01.T01 | == | Chr3 | rna-NM_001252891.2 |
| Chr7 | MsG0780041742.01.T01 | == | Chr4 | rna-XM_003523340.5 |
| Chr7 | MsG0780041742.01.T01 | == | Chr6 | rna-NM_001317715.1 |
| Chr7 | MsG0780038789.01.T01 | == | Chr7 | rna-XM_041017038.1 |
| Chr7 | MsG0780041742.01.T01 | == | Chr7 | rna-NM_001255746.2 |
| Chr7 | MsG0780036361.01.T01 | == | Chr7 | rna-XM_006583894.3 |
| Chr7 | MsG0780036361.01.T01 | == | Chr8 | rna-NM_001250726.2 |
| Chr7 | MsG0780039824.01.T01 | == | Chr9 | rna-XM_014762389.3 |
| Chr7 | MsG0780039773.01.T03 | == | Chr9 | rna-XM_003534488.5 |
| Chr7 | MsG0780039770.01.T01 | == | Chr9 | rna-XM_003534491.5 |
| Chr7 | MsG0780039432.01.T01 | == | Chr9 | rna-NM_001315509.1 |
| Chr7 | MsG0780039334.01.T01 | == | Chr9 | rna-XM_003534620.5 |
| Chr7 | MsG0780036199.01.T01 | == | Chr9 | rna-XM_003533499.5 |
| Chr7 | MsG0780041425.01.T01 | == | Chr10 | rna-XM_014763160.3 |
| Chr7 | MsG0780041380.01.T01 | == | Chr10 | rna-NM_001250509.1 |
| Chr7 | MsG0780039432.01.T01 | == | Chr16 | rna-XM_006599669.3 |
| Chr7 | MsG0780041742.01.T01 | == | Chr16 | rna-NM_001251748.2 |
| Chr7 | MsG0780039773.01.T03 | == | Chr16 | rna-XM_003548392.4 |
| Chr7 | MsG0780039770.01.T01 | == | Chr16 | rna-XM_003548590.5 |
| Chr7 | MsG0780039770.01.T01 | == | Chr18 | rna-NM_001250579.2 |
| Chr7 | MsG0780039773.01.T03 | == | Chr18 | rna-NM_001361408.1 |
| Chr7 | MsG0780039824.01.T01 | == | Chr18 | rna-XM_014770297.3 |
| Chr7 | MsG0780036361.01.T01 | == | Chr18 | rna-XM_006602870.3 |
| Chr7 | MsG0780039334.01.T01 | == | Chr18 | rna-XM_014771244.3 |
| Chr7 | MsG0780038789.01.T01 | == | Chr18 | rna-XM_003551432.5 |
| Chr7 | MsG0780039432.01.T01 | == | Chr18 | rna-NM_001250690.3 |
| Chr7 | MsG0780040890.01.T01 | == | Chr19 | rna-XM_026126950.2 |
| Chr7 | MsG0780041366.01.T01 | == | Chr19 | rna-NM_001361257.1 |
| Chr7 | MsG0780041380.01.T01 | == | Chr19 | rna-XM_003554509.5 |
| Chr7 | MsG0780041425.01.T01 | == | Chr19 | rna-XM_006604701.4 |
| Chr7 | MsG0780041742.01.T01 | == | Chr19 | rna-XM_003554714.5 |
| Chr7 | MsG0780036199.01.T01 | == | Chr19 | rna-XM_041012841.1 |
| Chr7 | MsG0780036361.01.T01 | == | Chr20 | rna-XM_003556662.4 |
| Chr5 | MsG0580026252.01.T01 | == | Chr1 | rna-XM_003517766.5 |
| Chr5 | MsG0580026322.01.T01 | == | Chr1 | rna-XM_006573077.4 |
| Chr5 | MsG0580024796.01.T01 | == | Chr1 | rna-XM_003517268.5 |
| Chr5 | MsG0580026252.01.T01 | == | Chr2 | rna-XM_003520049.5 |
| Chr5 | MsG0580026322.01.T01 | == | Chr2 | rna-NM_001250598.2 |
| Chr5 | MsG0580029904.01.T01 | == | Chr2 | rna-XM_003519570.5 |
| Chr5 | MsG0580028541.01.T01 | == | Chr2 | rna-XM_041013348.1 |
| Chr5 | MsG0580028560.01.T01 | == | Chr2 | rna-NM_001361373.1 |
| Chr5 | MsG0580024796.01.T01 | == | Chr5 | rna-NM_001250742.2 |
| Chr5 | MsG0580028140.01.T01 | == | Chr6 | rna-NM_001368716.1 |
| Chr5 | MsG0580026252.01.T01 | == | Chr7 | rna-XM_006583894.3 |
| Chr5 | MsG0580029904.01.T01 | == | Chr8 | rna-XM_003530736.5 |
| Chr5 | MsG0580024796.01.T01 | == | Chr11 | rna-NM_001361262.1 |
| Chr5 | MsG0580028541.01.T01 | == | Chr14 | rna-XM_006596846.1 |
| Chr5 | MsG0580028547.01.T01 | == | Chr14 | rna-XM_003544857.5 |
| Chr5 | MsG0580028560.01.T01 | == | Chr14 | rna-NM_001317594.1 |
| Chr5 | MsG0580029904.01.T01 | == | Chr14 | rna-NM_001249973.2 |
| Chr5 | MsG0580028140.01.T01 | == | Chr14 | rna-XM_003544213.5 |
| Chr5 | MsG0580024796.01.T01 | == | Chr17 | rna-XM_003549996.5 |
| Chr5 | MsG0580024796.01.T01 | == | Chr17 | rna-XM_041011507.1 |
| Chr5 | MsG0580029904.01.T01 | == | Chr18 | rna-XM_003552967.4 |
| Chr5 | MsG0580028560.01.T01 | == | Chr18 | rna-NM_001361407.1 |
| Chr5 | MsG0580026252.01.T01 | == | Chr20 | rna-XM_003556662.4 |
| Chr4 | MsG0480023675.01.T01 | == | Chr1 | rna-NM_001354195.1 |
| Chr4 | MsG0480022099.01.T01 | == | Chr1 | rna-XM_003517766.5 |
| Chr4 | MsG0480018500.01.T01 | == | Chr1 | rna-XM_006573325.4 |
| Chr4 | MsG0480022099.01.T01 | == | Chr2 | rna-XM_003520049.5 |
| Chr4 | MsG0480018500.01.T01 | == | Chr3 | rna-XM_006576402.4 |
| Chr4 | MsG0480023394.01.T01 | == | Chr4 | rna-XM_003523204.5 |
| Chr4 | MsG0480023383.01.T01 | == | Chr4 | rna-NM_001250398.2 |
| Chr4 | MsG0480022721.01.T01 | == | Chr5 | rna-XM_006579953.4 |
| Chr4 | MsG0480022760.01.T01 | == | Chr5 | rna-XM_003524062.4 |
| Chr4 | MsG0480023102.01.T01 | == | Chr5 | rna-XM_003524922.5 |
| Chr4 | MsG0480023383.01.T01 | == | Chr5 | rna-NM_001248384.2 |
| Chr4 | MsG0480023675.01.T01 | == | Chr5 | rna-XM_003525301.5 |
| Chr4 | MsG0480023383.01.T01 | == | Chr6 | rna-NM_001361404.1 |
| Chr4 | MsG0480023394.01.T01 | == | Chr6 | rna-XM_003526790.5 |
| Chr4 | MsG0480018188.01.T01 | == | Chr7 | rna-XM_003530217.5 |
| Chr4 | MsG0480022099.01.T01 | == | Chr7 | rna-XM_006583894.3 |
| Chr4 | MsG0480022721.01.T01 | == | Chr8 | rna-XM_006584934.3 |
| Chr4 | MsG0480022760.01.T01 | == | Chr8 | rna-XM_003532600.5 |
| Chr4 | MsG0480023675.01.T01 | == | Chr8 | rna-NM_001354170.1 |
| Chr4 | MsG0480023102.01.T01 | == | Chr8 | rna-XM_006585112.4 |
| Chr4 | MsG0480023599.01.T01 | == | Chr8 | rna-NM_001250081.2 |
| Chr4 | MsG0480023383.01.T01 | == | Chr8 | rna-NM_001250384.2 |
| Chr4 | MsG0480023394.01.T01 | == | Chr8 | rna-NM_001250413.2 |
| Chr4 | MsG0480018188.01.T01 | == | Chr8 | rna-NM_001250629.2 |
| Chr4 | MsG0480018188.01.T01 | == | Chr13 | rna-XM_003543654.5 |
| Chr4 | MsG0480018188.01.T01 | == | Chr15 | rna-NM_001250463.2 |
| Chr4 | MsG0480023675.01.T01 | == | Chr19 | rna-XM_003554714.5 |
| Chr4 | MsG0480022099.01.T01 | == | Chr20 | rna-XM_003556662.4 |
| Chr4 | MsG0480022120.01.T01 | == | Chr20 | rna-XM_003556662.4 |
| Chr3 | MsG0380014920.01.T01 | == | Chr2 | rna-NM_001361373.1 |
| Chr3 | MsG0380014238.01.T01 | == | Chr2 | rna-XM_006576060.4 |
| Chr3 | MsG0380014401.01.T01 | == | Chr2 | rna-XM_003519570.5 |
| Chr3 | MsG0380017296.01.T01 | == | Chr4 | rna-XM_003522227.4 |
| Chr3 | MsG0380017368.01.T02 | == | Chr4 | rna-XM_026128174.2 |
| Chr3 | MsG0380017553.01.T01 | == | Chr4 | rna-NM_001361385.1 |
| Chr3 | MsG0380016765.01.T01 | == | Chr4 | rna-XM_003523204.5 |
| Chr3 | MsG0380016708.01.T01 | == | Chr4 | rna-XM_014774905.3 |
| Chr3 | MsG0380017296.01.T01 | == | Chr6 | rna-XM_003527704.5 |
| Chr3 | MsG0380017368.01.T02 | == | Chr6 | rna-NM_001254057.3 |
| Chr3 | MsG0380016708.01.T01 | == | Chr6 | rna-NM_001368716.1 |
| Chr3 | MsG0380016765.01.T01 | == | Chr6 | rna-XM_003526790.5 |
| Chr3 | MsG0380017553.01.T01 | == | Chr6 | rna-NM_001251732.2 |
| Chr3 | MsG0380014401.01.T01 | == | Chr8 | rna-XM_003530736.5 |
| Chr3 | MsG0380014238.01.T01 | == | Chr8 | rna-XM_006586511.4 |
| Chr3 | MsG0380016765.01.T01 | == | Chr8 | rna-NM_001250413.2 |
| Chr3 | MsG0380014920.01.T01 | == | Chr11 | rna-NM_001361274.1 |
| Chr3 | MsG0380017368.01.T02 | == | Chr13 | rna-XM_003543654.5 |
| Chr3 | MsG0380017553.01.T01 | == | Chr14 | rna-NM_001361364.1 |
| Chr3 | MsG0380014920.01.T01 | == | Chr14 | rna-NM_001317594.1 |
| Chr3 | MsG0380014401.01.T01 | == | Chr14 | rna-NM_001249973.2 |
| Chr3 | MsG0380016708.01.T01 | == | Chr14 | rna-XM_003544213.5 |
| Chr3 | MsG0380017296.01.T01 | == | Chr17 | rna-XM_006601158.4 |
| Chr3 | MsG0380017553.01.T01 | == | Chr17 | rna-XM_003550084.5 |
| Chr3 | MsG0380014920.01.T01 | == | Chr18 | rna-NM_001361407.1 |
| Chr3 | MsG0380014238.01.T01 | == | Chr18 | rna-XM_006602123.4 |
| Chr3 | MsG0380014401.01.T01 | == | Chr18 | rna-XM_003552967.4 |
| Chr2 | MsG0280006932.01.T01 | == | Chr5 | rna-XM_003524922.5 |
| Chr2 | MsG0280010588.01.T01 | == | Chr6 | rna-XM_003526288.5 |
| Chr2 | MsG0280007272.01.T01 | == | Chr7 | rna-XM_003528645.5 |
| Chr2 | MsG0280007840.01.T01 | == | Chr7 | rna-XM_003528565.5 |
| Chr2 | MsG0280007391.01.T01 | == | Chr7 | rna-XM_003528645.5 |
| Chr2 | MsG0280011473.01.T01 | == | Chr7 | rna-XM_003530217.5 |
| Chr2 | MsG0280011473.01.T01 | == | Chr8 | rna-NM_001250629.2 |
| Chr2 | MsG0280006932.01.T01 | == | Chr8 | rna-XM_006585112.4 |
| Chr2 | MsG0280007391.01.T01 | == | Chr9 | rna-NM_001361279.1 |
| Chr2 | MsG0280007786.01.T01 | == | Chr9 | rna-XM_003534748.5 |
| Chr2 | MsG0280007840.01.T01 | == | Chr9 | rna-NM_001361358.1 |
| Chr2 | MsG0280008601.01.T01 | == | Chr9 | rna-XM_003533771.5 |
| Chr2 | MsG0280007272.01.T01 | == | Chr9 | rna-NM_001361279.1 |
| Chr2 | MsG0280007369.01.T01 | == | Chr9 | rna-NM_001361279.1 |
| Chr2 | MsG0280010588.01.T01 | == | Chr12 | rna-XM_003540786.5 |
| Chr2 | MsG0280006932.01.T01 | == | Chr12 | rna-NM_001250658.2 |
| Chr2 | MsG0280010417.01.T01 | == | Chr12 | rna-NM_001250658.2 |
| Chr2 | MsG0280010417.01.T01 | == | Chr13 | rna-NM_001250797.2 |
| Chr2 | MsG0280011473.01.T01 | == | Chr13 | rna-XM_003543654.5 |
| Chr2 | MsG0280009986.01.T01 | == | Chr13 | rna-XM_006595371.4 |
| Chr2 | MsG0280008601.01.T01 | == | Chr13 | rna-XM_041008038.1 |
| Chr2 | MsG0280010588.01.T01 | == | Chr13 | rna-XM_003541905.5 |
| Chr2 | MsG0280006932.01.T01 | == | Chr13 | rna-NM_001250797.2 |
| Chr2 | MsG0280007391.01.T01 | == | Chr15 | rna-XM_041009986.1 |
| Chr2 | MsG0280007786.01.T01 | == | Chr15 | rna-XM_041010014.1 |
| Chr2 | MsG0280007840.01.T01 | == | Chr15 | rna-XM_003547306.5 |
| Chr2 | MsG0280007272.01.T01 | == | Chr15 | rna-XM_041009986.1 |
| Chr2 | MsG0280008601.01.T01 | == | Chr15 | rna-XM_003546492.5 |
| Chr2 | MsG0280011473.01.T01 | == | Chr15 | rna-NM_001250463.2 |
| Chr2 | MsG0280007369.01.T01 | == | Chr15 | rna-XM_041009986.1 |
| Chr2 | MsG0280007840.01.T01 | == | Chr17 | rna-XM_003550805.4 |
| Chr2 | MsG0280007272.01.T01 | == | Chr17 | rna-XM_003549661.5 |
| Chr2 | MsG0280008601.01.T01 | == | Chr17 | rna-XM_003550534.5 |
| Chr2 | MsG0280007840.01.T01 | == | Chr19 | rna-XM_003554509.5 |
| Chr1 | MsG0180004777.01.T01 | == | Chr2 | rna-NM_001255065.2 |
| Chr1 | MsG0180003898.01.T01 | == | Chr2 | rna-NM_001250675.2 |
| Chr1 | MsG0180004777.01.T01 | == | Chr3 | rna-NM_001250493.2 |
| Chr1 | MsG0180003898.01.T01 | == | Chr3 | rna-XM_014773816.3 |
| Chr1 | MsG0180000738.01.T01 | == | Chr4 | rna-NM_001361385.1 |
| Chr1 | MsG0180000474.01.T01 | == | Chr4 | rna-XM_003522227.4 |
| Chr1 | MsG0180000738.01.T01 | == | Chr6 | rna-NM_001251732.2 |
| Chr1 | MsG0180000474.01.T01 | == | Chr6 | rna-XM_003527704.5 |
| Chr1 | MsG0180000525.01.T01 | == | Chr6 | rna-XM_041016290.1 |
| Chr1 | MsG0180004777.01.T01 | == | Chr9 | rna-NM_001361358.1 |
| Chr1 | MsG0180004777.01.T01 | == | Chr10 | rna-NM_001250509.1 |
| Chr1 | MsG0180004365.01.T01 | == | Chr10 | rna-XM_003536129.4 |
| Chr1 | MsG0180003898.01.T01 | == | Chr10 | rna-NM_001317712.1 |
| Chr1 | MsG0180000738.01.T01 | == | Chr14 | rna-NM_001361364.1 |
| Chr1 | MsG0180000525.01.T01 | == | Chr14 | rna-XM_014766943.3 |
| Chr1 | MsG0180000525.01.T01 | == | Chr17 | rna-XM_006601149.4 |
| Chr1 | MsG0180000474.01.T01 | == | Chr17 | rna-XM_006601158.4 |
| Chr1 | MsG0180000738.01.T01 | == | Chr17 | rna-XM_041011414.1 |
| Chr1 | MsG0180004777.01.T01 | == | Chr17 | rna-XM_003550805.4 |
| Chr1 | MsG0180004777.01.T01 | == | Chr19 | rna-XM_003554509.5 |
| Chr1 | MsG0180004365.01.T01 | == | Chr20 | rna-XM_003556378.5 |

| ***M.sativa and Oryza sativa*** | | | | |
| --- | --- | --- | --- | --- |
| Chr8 | MsG0880041942.01.T01 | == | Chr4 | rna-XM_015779547.2 |
| Chr8 | MsG0880047271.01.T01 | == | Chr5 | rna-XM_015782638.2 |
| Chr7 | MsG0780041425.01.T01 | == | Chr1 | rna-XM_026024818.1 |
| Chr7 | MsG0780039432.01.T01 | == | Chr1 | rna-XM_015786556.2 |
| Chr7 | MsG0780041742.01.T01 | == | Chr1 | rna-XM_015776833.2 |
| Chr7 | MsG0780039432.01.T01 | == | Chr3 | rna-XM_015774235.2 |
| Chr7 | MsG0780039432.01.T01 | == | Chr5 | rna-XM_015783418.2 |
| Chr7 | MsG0780039334.01.T01 | == | Chr5 | rna-XM_015784891.2 |
| Chr7 | MsG0780039432.01.T01 | == | Chr11 | rna-XM_015760947.2 |
| Chr7 | MsG0780039432.01.T01 | == | Chr12 | rna-XM_015764454.2 |
| Chr3 | MsG0380016708.01.T01 | == | Chr1 | rna-XM_015788626.2 |
| Chr3 | MsG0380014920.01.T01 | == | Chr1 | rna-XM_015764385.1 |
| Chr3 | MsG0380016708.01.T01 | == | Chr3 | rna-XM_015774235.2 |
| Chr3 | MsG0380016708.01.T01 | == | Chr11 | rna-XM_015760947.2 |
| Chr3 | MsG0380016708.01.T01 | == | Chr12 | rna-XM_015764454.2 |
| Chr2 | MsG0280006932.01.T01 | == | Chr1 | rna-XM_015792105.2 |
| Chr1 | MsG0180003898.01.T01 | == | Chr3 | rna-XM_015777433.2 |

| ***Medicago sativa and Arabidopsis thaliana*** | | | | |
| --- | --- | --- | --- | --- |
| Chr8 | MsG0880047665.01.T01 | == | Chr1 | rna-NM_104910.3 |
| Chr8 | MsG0880046429.01.T01 | == | Chr1 | rna-NM_001332376.1 |
| Chr8 | MsG0880046429.01.T01 | == | Chr1 | rna-NM_105649.3 |
| Chr8 | MsG0880045907.01.T01 | == | Chr2 | rna-NM_127896.3 |
| Chr8 | MsG0880047597.01.T01 | == | Chr4 | rna-NM_119241.6 |
| Chr8 | MsG0880043109.01.T01 | == | Chr4 | rna-NM_116355.3 |
| Chr8 | MsG0880047665.01.T01 | == | Chr4 | rna-NM_118328.4 |
| Chr8 | MsG0880047665.01.T01 | == | Chr4 | rna-NM_116683.3 |
| Chr8 | MsG0880047174.01.T01 | == | Chr5 | rna-NM_124329.3 |
| Chr7 | MsG0780041742.01.T01 | == | Chr2 | rna-NM_130204.3 |
| Chr7 | MsG0780041380.01.T01 | == | Chr2 | rna-NM_130294.4 |
| Chr7 | MsG0780039334.01.T01 | == | Chr2 | rna-NM_129404.4 |
| Chr7 | MsG0780041380.01.T01 | == | Chr3 | rna-NM_116099.3 |
| Chr7 | MsG0780041425.01.T01 | == | Chr4 | rna-NM_116402.4 |
| Chr7 | MsG0780039770.01.T01 | == | Chr4 | rna-NM_116355.3 |
| Chr5 | MsG0580026322.01.T01 | == | Chr1 | rna-NM_105598.3 |
| Chr5 | MsG0580026252.01.T01 | == | Chr1 | rna-NM_101262.3 |
| Chr5 | MsG0580028541.01.T01 | == | Chr1 | rna-NM_100573.4 |
| Chr5 | MsG0580024796.01.T01 | == | Chr2 | rna-NM_127896.3 |
| Chr5 | MsG0580028560.01.T01 | == | Chr2 | rna-NM_128578.4 |
| Chr5 | MsG0580026252.01.T01 | == | Chr2 | rna-NM_126385.4 |
| Chr5 | MsG0580029904.01.T01 | == | Chr4 | rna-NM_179119.5 |
| Chr4 | MsG0480018188.01.T01 | == | Chr1 | rna-NM_106732.4 |
| Chr4 | MsG0480022760.01.T01 | == | Chr1 | rna-NM_102726.3 |
| Chr4 | MsG0480022099.01.T01 | == | Chr1 | rna-NM_101262.3 |
| Chr4 | MsG0480023675.01.T01 | == | Chr4 | rna-NM_118512.3 |
| Chr4 | MsG0480023394.01.T01 | == | Chr4 | rna-NM_120101.3 |
| Chr4 | MsG0480023675.01.T01 | == | Chr4 | rna-NM_117177.3 |
| Chr4 | MsG0480022760.01.T01 | == | Chr5 | rna-NM_124005.5 |
| Chr3 | MsG0380017553.01.T01 | == | Chr2 | rna-NM_128018.3 |
| Chr3 | MsG0380014920.01.T01 | == | Chr2 | rna-NM_128578.4 |
| Chr3 | MsG0380016708.01.T01 | == | Chr2 | rna-NM_129637.3 |
| Chr3 | MsG0380016708.01.T01 | == | Chr3 | rna-NM_115498.4 |
| Chr3 | MsG0380017553.01.T01 | == | Chr4 | rna-NM_179228.2 |
| Chr2 | MsG0280011473.01.T01 | == | Chr1 | rna-NM_106732.4 |
| Chr2 | MsG0280007786.01.T01 | == | Chr1 | rna-NM_102802.2 |
| Chr2 | MsG0280007391.01.T01 | == | Chr4 | rna-NM_118328.4 |
| Chr2 | MsG0280007272.01.T01 | == | Chr4 | rna-NM_118328.4 |
| Chr2 | MsG0280007391.01.T01 | == | Chr4 | rna-NM_116683.3 |
| Chr2 | MsG0280007272.01.T01 | == | Chr4 | rna-NM_116683.3 |
| Chr2 | MsG0280007840.01.T01 | == | Chr5 | rna-NM_124329.3 |
| Chr1 | MsG0180004777.01.T01 | == | Chr2 | rna-NM_130294.4 |
| Chr1 | MsG0180000525.01.T01 | == | Chr2 | rna-NM_128058.4 |
| Chr1 | MsG0180000738.01.T01 | == | Chr2 | rna-NM_128018.3 |
| Chr1 | MsG0180004777.01.T01 | == | Chr3 | rna-NM_116099.3 |
| Chr1 | MsG0180000525.01.T01 | == | Chr4 | rna-NM_119329.4 |
| Chr1 | MsG0180000738.01.T01 | == | Chr4 | rna-NM_179228.2 |
