# Supplementary material for: Genomic identification and expression profiling of WRKY genes in alfalfa (Medicago sativa) elucidate their responsiveness to seed vigor
Source: BMC Plant Biol. 2023 Nov 16;23:568. doi: 10.1186/s12870-023-04597-x (PMC10652462; doi:10.1186/s12870-023-04597-x)
Supplement: Supplementary file 4 — Additional file 4: Table S4. Protein property of MsWRKY proteins. [file 12870_2023_4597_MOESM4_ESM.docx]

**Table S4. Protein property of MsWRKY proteins**

| **MsWRKY Name** | **Number of amino acid** | **Molecular weight Mw/Da** | **Theoretical PI** | **Number of negatively amino acid** | **Number of positively amino acid** | **Instability index** | **Aliphatic index** | **Grand average of hydropathicity** | **Subcellular localization** |
| --- | --- | --- | --- | --- | --- | --- | --- | --- | --- |
| MsWRKY1 | 616 | 67261.92 | 6.06 | 64 | 57 | 49.91 | 57.03 | -0.844 | Nuclear |
| MsWRKY2 | 274 | 31136.32 | 6.71 | 38 | 37 | 37.71 | 72.92 | -0.682 | Nuclear |
| MsWRKY3 | 417 | 46812.29 | 8.03 | 61 | 58 | 50.68 | 57.05 | -0.849 | Nuclear |
| MsWRKY4 | 167 | 19186.14 | 6.83 | 49 | 51 | 63.16 | 43.71 | -1.092 | Nuclear |
| MsWRKY5 | 305 | 34998.42 | 8.58 | 22 | 22 | 58.39 | 66.13 | -0.855 | Nuclear |
| MsWRKY6 | 251 | 28383.99 | 7.67 | 39 | 43 | 49.69 | 72.27 | -0.812 | Nuclear |
| MsWRKY7 | 340 | 36807.77 | 9.51 | 34 | 35 | 48.73 | 67.68 | -0.48 | Nuclear |
| MsWRKY8 | 360 | 40695.83 | 9.8 | 26 | 48 | 52.39 | 62.56 | -0.897 | Nuclear |
| MsWRKY9 | 71 | 7975.81 | 5.79 | 9 | 7 | 56.35 | 60.28 | -0.634 | Mitochondria |
| MsWRKY10 | 289 | 33121.98 | 8.01 | 31 | 33 | 30.73 | 77.58 | -0.234 | Mitochondria |
| MsWRKY11 | 327 | 36602.65 | 6.33 | 37 | 34 | 60.95 | 60.43 | -0.742 | Nuclear |
| MsWRKY12 | 210 | 23265.84 | 5.04 | 31 | 23 | 57.16 | 54.29 | -0.781 | Cytoplasmic |
| MsWRKY13 | 325 | 35817.2 | 8.68 | 34 | 38 | 41.36 | 66.31 | -0.639 | Nuclear |
| MsWRKY14 | 394 | 42990.55 | 8.59 | 26 | 29 | 42.49 | 77.36 | -0.325 | Nuclear |
| MsWRKY15 | 563 | 60713.11 | 7.7 | 48 | 49 | 43.12 | 60.2 | -0.692 | Nuclear |
| MsWRKY16 | 222 | 24863.3 | 7.14 | 33 | 24 | 52.54 | 77.25 | -0.46 | Nuclear |
| MsWRKY17 | 427 | 46772.2 | 5.9 | 55 | 44 | 45.27 | 71.92 | -0.409 | Chloroplast |
| MsWRKY18 | 476 | 52636.5 | 5.98 | 28 | 38 | 52.84 | 56.95 | -0.853 | Nuclear |
| MsWRKY19 | 301 | 33479.86 | 9.44 | 33 | 35 | 54.57 | 54.05 | -0.964 | Nuclear |
| MsWRKY20 | 248 | 28603.16 | 8.03 | 28 | 21 | 50.83 | 63.67 | -0.896 | Nuclear |
| MsWRKY21 | 219 | 25457.43 | 5.58 | 39 | 37 | 51.76 | 59.63 | -0.697 | Nuclear |
| MsWRKY22 | 305 | 34984.24 | 6.56 | 19 | 16 | 45.55 | 69.34 | -0.739 | Nuclear |
| MsWRKY23 | 165 | 19436.63 | 6.24 | 42 | 27 | 56.6 | 59.76 | -0.787 | Nuclear |
| MsWRKY24 | 276 | 31409.93 | 4.93 | 37 | 32 | 49.23 | 73.12 | -0.662 | Cytoplasmic |
| MsWRKY25 | 261 | 28858.26 | 5.85 | 41 | 38 | 59.94 | 59.73 | -0.706 | Nuclear |
| MsWRKY26 | 424 | 46305.42 | 6.13 | 36 | 43 | 43.41 | 63.58 | -0.717 | Nuclear |
| MsWRKY27 | 317 | 35556.98 | 8.97 | 127 | 99 | 57.24 | 59.31 | -0.815 | Nuclear |
| MsWRKY28 | 924 | 103343.08 | 5.36 | 35 | 36 | 45.19 | 75.98 | -0.417 | Nuclear |
| MsWRKY29 | 245 | 27501.6 | 7.69 | 56 | 51 | 35.97 | 62 | -0.92 | Nuclear |
| MsWRKY30 | 511 | 57129.49 | 6.29 | 50 | 38 | 57.24 | 49.92 | -1.018 | Nuclear |
| MsWRKY31 | 324 | 36893.94 | 5.28 | 29 | 31 | 48.95 | 72.1 | -0.743 | Nuclear |
| MsWRKY32 | 243 | 27797.21 | 8.31 | 23 | 12 | 50.23 | 58.15 | -0.844 | Nuclear |
| MsWRKY33 | 120 | 13828.25 | 4.3 | 13 | 26 | 25.01 | 77.83 | -0.664 | Chloroplast |
| MsWRKY34 | 149 | 17213.49 | 9.97 | 11 | 23 | 53.75 | 65.44 | -0.795 | Nuclear |
| MsWRKY35 | 130 | 14713.66 | 10.13 | 42 | 45 | 43.82 | 54.77 | -1.029 | Nuclear |
| MsWRKY36 | 314 | 35081.27 | 8.34 | 25 | 28 | 53.85 | 64.52 | -0.835 | Nuclear |
| MsWRKY37 | 209 | 23807.16 | 8.59 | 60 | 61 | 34.67 | 55.41 | -0.985 | Nuclear |
| MsWRKY38 | 405 | 46278.25 | 7.58 | 72 | 65 | 31.19 | 63.48 | -0.82 | Nuclear |
| MsWRKY39 | 639 | 69535.55 | 6.32 | 66 | 81 | 48.48 | 55.98 | -0.737 | Nuclear |
| MsWRKY40 | 712 | 78023.17 | 9 | 65 | 60 | 45.78 | 72.01 | -0.453 | Plasma membrane |
| MsWRKY41 | 522 | 57834.33 | 6.37 | 61 | 47 | 36.94 | 65.15 | -0.767 | Nuclear |
| MsWRKY42 | 445 | 49521.17 | 5.4 | 37 | 33 | 52.42 | 58.34 | -0.936 | Nuclear |
| MsWRKY43 | 349 | 39332.07 | 6.39 | 33 | 35 | 63.75 | 46.91 | -1.039 | Nuclear |
| MsWRKY44 | 243 | 27306.75 | 8.22 | 17 | 18 | 57.11 | 62.63 | -0.754 | Nuclear |
| MsWRKY45 | 120 | 13899.48 | 7.82 | 44 | 64 | 43.86 | 63.42 | -0.941 | Cytoplasmic |
| MsWRKY46 | 441 | 50293.64 | 9.48 | 10 | 20 | 28.77 | 73.58 | -0.522 | Nuclear |
| MsWRKY47 | 112 | 13071.11 | 9.8 | 45 | 33 | 62.82 | 63.48 | -0.747 | Nuclear |
| MsWRKY48 | 361 | 41202.34 | 5.27 | 22 | 37 | 58.47 | 53.19 | -0.884 | Nuclear |
| MsWRKY49 | 270 | 29829.94 | 9.48 | 50 | 50 | 46.42 | 70.04 | -0.554 | Nuclear |
| MsWRKY50 | 419 | 45831.46 | 7.25 | 32 | 31 | 48.8 | 52.36 | -0.944 | Nuclear |
| MsWRKY51 | 298 | 33728.9 | 6.86 | 35 | 29 | 43.5 | 65.4 | -0.502 | Nuclear |
| MsWRKY52 | 235 | 26546.76 | 5.46 | 92 | 89 | 38.57 | 73.49 | -0.66 | Nuclear |
| MsWRKY53 | 805 | 91896.71 | 6.75 | 48 | 34 | 41.07 | 89.24 | -0.155 | Plasma membrane |
| MsWRKY54 | 441 | 49381.63 | 5.39 | 115 | 111 | 55.13 | 91.27 | -0.041 | Plasma membrane |
| MsWRKY55 | 976 | 112051.1 | 6.64 | 105 | 91 | 40.63 | 87.18 | -0.177 | Plasma membrane |
| MsWRKY56 | 890 | 102048.07 | 5.96 | 33 | 37 | 40.61 | 92.47 | -0.006 | Plasma membrane |
| MsWRKY57 | 328 | 37613.26 | 8.66 | 53 | 50 | 45.63 | 95.95 | -0.107 | Chloroplast |
| MsWRKY58 | 578 | 64068.66 | 6.48 | 22 | 37 | 68.24 | 43.37 | -1.051 | Nuclear |
| MsWRKY59 | 200 | 23179.38 | 9.72 | 55 | 60 | 37.91 | 66.25 | -0.685 | Nuclear |
| MsWRKY60 | 548 | 59611.71 | 8.61 | 38 | 38 | 46.6 | 52.3 | -0.812 | Nuclear |
| MsWRKY61 | 285 | 32747.06 | 7.21 | 35 | 30 | 50.24 | 71.51 | -0.686 | Nuclear |
| MsWRKY62 | 295 | 33306.92 | 6.12 | 59 | 46 | 66.85 | 63.08 | -0.849 | Chloroplast |
| MsWRKY63 | 623 | 68445.11 | 5.55 | 8 | 12 | 54.82 | 64.33 | -0.558 | Nuclear |
| MsWRKY64 | 68 | 8189.77 | 8.96 | 42 | 40 | 56.61 | 96.03 | 0.047 | Nuclear |
| MsWRKY65 | 391 | 43459.72 | 6.67 | 22 | 30 | 49.97 | 63.63 | -0.652 | Nuclear |
| MsWRKY66 | 231 | 26074.8 | 9.15 | 59 | 57 | 60.8 | 69.26 | -0.618 | Nuclear |
| MsWRKY67 | 524 | 57470.67 | 6.72 | 49 | 49 | 68.7 | 54.35 | -0.87 | Nuclear |
| MsWRKY68 | 419 | 48203.8 | 7.1 | 48 | 45 | 41.01 | 96.71 | -0.177 | Cytoplasmic |
| MsWRKY69 | 416 | 48432.83 | 6.46 | 39 | 29 | 48.87 | 72.88 | -0.388 | Nuclear |
| MsWRKY70 | 296 | 33554.31 | 5.63 | 62 | 57 | 46.08 | 69.46 | -0.713 | Nuclear |
| MsWRKY71 | 548 | 61495.71 | 6.42 | 45 | 37 | 55.75 | 44.67 | -1.07 | Nuclear |
| MsWRKY72 | 286 | 32531.31 | 5.72 | 45 | 33 | 62.18 | 68.46 | -0.87 | Nuclear |
| MsWRKY73 | 282 | 32171.7 | 5.53 | 27 | 16 | 50.78 | 68.4 | -0.89 | Nuclear |
| MsWRKY74 | 146 | 15885.31 | 4.47 | 27 | 16 | 39.74 | 44.18 | -0.896 | Nuclear |
| MsWRKY75 | 178 | 20455.02 | 9.11 | 21 | 27 | 35.41 | 72.75 | -0.865 | Nuclear |
| MsWRKY76 | 667 | 74108.74 | 8.9 | 69 | 77 | 56.89 | 52.65 | -0.912 | Nuclear |
| MsWRKY77 | 803 | 88653.15 | 6.04 | 94 | 80 | 57.15 | 61.07 | -0.736 | Nuclear |
| MsWRKY78 | 347 | 39103.5 | 7.63 | 41 | 42 | 51.46 | 61.24 | -0.893 | Nuclear |
| MsWRKY79 | 288 | 32588.69 | 5.18 | 45 | 34 | 58.14 | 76.11 | -0.627 | Nuclear |
| MsWRKY80 | 296 | 33348.11 | 6.66 | 33 | 32 | 60.6 | 59.56 | -0.823 | Nuclear |
| MsWRKY81 | 411 | 44620.27 | 6.42 | 37 | 34 | 57.79 | 73.41 | -0.476 | Nuclear |
| MsWRKY82 | 333 | 38016.73 | 6.68 | 48 | 47 | 42.03 | 66.46 | -0.838 | Nuclear |
| MsWRKY83 | 174 | 19546.5 | 8.6 | 19 | 21 | 35.53 | 54.83 | -0.881 | Nuclear |
| MsWRKY84 | 208 | 22927.21 | 4.94 | 34 | 23 | 44.3 | 49.28 | -0.805 | Nuclear |
| MsWRKY85 | 495 | 55555.21 | 6.23 | 46 | 42 | 36.57 | 62.63 | -0.63 | Nuclear |
| MsWRKY86 | 336 | 36617.43 | 9.74 | 27 | 46 | 52.34 | 66.22 | -0.618 | Nuclear |
| MsWRKY87 | 616 | 67271.87 | 5.88 | 65 | 56 | 50.23 | 57.03 | -0.849 | Nuclear |
| MsWRKY88 | 431 | 47446.31 | 5.7 | 50 | 41 | 57.85 | 58.58 | -0.765 | Nuclear |
| MsWRKY89 | 307 | 34036.38 | 9.06 | 23 | 32 | 56.44 | 72.15 | -0.452 | Nuclear |
| MsWRKY90 | 533 | 57838.73 | 6.9 | 65 | 64 | 53.08 | 68.93 | -0.712 | Nuclear |
| MsWRKY91 | 481 | 52279.96 | 4.99 | 56 | 39 | 45.4 | 63.99 | -0.674 | Nuclear |
