# Supplementary material for: Genomic identification and expression profiling of WRKY genes in alfalfa (Medicago sativa) elucidate their responsiveness to seed vigor
Source: BMC Plant Biol. 2023 Nov 16;23:568. doi: 10.1186/s12870-023-04597-x (PMC10652462; doi:10.1186/s12870-023-04597-x)
Supplement: Supplementary file 5 — Additional file 5: Table S5. The structural features of motif 1-8. [file 12870_2023_4597_MOESM5_ESM.docx]

**Table S5: The structural features of motif 1-8**

| **Name** | **E-value** | **Sites** | **Width** | **Amino acid sequence** | **Logo** |
| --- | --- | --- | --- | --- | --- |
| Motif 1 | 2.8e-27 | 54 | 21 | WRKYGQKVVKGNPYPRSYYRC | 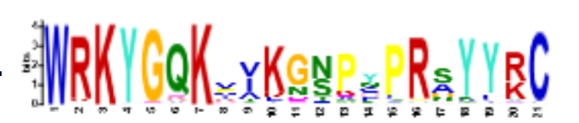 |
| Motif 2 | 3.6e-21 | 40 | 19 | RASEDPSIVITTYEGEHNH | 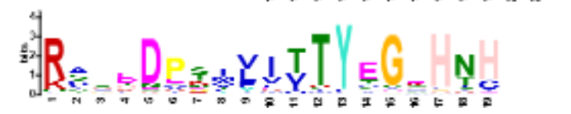 |
| Motif 3 | 1.0e-20 | 48 | 29 | KAKSZKTVREPRVVVQTRSEVDILDDGYR | 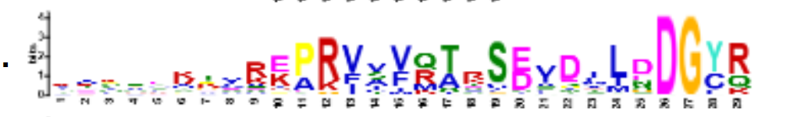 |
| Motif 4 | 1.6e-12 | 44 | 11 | APGCPVRKQVZ | 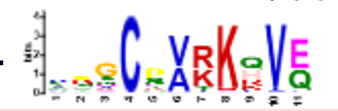 |
| Motif 5 | 4.6e-51 | 53 | 43 | KASDDGYNWRKYGQKQVKGSEFPRSYYKCTHPNCPVKKKVERS | 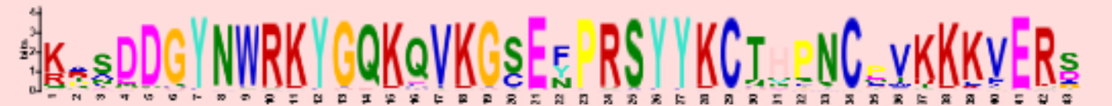 |
| Motif 6 | 4.3e-409 | 54 | 40 | LDGQITEIIYKGTHNHPKPQPSRRNSSSSSMSVSEERSSD | 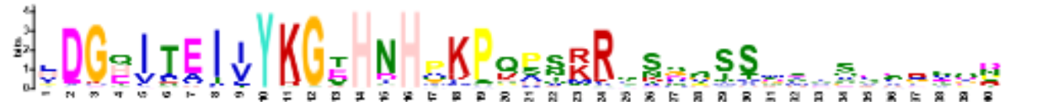 |
| Motif 7 | 3.3e-403 | 51 | 41 | QLEQLQTELKRVKSENKKLKZMLSEVTKNYTALQMQLVALM | 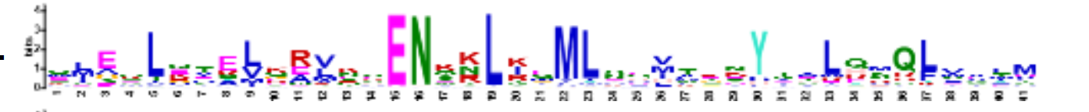 |
| Motif 8 | 7.4e-277 | 30 | 26 | VSAATAAITADPNFTAALAAAISSJI | 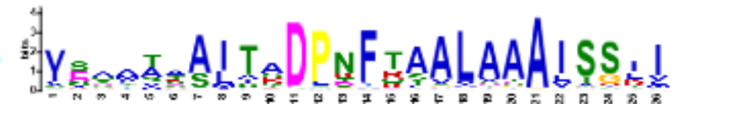 |
