# Supplementary material for: Genomic identification and expression profiling of WRKY genes in alfalfa (Medicago sativa) elucidate their responsiveness to seed vigor
Source: BMC Plant Biol. 2023 Nov 16;23:568. doi: 10.1186/s12870-023-04597-x (PMC10652462; doi:10.1186/s12870-023-04597-x)
Supplement: Supplementary file 6 — Additional file 6: Table S6. Cis-regulatory elements in the promoters of MsWRKY gene families. [file 12870_2023_4597_MOESM6_ESM.docx]

**Table S6: Cis-regulatory elements in the promoters of MsWRKY gene families**

|  |  | **light responsive element** | | | | | | | | | | | | | | | | |
| --- | --- | --- | --- | --- | --- | --- | --- | --- | --- | --- | --- | --- | --- | --- | --- | --- | --- | --- |
| *Cis*-acting element |  | Box II | G-box | GA-motif | GATA-motif | GT1-motif | Sp1 | TCT-motif | Box 4 | MRE | ACE | 3-AF1 binding site | ATCT-motif | LAMP-element | I-box | chs-CMA1a | ATC-motif | AE-box |
|  |  | part of a light responsive element | cis-acting element involved in light responsiveness | part of a light responsive element | part of a light responsive element | light responsive element | light responsive element | part of a light responsive element | part of a conserved  DNA module involved in light responsiveness | MYB binding  site involved in light responsiveness | cis-acting  element involved in light responsiveness | light responsive element | part of a  conserved DNA module involved in light responsiveness | part of a light  responsive element | part of a  light responsive element | part of a light responsive element | part of a conserved DNA module involved in light responsiveness | part of a  module for light response |
| *MsWRKY1* | MsG0080048036.01.T01 |  | 1 |  | 1 | 1 |  |  | 4 | 2 | 1 | 1 |  |  |  |  |  |  |
| *MsWRKY2* | MsG0080048967.01.T01 |  | 2 | 1 | 1 |  |  | 2 | 4 | 1 | 4 | 1 | 1 | 1 | 2 |  |  |  |
| *MsWRKY3* | MsG0080049021.01.T01 |  | 3 | 1 |  | 1 |  | 1 | 2 | 1 |  | 1 |  |  |  | 1 |  |  |
| *MsWRKY4* | MsG0180000474.01.T01 |  | 1 | 1 | 1 | 7 |  |  | 4 |  |  |  |  |  |  |  | 1 |  |
| *MsWRKY5* | MsG0180000525.01.T01 | 1 | 3 |  |  | 2 |  |  | 2 |  |  |  |  |  |  |  |  |  |
| *MsWRKY6* | MsG0180000526.01.T01 |  | 6 |  |  | 1 |  |  | 4 |  |  |  |  |  | 3 |  |  |  |
| *MsWRKY7* | MsG0180000738.01.T01 |  | 1 |  | 4 | 1 |  | 3 | 7 |  | 1 |  | 1 |  | 1 |  |  |  |
| *MsWRKY8* | MsG0180003898.01.T01 |  |  |  |  | 1 |  |  | 1 |  |  |  |  |  |  |  |  | 1 |
| *MsWRKY9* | MsG0180004240.01.T01 |  | 2 | 1 | 1 | 1 | 1 | 2 | 3 | 2 |  |  |  |  |  |  |  |  |
| *MsWRKY10* | MsG0180004365.01.T01 |  |  | 1 | 1 | 1 |  | 2 | 2 | 1 |  |  |  |  | 1 |  |  |  |
| *MsWRKY11* | MsG0180004777.01.T01 |  | 7 |  | 1 | 5 |  | 3 | 5 |  |  |  |  |  |  |  |  | 1 |
| *MsWRKY12* | MsG0280006932.01.T01 |  | 6 | 1 |  |  | 2 | 2 | 3 |  |  |  |  |  | 2 | 1 |  |  |
| *MsWRKY13* | MsG0280007272.01.T01 |  | 2 |  | 1 | 2 | 1 | 2 | 6 |  |  |  | 1 |  |  |  |  | 1 |
| *MsWRKY14* | MsG0280007369.01.T01 |  | 5 |  | 1 | 2 |  | 2 | 7 |  |  |  | 1 |  |  |  |  |  |
| *MsWRKY15* | MsG0280007391.01.T01 |  | 3 |  | 1 | 2 | 1 | 2 | 3 | 1 |  |  | 2 |  | 1 |  |  |  |
| *MsWRKY16* | MsG0280007412.01.T01 |  | 2 |  | 3 | 1 |  |  |  |  |  |  |  |  |  |  |  |  |
| *MsWRKY17* | MsG0280007413.01.T01 |  | 1 |  | 2 | 1 |  | 1 |  |  |  |  |  |  |  |  |  | 1 |
| *MsWRKY18* | MsG0280007786.01.T01 |  | 1 | 1 | 2 |  | 1 | 1 | 2 |  |  | 1 |  | 1 | 2 |  |  | 2 |
| *MsWRKY19* | MsG0280007840.01.T01 |  | 2 | 1 |  | 3 |  | 1 | 3 |  |  |  |  |  |  |  |  |  |
| *MsWRKY20* | MsG0280008601.01.T01 |  | 2 |  |  | 1 |  |  | 2 |  |  |  |  |  |  |  |  |  |
| *MsWRKY21* | MsG0280009986.01.T01 |  | 1 |  |  | 2 |  | 1 | 1 |  |  |  |  |  |  |  |  | 1 |
| *MsWRKY22* | MsG0280009987.01.T01 |  | 5 |  | 1 |  |  | 1 | 7 | 1 | 1 |  |  |  |  |  |  | 1 |
| *MsWRKY23* | MsG0280009989.01.T01 |  |  |  | 1 | 2 |  | 3 | 7 |  |  |  |  |  |  |  |  |  |
| *MsWRKY24* | MsG0280009990.01.T01 |  | 5 |  |  | 1 |  |  | 7 |  | 1 |  |  |  | 1 |  |  |  |
| *MsWRKY25* | MsG0280010417.01.T01 |  | 1 |  |  |  |  | 1 |  |  |  | 1 |  | 1 | 3 |  |  |  |
| *MsWRKY26* | MsG0280010588.01.T01 |  | 3 |  | 1 |  |  | 1 | 3 |  |  |  | 1 |  | 1 |  |  |  |
| *MsWRKY27* | MsG0280011473.01.T01 |  | 1 |  |  | 4 |  |  | 3 | 1 |  |  | 1 |  |  | 1 |  |  |
| *MsWRKY28* | MsG0380014238.01.T01 |  |  |  |  | 1 | 1 | 1 |  |  |  |  |  |  | 2 |  |  |  |
| *MsWRKY29* | MsG0380014401.01.T01 |  |  |  | 1 | 4 |  | 1 | 3 | 1 |  |  |  |  |  |  |  | 1 |
| *MsWRKY30* | MsG0380014920.01.T01 |  |  |  | 1 | 4 |  |  | 2 | 1 |  | 1 |  | 1 |  |  |  |  |
| *MsWRKY31* | MsG0380016708.01.T01 |  | 4 |  |  |  |  |  |  |  |  |  | 1 |  |  |  |  |  |
| *MsWRKY32* | MsG0380016765.01.T01 |  |  |  |  | 1 |  |  | 2 | 1 |  |  |  | 1 |  |  |  | 1 |
| *MsWRKY33* | MsG0380017296.01.T01 |  | 2 |  |  | 3 |  | 1 | 3 |  |  |  |  |  |  | 1 |  |  |
| *MsWRKY34* | MsG0380017368.01.T02 |  | 2 |  |  |  |  | 1 | 3 | 2 |  | 1 |  |  |  |  |  |  |
| *MsWRKY35* | MsG0380017553.01.T01 |  |  |  | 1 | 3 |  | 2 | 1 |  |  |  | 2 |  | 2 |  |  | 1 |
| *MsWRKY36* | MsG0480018188.01.T01 |  | 3 | 1 | 1 | 2 |  |  | 6 | 1 |  |  |  | 1 | 1 |  |  |  |
| *MsWRKY37* | MsG0480018500.01.T01 |  | 1 |  | 1 | 1 |  |  | 1 |  |  |  |  |  |  | 2 |  | 2 |
| *MsWRKY38* | MsG0480020316.01.T01 | 1 | 2 |  |  | 3 |  |  | 2 |  |  | 1 |  |  |  | 1 |  | 3 |
| *MsWRKY39* | MsG0480021643.01.T01 |  |  |  |  |  | 1 | 3 | 2 |  |  |  | 1 |  |  | 1 |  | 1 |
| *MsWRKY40* | MsG0480022099.01.T01 |  | 3 | 3 |  |  |  | 1 |  |  |  |  |  | 1 | 2 | 1 |  | 1 |
| *MsWRKY41* | MsG0480022120.01.T01 |  | 1 | 1 |  | 3 |  | 1 | 1 | 1 |  |  |  | 1 | 1 | 2 |  |  |
| *MsWRKY42* | MsG0480022721.01.T01 |  | 2 | 1 |  |  |  |  | 4 | 1 |  | 1 |  |  | 1 |  |  |  |
| *MsWRKY43* | MsG0480022760.01.T01 |  | 8 |  | 1 | 3 |  | 1 | 6 | 1 | 1 |  | 1 |  |  |  |  |  |
| *MsWRKY44* | MsG0480023102.01.T01 |  | 2 |  | 2 | 2 |  | 2 | 3 | 1 |  |  |  |  |  |  |  | 1 |
| *MsWRKY45* | MsG0480023383.01.T01 |  | 7 | 1 |  |  |  |  | 2 |  |  |  | 1 |  | 1 |  |  |  |
| *MsWRKY46* | MsG0480023394.01.T01 |  |  |  | 1 |  |  |  | 4 | 1 |  |  | 1 |  |  | 1 |  |  |
| *MsWRKY47* | MsG0480023599.01.T01 |  | 4 |  | 3 |  | 2 |  |  |  | 2 |  |  |  | 2 | 1 |  |  |
| *MsWRKY48* | MsG0480023675.01.T01 |  | 1 |  |  | 2 |  | 2 | 5 |  |  |  |  |  |  |  |  |  |
| *MsWRKY49* | MsG0580024796.01.T01 |  | 4 |  |  | 1 |  |  | 1 |  |  |  |  | 1 |  |  |  | 1 |
| *MsWRKY50* | MsG0580026252.01.T01 |  | 1 |  |  |  |  | 3 | 9 |  |  |  |  |  |  |  |  | 1 |
| *MsWRKY51* | MsG0580026322.01.T01 |  | 2 |  |  |  |  |  | 4 |  |  |  |  |  | 1 |  |  |  |
| *MsWRKY52* | MsG0580028140.01.T01 |  |  |  | 1 | 1 |  | 1 | 8 |  |  |  |  | 2 |  | 1 |  | 2 |
| *MsWRKY53* | MsG0580028530.01.T01 |  | 1 | 1 |  | 1 |  |  | 2 |  |  |  | 2 | 1 |  |  |  |  |
| *MsWRKY54* | MsG0580028539.01.T01 |  |  |  |  |  |  |  | 6 |  |  |  |  | 1 | 1 | 2 |  |  |
| *MsWRKY55* | MsG0580028541.01.T01 |  |  |  |  | 1 | 1 |  |  |  |  | 1 | 1 |  |  |  | 1 |  |
| *MsWRKY56* | MsG0580028547.01.T01 |  |  |  |  | 1 | 1 |  |  |  |  | 1 |  |  | 1 |  | 1 |  |
| *MsWRKY57* | MsG0580028553.01.T02 |  |  |  |  |  |  |  | 1 |  | 2 |  |  |  |  |  |  | 1 |
| *MsWRKY58* | MsG0580028560.01.T01 |  | 1 |  | 2 | 8 |  | 3 | 3 |  |  |  |  | 1 |  |  |  | 2 |
| *MsWRKY59* | MsG0580028889.01.T01 |  | 2 | 1 |  | 6 |  |  | 2 | 1 |  |  |  |  |  | 3 |  |  |
| *MsWRKY60* | MsG0580029904.01.T01 |  |  | 1 | 1 | 1 |  | 2 | 4 |  |  |  |  |  |  |  |  |  |
| *MsWRKY61* | MsG0680031870.01.T01 |  | 1 |  | 1 | 1 |  | 1 | 2 | 1 |  |  |  |  |  | 1 |  |  |
| *MsWRKY62* | MsG0680032825.01.T01 |  | 1 |  |  | 2 |  |  | 5 |  |  |  |  |  | 1 | 1 |  |  |
| *MsWRKY63* | MsG0680032826.01.T01 |  |  | 1 |  | 1 |  |  |  |  | 1 |  |  | 1 | 1 |  |  |  |
| *MsWRKY64* | MsG0680033446.01.T01 |  | 1 |  |  | 1 |  |  | 5 |  |  |  |  |  |  | 2 |  |  |
| *MsWRKY65* | MsG0680035649.01.T01 |  | 2 |  | 1 | 3 |  | 1 | 2 |  | 1 |  |  | 2 | 3 |  |  | 1 |
| *MsWRKY66* | MsG0780036199.01.T01 |  | 1 | 1 |  | 4 |  | 1 | 4 | 1 |  |  |  |  |  |  |  |  |
| *MsWRKY67* | MsG0780036361.01.T01 |  | 1 |  |  | 1 |  | 1 | 3 | 2 |  |  |  |  |  |  |  |  |
| *MsWRKY68* | MsG0780037590.01.T01 |  | 5 |  |  | 1 |  |  | 6 | 1 |  |  |  |  |  | 1 |  | 1 |
| *MsWRKY69* | MsG0780037760.01.T01 |  | 1 |  | 1 |  | 1 |  |  | 1 |  | 1 |  |  | 1 |  |  | 4 |
| *MsWRKY70* | MsG0780038789.01.T01 |  |  | 1 |  | 1 |  | 2 | 1 |  | 1 |  |  |  |  | 1 |  |  |
| *MsWRKY71* | MsG0780039334.01.T01 |  | 7 |  | 6 | 1 |  | 1 | 2 | 1 | 1 |  |  |  |  |  |  | 1 |
| *MsWRKY72* | MsG0780039432.01.T01 |  | 3 |  | 1 | 3 |  |  |  |  |  |  |  | 1 | 1 | 1 | 1 |  |
| *MsWRKY73* | MsG0780039433.01.T01 |  | 1 |  | 1 |  |  |  | 2 | 1 |  |  |  |  | 1 |  |  | 2 |
| *MsWRKY74* | MsG0780039770.01.T01 |  | 5 |  | 1 | 2 |  |  | 2 | 1 |  |  |  |  |  | 2 |  | 2 |
| *MsWRKY75* | MsG0780039773.01.T03 |  |  |  |  | 3 |  | 2 | 1 | 1 |  |  |  |  |  | 2 |  |  |
| *MsWRKY76* | MsG0780039824.01.T01 |  | 2 |  |  |  |  |  | 8 |  |  |  | 1 |  |  |  |  | 1 |
| *MsWRKY77* | MsG0780039825.01.T01 |  | 7 |  | 1 | 5 |  |  | 5 |  |  |  |  |  | 2 | 1 |  |  |
| *MsWRKY78* | MsG0780040890.01.T01 |  |  |  | 1 | 3 |  | 2 | 1 |  |  |  |  |  | 1 |  |  |  |
| *MsWRKY79* | MsG0780041366.01.T01 | 1 | 8 | 1 | 1 |  |  | 1 | 2 |  | 1 |  |  |  | 1 |  |  | 1 |
| *MsWRKY80* | MsG0780041380.01.T01 |  | 1 | 1 |  | 3 |  |  | 2 | 1 |  |  |  |  | 1 | 2 |  | 1 |
| *MsWRKY81* | MsG0780041425.01.T01 |  | 3 |  | 1 | 1 |  | 1 | 2 |  |  |  |  | 1 |  | 1 |  |  |
| *MsWRKY82* | MsG0780041742.01.T01 |  | 5 | 1 |  | 6 |  | 1 | 1 |  |  |  |  |  |  | 2 |  | 1 |
| *MsWRKY83* | MsG0880041942.01.T01 |  | 4 |  |  | 3 |  |  | 10 |  |  |  |  |  |  | 1 |  |  |
| *MsWRKY84* | MsG0880043109.01.T01 |  | 1 | 1 |  | 1 |  | 1 | 4 | 1 |  |  |  | 1 | 1 |  |  | 2 |
| *MsWRKY85* | MsG0880045340.01.T01 |  | 1 |  |  |  |  | 1 | 5 |  |  |  |  | 2 | 1 |  |  | 2 |
| *MsWRKY86* | MsG0880045907.01.T01 |  |  | 1 | 2 | 3 |  | 1 | 5 | 1 | 1 |  | 1 |  | 1 |  |  |  |
| *MsWRKY87* | MsG0880046429.01.T01 |  |  |  | 1 | 1 |  | 2 | 4 | 2 | 1 | 1 |  |  |  |  |  | 2 |
| *MsWRKY88* | MsG0880047174.01.T01 |  | 4 |  |  | 2 |  |  | 7 | 2 |  |  |  |  | 1 |  |  |  |
| *MsWRKY89* | MsG0880047271.01.T01 |  | 2 |  |  |  |  | 1 | 2 |  |  |  | 1 |  |  |  |  | 1 |
| *MsWRKY90* | MsG0880047597.01.T01 |  | 3 |  |  |  |  | 1 |  | 1 | 1 |  |  |  |  |  |  |  |
| *MsWRKY91* | MsG0880047665.01.T01 |  | 2 |  | 2 | 1 |  |  |  |  | 1 | 1 | 1 |  |  |  |  |  |

| Catergory |  | light responsive element | | | | | | | | | | | hormone responsive element | | | | | | | | | |
| --- | --- | --- | --- | --- | --- | --- | --- | --- | --- | --- | --- | --- | --- | --- | --- | --- | --- | --- | --- | --- | --- | --- |
| *Cis*-acting element |  | TCCC-motif | Gap-box | CAG-motif | AT1-motif | Pc-CMA2c | C-box | GTGGC-motif | 4cl-CMA1b | sbp-CMA1c | ACA-motif | AAAC-motif | ABRE | AuxRR-core | CGTCA-motif | GARE-motif | TCA-element | TGA-element | TGACG-motif | TATC-box | P-box | SARE |
|  |  | part  of a light responsive element | part of a  light responsive element | part of a light response element | part of a light responsive module | part of a light responsive element | cis-acting regulatory element involved in light responsiveness | part of a light responsive element | light responsive element | part of a light responsive element | part of  gapA in (gapA-CMA1) involved with light responsiveness | light  responsive element | cis-acting element involved in the abscisic acid responsiveness | cis-acting regulatory element involved in auxin responsiveness | cis-acting regulatory element involved in the MeJA-responsiveness | gibberellin-responsive element | cis-acting element involved in salicylic acid responsivenessethylene-responsive element | auxin-responsive element | cis-acting regulatory element involved in the MeJA-responsiveness | cis-acting  element involved in gibberellin-responsiveness | gibberellin- responsive element | cis-acting element involved in salicylic acid responsiveness |
| *MsWRKY1* | MsG0080048036.01.T01 |  |  |  |  |  |  |  |  |  |  |  | 1 |  | 2 |  |  |  | 2 |  |  |  |
| *MsWRKY2* | MsG0080048967.01.T01 |  |  |  |  |  |  |  |  |  |  |  | 2 |  | 2 |  | 1 |  | 2 | 1 |  |  |
| *MsWRKY3* | MsG0080049021.01.T01 |  |  |  |  |  |  |  |  |  |  |  | 2 | 1 | 2 | 1 | 1 |  | 2 | 2 |  |  |
| *MsWRKY4* | MsG0180000474.01.T01 |  |  |  |  |  |  |  |  |  |  |  | 1 |  | 1 |  | 3 |  | 1 |  |  |  |
| *MsWRKY5* | MsG0180000525.01.T01 |  |  |  |  |  |  |  |  |  |  |  | 2 |  | 2 | 1 |  | 1 | 2 | 1 |  |  |
| *MsWRKY6* | MsG0180000526.01.T01 |  |  |  |  |  |  |  |  |  |  |  | 5 |  | 1 |  |  |  | 1 |  |  |  |
| *MsWRKY7* | MsG0180000738.01.T01 |  |  |  |  |  |  |  |  |  |  |  | 1 |  | 1 |  | 1 | 1 | 1 |  |  |  |
| *MsWRKY8* | MsG0180003898.01.T01 |  |  |  |  |  |  |  |  |  |  |  |  |  |  | 5 |  |  |  |  |  |  |
| *MsWRKY9* | MsG0180004240.01.T01 |  |  |  |  |  |  |  |  |  |  |  | 1 |  | 2 |  |  |  | 2 |  |  |  |
| *MsWRKY10* | MsG0180004365.01.T01 |  |  |  |  |  |  |  |  |  | 1 | 1 |  |  | 1 |  |  |  | 1 |  | 1 |  |
| *MsWRKY11* | MsG0180004777.01.T01 |  |  |  |  |  |  |  |  |  |  |  | 4 |  |  |  | 1 | 2 |  | 1 |  |  |
| *MsWRKY12* | MsG0280006932.01.T01 |  |  |  |  |  |  |  |  |  |  |  | 4 |  | 2 |  |  |  | 2 |  | 1 |  |
| *MsWRKY13* | MsG0280007272.01.T01 | 1 | 1 |  |  |  |  |  |  |  |  |  | 5 | 1 | 2 | 1 | 1 |  | 2 | 1 | 1 |  |
| *MsWRKY14* | MsG0280007369.01.T01 |  |  |  |  |  |  |  |  |  |  |  | 3 |  | 1 |  | 1 |  | 1 | 1 |  |  |
| *MsWRKY15* | MsG0280007391.01.T01 | 1 |  |  |  |  |  |  |  |  |  |  | 3 |  | 1 |  | 19 |  | 1 | 1 | 1 |  |
| *MsWRKY16* | MsG0280007412.01.T01 |  |  |  |  |  |  |  |  |  |  |  | 2 |  |  |  | 3 |  |  | 2 |  |  |
| *MsWRKY17* | MsG0280007413.01.T01 |  | 2 |  |  |  |  |  |  |  |  |  | 1 |  | 1 |  | 2 |  | 1 |  |  |  |
| *MsWRKY18* | MsG0280007786.01.T01 | 1 |  | 1 |  |  |  |  |  |  |  |  | 1 |  |  |  |  | 2 |  | 1 | 1 |  |
| *MsWRKY19* | MsG0280007840.01.T01 | 1 |  |  | 1 |  |  |  |  |  |  |  | 2 |  | 1 |  | 1 |  | 1 |  |  |  |
| *MsWRKY20* | MsG0280008601.01.T01 |  |  |  |  |  |  |  |  |  |  |  | 2 |  | 2 |  |  |  | 2 |  |  |  |
| *MsWRKY21* | MsG0280009986.01.T01 |  |  |  |  |  |  |  |  |  |  |  |  |  | 1 |  |  |  | 1 |  |  |  |
| *MsWRKY22* | MsG0280009987.01.T01 | 1 |  |  |  |  |  |  |  |  |  |  | 5 |  | 1 |  | 2 |  | 1 | 2 |  |  |
| *MsWRKY23* | MsG0280009989.01.T01 | 1 |  |  |  |  |  |  |  |  |  |  |  |  | 2 | 1 |  | 1 | 2 |  |  |  |
| *MsWRKY24* | MsG0280009990.01.T01 |  |  |  |  |  |  |  |  |  |  |  | 5 |  |  |  |  |  |  | 1 |  |  |
| *MsWRKY25* | MsG0280010417.01.T01 |  |  |  |  |  |  |  |  |  |  |  | 1 |  | 1 |  |  | 1 | 1 |  |  |  |
| *MsWRKY26* | MsG0280010588.01.T01 | 1 |  |  |  |  |  |  |  |  |  |  | 2 |  | 2 |  | 1 |  | 2 |  |  |  |
| *MsWRKY27* | MsG0280011473.01.T01 |  |  |  |  |  |  |  |  |  |  |  | 2 |  |  |  | 1 |  |  |  |  |  |
| *MsWRKY28* | MsG0380014238.01.T01 |  |  |  |  |  |  |  |  |  |  |  | 1 |  | 1 |  |  |  | 1 |  |  |  |
| *MsWRKY29* | MsG0380014401.01.T01 | 1 |  |  |  |  |  |  |  |  |  |  |  |  | 1 | 1 |  |  | 1 |  | 1 |  |
| *MsWRKY30* | MsG0380014920.01.T01 |  |  |  |  |  |  |  |  |  |  |  |  |  | 2 |  | 1 | 1 | 2 |  | 1 |  |
| *MsWRKY31* | MsG0380016708.01.T01 | 1 |  |  |  |  |  |  |  |  |  |  | 3 |  | 5 |  | 4 | 1 | 5 |  |  |  |
| *MsWRKY32* | MsG0380016765.01.T01 |  | 1 |  | 1 |  |  |  |  |  |  |  |  |  |  |  |  | 1 |  | 2 |  |  |
| *MsWRKY33* | MsG0380017296.01.T01 |  |  |  |  |  |  |  |  |  |  |  | 2 |  | 1 |  |  |  | 1 |  |  |  |
| *MsWRKY34* | MsG0380017368.01.T02 | 1 |  |  |  |  |  |  |  |  |  | 1 | 1 |  | 2 |  | 1 | 1 | 2 |  |  |  |
| *MsWRKY35* | MsG0380017553.01.T01 |  |  |  |  |  |  |  |  |  |  |  |  | 2 | 3 |  |  |  | 3 |  |  |  |
| *MsWRKY36* | MsG0480018188.01.T01 |  |  |  |  |  |  |  |  |  |  |  | 3 |  |  |  |  |  |  | 2 | 2 |  |
| *MsWRKY37* | MsG0480018500.01.T01 |  |  |  |  |  |  |  |  |  |  |  | 1 |  |  |  |  |  |  |  |  |  |
| *MsWRKY38* | MsG0480020316.01.T01 |  |  |  |  |  |  |  |  |  |  |  |  | 1 | 1 |  |  |  | 1 |  |  |  |
| *MsWRKY39* | MsG0480021643.01.T01 |  |  |  |  |  |  |  |  |  |  |  | 2 |  | 1 |  | 1 | 1 | 1 |  | 1 |  |
| *MsWRKY40* | MsG0480022099.01.T01 |  |  |  |  |  |  |  |  |  |  |  | 3 |  | 1 |  | 2 |  | 1 |  |  |  |
| *MsWRKY41* | MsG0480022120.01.T01 | 1 |  |  |  |  |  |  |  |  |  |  | 1 |  |  |  |  |  |  |  | 1 |  |
| *MsWRKY42* | MsG0480022721.01.T01 | 1 |  |  |  | 1 |  |  |  |  |  |  | 2 | 1 | 1 |  |  | 1 | 1 |  |  |  |
| *MsWRKY43* | MsG0480022760.01.T01 | 1 |  |  |  |  |  |  |  |  |  |  | 8 |  | 2 |  |  |  | 2 |  | 1 | 1 |
| *MsWRKY44* | MsG0480023102.01.T01 | 1 | 1 |  |  |  |  |  |  |  |  |  | 2 |  | 1 |  |  | 3 | 1 |  |  |  |
| *MsWRKY45* | MsG0480023383.01.T01 |  |  |  |  |  |  |  |  |  |  |  | 5 |  | 2 |  |  |  | 2 |  |  |  |
| *MsWRKY46* | MsG0480023394.01.T01 | 1 |  |  |  |  |  |  |  |  |  |  |  |  | 1 |  | 2 |  | 1 |  | 2 |  |
| *MsWRKY47* | MsG0480023599.01.T01 |  |  |  |  |  |  |  |  |  |  | 1 | 4 | 1 | 1 | 2 | 1 |  | 1 |  | 1 |  |
| *MsWRKY48* | MsG0480023675.01.T01 |  |  |  |  |  |  |  |  |  |  |  | 1 |  |  |  |  |  |  |  | 1 |  |
| *MsWRKY49* | MsG0580024796.01.T01 |  |  |  |  |  |  |  |  |  |  |  | 2 |  |  |  | 3 |  |  |  | 3 |  |
| *MsWRKY50* | MsG0580026252.01.T01 | 1 |  |  |  |  |  |  |  |  |  |  | 1 |  | 1 | 1 |  | 1 | 1 |  | 1 |  |
| *MsWRKY51* | MsG0580026322.01.T01 |  |  |  |  |  |  |  |  |  |  |  | 2 | 1 | 1 |  | 1 | 2 | 1 | 1 |  |  |
| *MsWRKY52* | MsG0580028140.01.T01 |  |  |  |  |  |  |  |  |  | 1 |  |  |  |  |  | 1 |  |  |  |  |  |
| *MsWRKY53* | MsG0580028530.01.T01 |  |  |  |  |  |  |  |  |  |  |  | 1 |  |  | 2 | 1 |  |  |  |  | 1 |
| *MsWRKY54* | MsG0580028539.01.T01 |  | 1 |  |  |  |  |  |  |  |  |  |  |  |  |  |  |  |  |  |  |  |
| *MsWRKY55* | MsG0580028541.01.T01 |  |  |  |  |  | 1 | 1 |  |  |  |  |  |  |  |  | 1 | 1 |  |  |  |  |
| *MsWRKY56* | MsG0580028547.01.T01 |  |  |  |  |  |  | 1 |  |  |  |  |  |  |  |  | 1 | 1 |  |  |  |  |
| *MsWRKY57* | MsG0580028553.01.T02 |  |  |  |  |  |  |  |  |  |  |  | 1 |  | 1 | 1 | 1 |  | 1 | 1 | 1 |  |
| *MsWRKY58* | MsG0580028560.01.T01 |  |  |  |  |  |  |  |  |  |  |  |  |  | 1 |  | 1 |  | 1 |  |  |  |
| *MsWRKY59* | MsG0580028889.01.T01 | 1 |  |  |  |  |  |  |  |  |  |  | 2 |  |  |  |  |  |  |  |  |  |
| *MsWRKY60* | MsG0580029904.01.T01 |  | 1 |  |  |  |  |  | 1 |  |  |  |  |  | 1 |  | 1 |  | 1 |  |  |  |
| *MsWRKY61* | MsG0680031870.01.T01 |  |  |  |  |  |  |  |  |  |  |  | 1 |  |  |  |  |  |  |  | 1 |  |
| *MsWRKY62* | MsG0680032825.01.T01 |  |  |  |  |  |  |  |  |  |  |  | 1 |  |  |  | 1 | 1 |  | 1 |  |  |
| *MsWRKY63* | MsG0680032826.01.T01 |  | 1 |  |  |  |  |  |  |  |  |  |  | 1 | 2 |  | 1 |  | 2 | 1 |  |  |
| *MsWRKY64* | MsG0680033446.01.T01 |  |  |  |  |  |  |  |  |  |  |  | 1 |  |  |  |  |  |  |  | 1 |  |
| *MsWRKY65* | MsG0680035649.01.T01 |  | 1 |  |  |  |  |  |  |  |  |  | 2 |  | 6 |  | 1 |  | 6 |  | 1 |  |
| *MsWRKY66* | MsG0780036199.01.T01 |  |  |  |  |  |  |  |  |  |  |  | 1 | 2 | 1 |  | 2 | 1 | 1 |  |  |  |
| *MsWRKY67* | MsG0780036361.01.T01 |  |  |  |  |  |  |  |  |  |  |  | 1 |  |  |  |  | 1 |  |  |  |  |
| *MsWRKY68* | MsG0780037590.01.T01 | 1 |  |  |  |  |  |  |  |  |  |  | 5 |  | 2 |  |  |  |  | 1 |  |  |
| *MsWRKY69* | MsG0780037760.01.T01 |  |  |  |  |  |  |  |  |  |  |  |  |  |  |  |  |  | 2 |  |  |  |
| *MsWRKY70* | MsG0780038789.01.T01 | 1 |  |  |  |  |  |  |  |  |  |  |  |  |  |  | 1 | 1 |  |  | 1 |  |
| *MsWRKY71* | MsG0780039334.01.T01 |  |  |  |  |  |  |  |  |  |  | 1 |  |  |  |  | 4 |  |  |  |  |  |
| *MsWRKY72* | MsG0780039432.01.T01 |  |  |  |  |  |  |  |  |  |  |  | 2 |  | 3 | 1 | 1 | 1 | 3 |  | 1 |  |
| *MsWRKY73* | MsG0780039433.01.T01 | 2 |  |  |  |  |  |  |  |  |  |  | 1 |  | 1 |  | 1 | 3 | 1 |  |  |  |
| *MsWRKY74* | MsG0780039770.01.T01 |  |  |  |  |  |  |  | 1 |  |  |  | 3 | 1 | 1 |  | 1 |  | 1 | 1 | 1 |  |
| *MsWRKY75* | MsG0780039773.01.T03 |  |  | 1 |  |  |  |  |  |  |  |  |  | 1 | 2 | 1 |  |  | 2 |  |  | 2 |
| *MsWRKY76* | MsG0780039824.01.T01 |  | 1 |  |  |  |  |  |  | 1 |  |  | 1 | 2 | 1 |  |  |  | 1 |  | 1 |  |
| *MsWRKY77* | MsG0780039825.01.T01 |  |  |  |  |  |  |  |  |  |  |  | 7 |  | 2 |  |  |  | 2 |  | 1 |  |
| *MsWRKY78* | MsG0780040890.01.T01 |  |  |  |  |  |  |  |  |  |  |  |  |  | 1 | 1 |  |  |  | 1 |  |  |
| *MsWRKY79* | MsG0780041366.01.T01 | 1 |  |  |  |  |  |  |  |  |  |  | 7 |  | 1 | 1 |  |  | 1 |  |  |  |
| *MsWRKY80* | MsG0780041380.01.T01 |  |  |  |  |  |  |  |  |  |  |  | 1 |  | 2 |  | 3 | 1 | 2 |  |  |  |
| *MsWRKY81* | MsG0780041425.01.T01 |  |  |  | 1 |  |  |  |  |  |  |  | 3 |  | 3 | 2 |  |  | 3 |  |  |  |
| *MsWRKY82* | MsG0780041742.01.T01 |  |  |  |  |  |  |  |  |  |  |  | 3 |  | 2 |  |  |  | 2 |  |  |  |
| *MsWRKY83* | MsG0880041942.01.T01 |  |  |  |  |  |  |  |  |  |  |  | 3 |  |  |  |  | 1 |  | 1 |  |  |
| *MsWRKY84* | MsG0880043109.01.T01 |  |  |  |  |  |  |  |  |  |  |  | 1 |  |  |  | 1 |  |  |  |  |  |
| *MsWRKY85* | MsG0880045340.01.T01 |  |  |  |  |  |  |  |  |  |  |  | 1 |  | 1 |  |  | 1 | 6 | 1 |  |  |
| *MsWRKY86* | MsG0880045907.01.T01 |  |  |  | 1 |  |  |  |  |  |  |  |  |  | 1 |  | 2 |  | 1 |  |  |  |
| *MsWRKY87* | MsG0880046429.01.T01 |  |  |  | 1 |  |  |  |  |  |  |  |  |  | 2 |  | 1 | 2 |  |  |  |  |
| *MsWRKY88* | MsG0880047174.01.T01 | 1 |  |  |  |  |  |  |  |  |  |  | 2 | 1 |  |  |  | 1 |  | 1 |  |  |
| *MsWRKY89* | MsG0880047271.01.T01 |  |  |  |  |  |  |  |  |  |  |  | 1 |  | 2 |  |  |  | 2 |  |  |  |
| *MsWRKY90* | MsG0880047597.01.T01 |  |  |  | 1 |  |  |  |  |  |  |  | 1 |  | 2 |  |  | 1 | 3 |  |  |  |
| *MsWRKY91* | MsG0880047665.01.T01 |  | 1 |  |  |  |  |  |  |  |  |  | 3 |  | 5 |  | 1 |  | 5 |  |  |  |

| **Catergory** |  | **evironment responsive element** | | | | | | | **promoter related and bingding sites element** | | | | | |
| --- | --- | --- | --- | --- | --- | --- | --- | --- | --- | --- | --- | --- | --- | --- |
| *Cis*-acting element |  | ARE | GC-motif | LTR | MBS | TC-rich repeats | WUN-motif | LTR | HD-Zip | W-box | A-box | CCAAT-box | AT-rich element | 3-AF3  binding site |
|  |  | cis-acting regulatory element essential for the anaerobic induction | enhancer-like element involved in anoxic specific inducibility | cis-acting element involved in low-temperature responsiveness | MYB binding site involved in drought-inducibility | cis-acting element involved in defense and stress responsiveness | wound-responsive element | cis-acting element involved in low-temperature responsiveness | element involved in differentiation of the palisade mesophyll cells | WRKY transcription factor binding site in defense responses | cis-acting regulatory element | MYBHv1 binding site | binding  site of AT-rich DNA binding protein (ATBP-1) | part of a  conserved DNA module array (CMA3) |
| *MsWRKY1* | MsG0080048036.01.T01 |  |  |  |  |  |  |  |  |  |  | 1 |  |  |
| *MsWRKY2* | MsG0080048967.01.T01 |  |  | 1 | 1 | 1 | 1 |  |  |  |  |  | 2 |  |
| *MsWRKY3* | MsG0080049021.01.T01 | 6 |  |  | 2 |  |  |  |  |  |  |  | 1 |  |
| *MsWRKY4* | MsG0180000474.01.T01 | 1 |  |  | 1 |  |  | 1 |  |  |  |  | 1 |  |
| *MsWRKY5* | MsG0180000525.01.T01 | 3 |  |  | 1 |  |  |  |  |  |  |  |  |  |
| *MsWRKY6* | MsG0180000526.01.T01 |  |  |  | 1 |  |  |  |  |  |  | 1 |  | 1 |
| *MsWRKY7* | MsG0180000738.01.T01 |  |  |  | 1 |  |  |  |  |  |  |  |  |  |
| *MsWRKY8* | MsG0180003898.01.T01 |  | 1 |  |  | 1 |  |  |  |  |  |  | 1 |  |
| *MsWRKY9* | MsG0180004240.01.T01 | 3 | 1 |  |  | 1 |  |  |  |  |  |  | 1 | 2 |
| *MsWRKY10* | MsG0180004365.01.T01 | 1 |  |  |  | 1 |  |  |  |  |  |  |  | 1 |
| *MsWRKY11* | MsG0180004777.01.T01 | 2 |  | 2 | 1 | 1 |  |  | 1 |  |  |  |  | 1 |
| *MsWRKY12* | MsG0280006932.01.T01 |  |  | 3 | 1 |  |  |  |  |  |  |  |  | 1 |
| *MsWRKY13* | MsG0280007272.01.T01 | 1 | 1 |  |  | 1 |  |  | 1 |  |  |  |  |  |
| *MsWRKY14* | MsG0280007369.01.T01 | 3 |  |  |  | 1 |  |  | 1 |  |  | 1 |  |  |
| *MsWRKY15* | MsG0280007391.01.T01 | 4 | 1 |  |  | 1 |  |  | 1 |  |  |  |  | 1 |
| *MsWRKY16* | MsG0280007412.01.T01 | 6 | 1 |  | 2 |  |  |  |  |  |  | 1 |  |  |
| *MsWRKY17* | MsG0280007413.01.T01 | 4 |  |  | 2 |  |  |  |  |  |  | 1 |  |  |
| *MsWRKY18* | MsG0280007786.01.T01 | 4 |  |  | 6 |  |  |  |  |  |  |  |  |  |
| *MsWRKY19* | MsG0280007840.01.T01 | 6 |  | 1 | 1 |  |  |  |  |  |  |  | 2 |  |
| *MsWRKY20* | MsG0280008601.01.T01 |  |  | 1 | 1 |  |  |  |  |  |  |  |  |  |
| *MsWRKY21* | MsG0280009986.01.T01 | 1 |  |  | 1 |  |  |  |  |  |  |  |  |  |
| *MsWRKY22* | MsG0280009987.01.T01 | 1 |  |  | 1 |  |  |  |  |  |  |  |  |  |
| *MsWRKY23* | MsG0280009989.01.T01 |  |  |  | 2 |  |  |  |  |  |  |  |  |  |
| *MsWRKY24* | MsG0280009990.01.T01 | 1 |  |  | 2 |  |  |  | 1 |  |  | 2 | 1 |  |
| *MsWRKY25* | MsG0280010417.01.T01 |  |  | 1 |  |  |  |  |  |  |  |  |  |  |
| *MsWRKY26* | MsG0280010588.01.T01 | 2 |  |  |  |  |  |  |  |  |  |  |  |  |
| *MsWRKY27* | MsG0280011473.01.T01 | 2 |  | 2 | 1 | 1 |  |  |  |  |  | 1 |  |  |
| *MsWRKY28* | MsG0380014238.01.T01 | 1 |  |  |  | 1 |  |  |  |  |  |  | 1 |  |
| *MsWRKY29* | MsG0380014401.01.T01 | 3 |  | 1 | 1 |  |  |  |  |  |  | 1 |  |  |
| *MsWRKY30* | MsG0380014920.01.T01 | 4 |  | 2 | 1 |  |  |  |  |  |  | 1 | 1 |  |
| *MsWRKY31* | MsG0380016708.01.T01 |  |  |  | 1 |  |  |  | 1 |  |  |  |  |  |
| *MsWRKY32* | MsG0380016765.01.T01 | 2 |  |  | 1 | 1 |  |  |  |  |  |  |  |  |
| *MsWRKY33* | MsG0380017296.01.T01 | 1 | 1 |  |  | 2 |  |  |  |  |  | 1 | 1 |  |
| *MsWRKY34* | MsG0380017368.01.T02 | 3 |  |  |  |  |  |  |  |  |  |  | 1 |  |
| *MsWRKY35* | MsG0380017553.01.T01 | 1 | 1 |  |  |  |  |  |  |  |  |  | 1 |  |
| *MsWRKY36* | MsG0480018188.01.T01 | 3 |  |  |  |  |  |  |  |  |  |  | 1 |  |
| *MsWRKY37* | MsG0480018500.01.T01 | 3 |  |  | 1 |  |  |  |  |  |  | 1 | 1 |  |
| *MsWRKY38* | MsG0480020316.01.T01 | 2 |  | 1 |  | 2 |  |  |  |  |  | 1 |  |  |
| *MsWRKY39* | MsG0480021643.01.T01 | 4 |  |  |  | 1 |  |  |  |  |  | 1 | 1 |  |
| *MsWRKY40* | MsG0480022099.01.T01 | 4 |  |  | 1 | 1 |  |  |  |  |  |  |  |  |
| *MsWRKY41* | MsG0480022120.01.T01 | 1 |  |  |  |  |  |  |  |  |  |  |  |  |
| *MsWRKY42* | MsG0480022721.01.T01 |  |  |  | 1 | 1 |  |  |  |  | 1 |  | 2 |  |
| *MsWRKY43* | MsG0480022760.01.T01 | 4 |  | 1 | 1 |  |  |  |  |  |  |  |  |  |
| *MsWRKY44* | MsG0480023102.01.T01 | 6 |  |  |  |  |  |  |  |  |  |  |  |  |
| *MsWRKY45* | MsG0480023383.01.T01 | 1 |  | 1 |  |  |  |  |  |  |  |  | 1 |  |
| *MsWRKY46* | MsG0480023394.01.T01 |  |  |  | 2 |  |  |  |  |  |  | 1 |  |  |
| *MsWRKY47* | MsG0480023599.01.T01 |  |  | 1 | 3 |  |  |  |  |  |  | 1 |  |  |
| *MsWRKY48* | MsG0480023675.01.T01 | 1 |  | 1 |  |  |  |  |  |  |  |  | 1 |  |
| *MsWRKY49* | MsG0580024796.01.T01 | 1 |  | 2 | 1 |  |  |  |  |  |  |  | 1 |  |
| *MsWRKY50* | MsG0580026252.01.T01 |  |  | 1 | 2 |  |  |  |  |  |  | 1 |  |  |
| *MsWRKY51* | MsG0580026322.01.T01 | 2 |  |  | 1 |  | 1 |  |  |  |  |  |  |  |
| *MsWRKY52* | MsG0580028140.01.T01 |  |  |  | 1 |  |  |  | 2 |  |  |  | 2 |  |
| *MsWRKY53* | MsG0580028530.01.T01 | 4 |  | 2 |  | 1 |  |  | 1 |  |  |  |  |  |
| *MsWRKY54* | MsG0580028539.01.T01 | 1 |  | 2 | 1 | 1 |  |  |  |  |  |  | 1 |  |
| *MsWRKY55* | MsG0580028541.01.T01 | 2 |  |  | 1 |  |  |  |  |  |  |  |  |  |
| *MsWRKY56* | MsG0580028547.01.T01 | 2 |  |  | 1 |  | 1 |  |  |  |  |  |  |  |
| *MsWRKY57* | MsG0580028553.01.T02 | 3 |  | 1 | 2 |  |  |  |  |  |  |  | 1 |  |
| *MsWRKY58* | MsG0580028560.01.T01 | 3 |  |  | 2 |  |  |  |  |  |  |  | 1 |  |
| *MsWRKY59* | MsG0580028889.01.T01 |  |  |  | 3 |  |  |  |  |  |  |  |  |  |
| *MsWRKY60* | MsG0580029904.01.T01 | 2 |  |  |  | 1 |  |  |  |  |  |  |  |  |
| *MsWRKY61* | MsG0680031870.01.T01 | 3 |  | 1 | 2 |  |  |  |  |  |  | 1 | 2 |  |
| *MsWRKY62* | MsG0680032825.01.T01 | 3 |  |  |  |  |  |  |  |  |  |  |  |  |
| *MsWRKY63* | MsG0680032826.01.T01 | 3 | 1 |  | 5 |  |  |  |  |  |  | 3 |  |  |
| *MsWRKY64* | MsG0680033446.01.T01 | 1 |  |  |  |  |  |  |  |  |  |  | 1 |  |
| *MsWRKY65* | MsG0680035649.01.T01 |  |  |  | 1 |  | 1 |  |  |  |  |  |  |  |
| *MsWRKY66* | MsG0780036199.01.T01 | 2 |  |  | 4 | 1 |  |  |  |  |  |  | 1 |  |
| *MsWRKY67* | MsG0780036361.01.T01 | 1 |  |  |  |  |  |  |  |  |  |  |  |  |
| *MsWRKY68* | MsG0780037590.01.T01 | 2 |  | 1 |  |  |  |  |  |  |  | 1 |  |  |
| *MsWRKY69* | MsG0780037760.01.T01 | 2 |  | 1 | 1 |  |  |  |  |  |  |  |  |  |
| *MsWRKY70* | MsG0780038789.01.T01 | 2 |  |  | 1 | 1 |  |  | 1 |  |  |  | 1 |  |
| *MsWRKY71* | MsG0780039334.01.T01 | 3 |  |  |  |  |  |  |  |  |  |  |  |  |
| *MsWRKY72* | MsG0780039432.01.T01 |  | 1 |  | 2 |  |  |  |  |  |  |  | 1 |  |
| *MsWRKY73* | MsG0780039433.01.T01 | 3 |  |  | 1 | 2 |  |  |  |  |  |  | 2 |  |
| *MsWRKY74* | MsG0780039770.01.T01 |  |  |  | 1 |  |  |  |  |  |  |  |  |  |
| *MsWRKY75* | MsG0780039773.01.T03 |  |  |  | 3 |  |  |  |  |  |  |  |  |  |
| *MsWRKY76* | MsG0780039824.01.T01 |  |  | 1 |  | 2 |  |  |  |  |  | 1 | 1 |  |
| *MsWRKY77* | MsG0780039825.01.T01 | 5 |  | 1 |  |  | 1 |  | 1 |  |  |  | 1 |  |
| *MsWRKY78* | MsG0780040890.01.T01 |  |  |  | 3 |  |  |  |  |  |  |  |  |  |
| *MsWRKY79* | MsG0780041366.01.T01 |  |  | 1 | 1 |  |  |  |  |  |  |  |  |  |
| *MsWRKY80* | MsG0780041380.01.T01 | 1 | 1 |  |  |  |  |  |  |  |  |  | 1 |  |
| *MsWRKY81* | MsG0780041425.01.T01 |  |  |  | 4 | 1 |  |  | 1 |  |  |  |  |  |
| *MsWRKY82* | MsG0780041742.01.T01 | 3 |  |  |  |  |  |  |  |  |  |  |  |  |
| *MsWRKY83* | MsG0880041942.01.T01 | 4 |  |  |  |  |  |  |  |  |  |  |  |  |
| *MsWRKY84* | MsG0880043109.01.T01 |  |  | 1 |  |  |  |  |  |  |  |  |  |  |
| *MsWRKY85* | MsG0880045340.01.T01 | 2 |  |  |  |  |  |  |  |  |  |  |  |  |
| *MsWRKY86* | MsG0880045907.01.T01 |  |  |  |  | 1 |  |  | 1 |  |  |  |  |  |
| *MsWRKY87* | MsG0880046429.01.T01 | 1 |  |  | 1 |  |  |  |  |  |  | 1 |  |  |
| *MsWRKY88* | MsG0880047174.01.T01 |  |  |  |  | 1 |  |  |  |  |  | 1 |  |  |
| *MsWRKY89* | MsG0880047271.01.T01 | 1 |  |  |  |  |  |  |  |  |  |  |  |  |
| *MsWRKY90* | MsG0880047597.01.T01 | 3 |  |  |  | 1 |  |  |  |  |  |  |  |  |
| *MsWRKY91* | MsG0880047665.01.T01 | 2 |  |  | 1 | 1 |  |  |  |  |  |  |  |  |

| Catergory |  | development related element | | | | | | | | | | unknown |
| --- | --- | --- | --- | --- | --- | --- | --- | --- | --- | --- | --- | --- |
| *Cis*-acting element |  | CAT-box | o2-site | GCN4_motif | RY-element | GCN4_motif | MSA-like | circadian | AACA_motif | Box III | 5UTR Py-rich stretch | CCGTCC-box |
|  |  | cis-acting regulatory element related to meristem expression | cis-acting regulatory element involved in zein metabolism regulation | cis-regulatory element involved in endosperm expression | cis-acting  regulatory element involved in seed-specific regulation | cis-regulatory element involved in endosperm expression | cis-acting element involved in cell cycle regulation | cis-acting  regulatory element involved in circadian control | involved in endosperm-specific negative expression | protein binding site | cis-acting element conferring high transcription levels | unknown |
| *MsWRKY1* | MsG0080048036.01.T01 |  |  |  |  |  |  |  |  |  |  | 1 |
| *MsWRKY2* | MsG0080048967.01.T01 |  |  |  |  |  |  |  |  |  |  |  |
| *MsWRKY3* | MsG0080049021.01.T01 |  |  |  |  |  |  |  |  |  |  |  |
| *MsWRKY4* | MsG0180000474.01.T01 |  | 2 |  |  |  |  |  |  |  |  |  |
| *MsWRKY5* | MsG0180000525.01.T01 | 1 |  |  |  |  |  |  |  |  |  |  |
| *MsWRKY6* | MsG0180000526.01.T01 |  |  |  |  |  |  |  |  |  |  |  |
| *MsWRKY7* | MsG0180000738.01.T01 |  |  | 1 |  |  |  |  |  |  |  |  |
| *MsWRKY8* | MsG0180003898.01.T01 |  | 1 |  | 1 |  |  | 1 |  |  |  |  |
| *MsWRKY9* | MsG0180004240.01.T01 |  |  |  |  |  |  |  |  |  |  |  |
| *MsWRKY10* | MsG0180004365.01.T01 |  | 1 |  |  |  |  |  |  |  |  |  |
| *MsWRKY11* | MsG0180004777.01.T01 | 1 |  |  |  |  |  |  |  |  |  |  |
| *MsWRKY12* | MsG0280006932.01.T01 | 2 |  |  |  |  |  |  |  |  |  |  |
| *MsWRKY13* | MsG0280007272.01.T01 |  | 1 |  |  |  |  | 1 |  |  |  |  |
| *MsWRKY14* | MsG0280007369.01.T01 |  |  |  |  |  |  |  |  |  |  |  |
| *MsWRKY15* | MsG0280007391.01.T01 |  | 1 | 1 |  |  |  | 1 |  |  |  |  |
| *MsWRKY16* | MsG0280007412.01.T01 | 1 | 1 |  |  |  |  |  |  |  |  |  |
| *MsWRKY17* | MsG0280007413.01.T01 |  |  |  |  |  |  |  |  |  |  |  |
| *MsWRKY18* | MsG0280007786.01.T01 |  |  |  |  |  |  |  |  |  |  |  |
| *MsWRKY19* | MsG0280007840.01.T01 |  | 2 |  |  |  |  | 1 |  |  |  |  |
| *MsWRKY20* | MsG0280008601.01.T01 | 1 |  |  |  |  |  |  |  |  |  |  |
| *MsWRKY21* | MsG0280009986.01.T01 |  |  |  |  | 1 |  |  |  |  |  |  |
| *MsWRKY22* | MsG0280009987.01.T01 |  |  | 1 |  |  |  |  |  |  |  |  |
| *MsWRKY23* | MsG0280009989.01.T01 | 1 |  |  |  |  | 1 |  |  |  |  |  |
| *MsWRKY24* | MsG0280009990.01.T01 | 1 |  |  |  |  |  |  |  |  |  |  |
| *MsWRKY25* | MsG0280010417.01.T01 |  |  |  |  |  |  |  |  |  |  |  |
| *MsWRKY26* | MsG0280010588.01.T01 | 1 | 1 |  |  |  |  |  | 1 |  |  |  |
| *MsWRKY27* | MsG0280011473.01.T01 |  |  |  |  |  |  |  |  |  |  |  |
| *MsWRKY28* | MsG0380014238.01.T01 |  |  | 1 |  |  |  |  |  |  |  |  |
| *MsWRKY29* | MsG0380014401.01.T01 | 1 |  | 1 | 1 |  |  |  |  | 1 |  |  |
| *MsWRKY30* | MsG0380014920.01.T01 |  | 1 |  |  |  | 1 |  |  |  |  |  |
| *MsWRKY31* | MsG0380016708.01.T01 |  | 1 |  |  |  |  |  |  |  |  |  |
| *MsWRKY32* | MsG0380016765.01.T01 |  |  |  |  |  |  |  |  |  |  |  |
| *MsWRKY33* | MsG0380017296.01.T01 | 1 |  |  | 1 |  |  |  |  |  |  |  |
| *MsWRKY34* | MsG0380017368.01.T02 |  |  |  |  |  |  |  |  |  |  |  |
| *MsWRKY35* | MsG0380017553.01.T01 |  |  |  |  |  |  |  |  |  |  |  |
| *MsWRKY36* | MsG0480018188.01.T01 | 1 |  |  |  |  |  |  |  |  |  |  |
| *MsWRKY37* | MsG0480018500.01.T01 |  | 1 |  | 1 |  |  | 3 |  | 1 |  |  |
| *MsWRKY38* | MsG0480020316.01.T01 |  |  | 2 |  |  |  |  |  |  |  |  |
| *MsWRKY39* | MsG0480021643.01.T01 |  |  |  |  |  |  |  |  |  |  |  |
| *MsWRKY40* | MsG0480022099.01.T01 | 1 | 2 |  |  |  |  |  |  |  |  |  |
| *MsWRKY41* | MsG0480022120.01.T01 |  | 1 |  |  |  |  |  |  |  | 1 |  |
| *MsWRKY42* | MsG0480022721.01.T01 |  | 1 |  | 1 |  |  |  |  |  |  |  |
| *MsWRKY43* | MsG0480022760.01.T01 |  | 1 |  |  |  |  |  |  |  |  |  |
| *MsWRKY44* | MsG0480023102.01.T01 |  |  | 1 |  |  |  |  |  |  |  |  |
| *MsWRKY45* | MsG0480023383.01.T01 | 1 |  | 1 |  |  |  |  |  |  |  |  |
| *MsWRKY46* | MsG0480023394.01.T01 |  |  |  | 1 |  |  |  |  |  |  |  |
| *MsWRKY47* | MsG0480023599.01.T01 | 2 |  | 1 |  |  |  |  |  |  |  |  |
| *MsWRKY48* | MsG0480023675.01.T01 |  |  |  |  |  |  | 1 |  |  |  |  |
| *MsWRKY49* | MsG0580024796.01.T01 |  | 1 |  |  |  |  | 1 |  |  |  |  |
| *MsWRKY50* | MsG0580026252.01.T01 |  |  |  |  |  |  | 1 |  |  |  |  |
| *MsWRKY51* | MsG0580026322.01.T01 | 2 | 1 | 1 |  |  |  |  |  |  |  |  |
| *MsWRKY52* | MsG0580028140.01.T01 | 1 |  |  |  |  |  |  |  |  |  |  |
| *MsWRKY53* | MsG0580028530.01.T01 | 2 |  |  |  |  |  |  |  |  |  |  |
| *MsWRKY54* | MsG0580028539.01.T01 |  |  |  |  |  |  |  |  |  |  |  |
| *MsWRKY55* | MsG0580028541.01.T01 | 1 | 2 |  |  |  |  |  |  |  |  |  |
| *MsWRKY56* | MsG0580028547.01.T01 | 1 | 1 |  |  |  |  |  |  |  |  |  |
| *MsWRKY57* | MsG0580028553.01.T02 |  | 2 |  |  |  |  |  |  |  |  |  |
| *MsWRKY58* | MsG0580028560.01.T01 |  |  |  |  |  |  |  |  |  |  |  |
| *MsWRKY59* | MsG0580028889.01.T01 |  |  |  |  |  |  |  |  |  |  |  |
| *MsWRKY60* | MsG0580029904.01.T01 |  | 1 | 1 |  |  |  | 1 |  |  |  |  |
| *MsWRKY61* | MsG0680031870.01.T01 | 1 |  |  |  |  |  |  | 1 |  |  |  |
| *MsWRKY62* | MsG0680032825.01.T01 |  | 1 |  |  |  |  |  |  |  |  |  |
| *MsWRKY63* | MsG0680032826.01.T01 | 2 | 1 |  |  |  |  |  |  |  |  |  |
| *MsWRKY64* | MsG0680033446.01.T01 | 1 | 1 |  |  |  |  |  |  |  |  |  |
| *MsWRKY65* | MsG0680035649.01.T01 |  |  | 2 |  |  |  |  |  |  |  |  |
| *MsWRKY66* | MsG0780036199.01.T01 |  |  |  |  |  |  |  |  |  |  |  |
| *MsWRKY67* | MsG0780036361.01.T01 |  | 1 |  |  |  |  |  |  |  |  |  |
| *MsWRKY68* | MsG0780037590.01.T01 |  |  | 1 |  |  |  |  |  |  |  |  |
| *MsWRKY69* | MsG0780037760.01.T01 |  |  |  |  |  |  |  |  |  |  |  |
| *MsWRKY70* | MsG0780038789.01.T01 |  |  |  |  |  |  |  |  |  |  |  |
| *MsWRKY71* | MsG0780039334.01.T01 |  | 1 |  |  |  |  |  |  | 1 |  |  |
| *MsWRKY72* | MsG0780039432.01.T01 | 1 | 1 |  |  |  |  | 1 |  |  |  |  |
| *MsWRKY73* | MsG0780039433.01.T01 | 1 |  | 1 |  |  |  | 1 |  |  |  |  |
| *MsWRKY74* | MsG0780039770.01.T01 |  | 2 |  |  |  |  |  |  |  |  |  |
| *MsWRKY75* | MsG0780039773.01.T03 |  |  |  |  |  |  |  |  |  |  |  |
| *MsWRKY76* | MsG0780039824.01.T01 |  |  |  |  |  | 2 |  |  |  |  |  |
| *MsWRKY77* | MsG0780039825.01.T01 |  | 1 |  |  |  |  |  |  |  |  |  |
| *MsWRKY78* | MsG0780040890.01.T01 | 1 |  |  |  |  |  |  |  |  |  |  |
| *MsWRKY79* | MsG0780041366.01.T01 | 1 | 1 | 1 |  |  |  |  |  |  |  |  |
| *MsWRKY80* | MsG0780041380.01.T01 | 1 |  |  |  |  |  |  |  |  |  |  |
| *MsWRKY81* | MsG0780041425.01.T01 |  | 2 |  |  |  |  | 2 |  |  |  |  |
| *MsWRKY82* | MsG0780041742.01.T01 |  |  |  |  |  |  | 1 |  |  |  |  |
| *MsWRKY83* | MsG0880041942.01.T01 |  | 2 |  | 1 |  |  | 1 |  |  |  |  |
| *MsWRKY84* | MsG0880043109.01.T01 |  | 2 | 2 |  |  |  |  |  |  |  |  |
| *MsWRKY85* | MsG0880045340.01.T01 |  |  |  |  |  |  |  |  |  |  |  |
| *MsWRKY86* | MsG0880045907.01.T01 |  |  |  |  |  |  |  |  |  |  |  |
| *MsWRKY87* | MsG0880046429.01.T01 |  |  |  |  |  |  |  |  |  |  |  |
| *MsWRKY88* | MsG0880047174.01.T01 |  |  |  | 1 |  |  |  |  |  |  |  |
| *MsWRKY89* | MsG0880047271.01.T01 |  |  |  |  |  |  | 1 |  |  |  |  |
| *MsWRKY90* | MsG0880047597.01.T01 |  |  |  |  |  |  |  |  |  |  |  |
| *MsWRKY91* | MsG0880047665.01.T01 |  |  |  |  |  |  |  |  | 1 |  |  |
