# Supplementary material for: Genomic identification and expression profiling of WRKY genes in alfalfa (Medicago sativa) elucidate their responsiveness to seed vigor
Source: BMC Plant Biol. 2023 Nov 16;23:568. doi: 10.1186/s12870-023-04597-x (PMC10652462; doi:10.1186/s12870-023-04597-x)
Supplement: Supplementary file 7 — Additional file 7: Table S7. The secondary structure of MsWRKY proteins. [file 12870_2023_4597_MOESM7_ESM.docx]

| **Table S7: The secondary structure of MsWRKY proteins** | | | | |
| --- | --- | --- | --- | --- |
| Gene name | α-helix(%) | Extended strand(%) | β-turn(%) | Random coil(%) |
| MsWRKY01 | 22.89% | 10.39% | 2.44% | 64.29% |
| MsWRKY02 | 31.02% | 8.03% | 5.84% | 55.11% |
| MsWRKY03 | 18.23% | 10.79% | 2.88% | 68.11% |
| MsWRKY04 | 24.55% | 14.97% | 7.78% | 52.69% |
| MsWRKY05 | 31.48% | 13.44% | 1.97% | 53.11% |
| MsWRKY06 | 31.87% | 16.33% | 3.59% | 48.21% |
| MsWRKY07 | 18.24% | 11.18% | 6.76% | 63.82% |
| MsWRKY08 | 25.28% | 9.44% | 4.17% | 61.11% |
| MsWRKY09 | 15.49% | 19.72% | 5.63% | 59.15% |
| MsWRKY10 | 30.45% | 24.22% | 11.76% | 33.56% |
| MsWRKY11 | 25.08% | 7.03% | 1.53% | 66.36% |
| MsWRKY12 | 21.90% | 15.24% | 5.24% | 57.62% |
| MsWRKY13 | 32.00% | 12.00% | 2.46% | 53.54% |
| MsWRKY14 | 32.99% | 12.18% | 4.57% | 50.25% |
| MsWRKY15 | 26.47% | 12.79% | 3.02% | 57.73% |
| MsWRKY16 | 19.37% | 25.68% | 3.15% | 51.80% |
| MsWRKY17 | 14.05% | 19.91% | 5.15% | 60.89% |
| MsWRKY18 | 19.12% | 9.66% | 3.99% | 67.23% |
| MsWRKY19 | 20.93% | 18.27% | 5.98% | 54.82% |
| MsWRKY20 | 38.31% | 11.29% | 2.42% | 47.98% |
| MsWRKY21 | 15.53% | 20.55% | 5.02% | 58.90% |
| MsWRKY22 | 28.20% | 15.41% | 1.64% | 54.75% |
| MsWRKY23 | 18.18% | 18.18% | 4.85% | 58.79% |
| MsWRKY24 | 36.96% | 18.48% | 1.81% | 42.75% |
| MsWRKY25 | 12.26% | 12.26% | 4.60% | 70.88% |
| MsWRKY26 | 24.76% | 10.14% | 2.59% | 62.50% |
| MsWRKY27 | 25.87% | 11.36% | 2.84% | 59.94% |
| MsWRKY28 | 34.96% | 19.59% | 7.90% | 37.55% |
| MsWRKY29 | 10.20% | 11.43% | 5.31% | 73.06% |
| MsWRKY30 | 12.33% | 10.37% | 2.94% | 74.36% |
| MsWRKY31 | 26.54% | 12.96% | 3.09% | 57.41% |
| MsWRKY32 | 24.69% | 13.99% | 2.88% | 58.44% |
| MsWRKY33 | 22.50% | 16.67% | 4.17% | 56.67% |
| MsWRKY34 | 20.81% | 20.81% | 6.04% | 52.35% |
| MsWRKY35 | 13.08% | 20.00% | 8.46% | 58.46% |
| MsWRKY36 | 29.30% | 10.19% | 2.23% | 58.28% |
| MsWRKY37 | 18.66% | 14.35% | 5.74% | 61.24% |
| MsWRKY38 | 26.91% | 18.52% | 4.44% | 50.12% |
| MsWRKY39 | 7.98% | 14.40% | 2.97% | 74.65% |
| MsWRKY40 | 21.77% | 15.87% | 4.92% | 57.44% |
| MsWRKY41 | 22.61% | 11.30% | 4.41% | 61.69% |
| MsWRKY42 | 33.26% | 9.89% | 2.47% | 54.38% |
| MsWRKY43 | 22.64% | 12.61% | 7.16% | 57.59% |
| MsWRKY44 | 17.70% | 9.05% | 2.88% | 70.37% |
| MsWRKY45 | 17.50% | 15.83% | 4.17% | 62.50% |
| MsWRKY46 | 32.20% | 20.41% | 8.39% | 39.00% |
| MsWRKY47 | 23.21% | 10.71% | 6.25% | 59.82% |
| MsWRKY48 | 21.33% | 10.80% | 3.60% | 64.27% |
| MsWRKY49 | 24.81% | 11.85% | 3.70% | 59.63% |
| MsWRKY50 | 11.69% | 12.17% | 3.82% | 72.32% |
| MsWRKY51 | 15.77% | 13.09% | 5.37% | 65.77% |
| MsWRKY52 | 26.81% | 10.21% | 3.40% | 59.57% |
| MsWRKY53 | 54.16% | 13.29% | 4.10% | 28.45% |
| MsWRKY54 | 50.11% | 14.06% | 3.17% | 32.65% |
| MsWRKY55 | 55.23% | 11.48% | 3.69% | 29.61% |
| MsWRKY56 | 56.18% | 14.49% | 5.51% | 23.82% |
| MsWRKY57 | 47.56% | 10.67% | 3.35% | 38.41% |
| MsWRKY58 | 11.76% | 10.73% | 2.25% | 75.26% |
| MsWRKY59 | 29.50% | 21.50% | 6.00% | 43.00% |
| MsWRKY60 | 6.93% | 12.77% | 3.83% | 76.46% |
| MsWRKY61 | 38.60% | 16.49% | 5.26% | 39.65% |
| MsWRKY62 | 15.93% | 21.02% | 3.73% | 59.32% |
| MsWRKY63 | 11.72% | 23.27% | 4.33% | 60.67% |
| MsWRKY64 | 51.47% | 23.53% | 5.88% | 19.12% |
| MsWRKY65 | 14.83% | 15.09% | 3.84% | 66.24% |
| MsWRKY66 | 29.87% | 16.88% | 5.19% | 48.05% |
| MsWRKY67 | 13.36% | 12.40% | 2.86% | 71.37% |
| MsWRKY68 | 42.00% | 13.84% | 2.39% | 41.77% |
| MsWRKY69 | 31.01% | 26.44% | 10.82% | 31.73% |
| MsWRKY70 | 29.39% | 12.16% | 3.38% | 55.07% |
| MsWRKY71 | 10.40% | 8.39% | 3.10% | 78.10% |
| MsWRKY72 | 19.58% | 17.13% | 3.50% | 59.79% |
| MsWRKY73 | 23.05% | 17.02% | 3.55% | 56.38% |
| MsWRKY74 | 23.97% | 8.90% | 6.16% | 60.96% |
| MsWRKY75 | 28.09% | 15.73% | 3.37% | 52.81% |
| MsWRKY76 | 12.59% | 12.44% | 4.35% | 70.61% |
| MsWRKY77 | 15.69% | 12.20% | 3.61% | 68.49% |
| MsWRKY78 | 17.29% | 9.80% | 3.17% | 69.74% |
| MsWRKY79 | 24.31% | 12.15% | 4.17% | 59.38% |
| MsWRKY80 | 22.64% | 6.08% | 1.69% | 69.59% |
| MsWRKY81 | 24.57% | 10.71% | 2.92% | 61.80% |
| MsWRKY82 | 22.52% | 9.31% | 2.10% | 66.07% |
| MsWRKY83 | 10.92% | 19.54% | 7.47% | 62.07% |
| MsWRKY84 | 30.29% | 8.65% | 7.69% | 53.37% |
| MsWRKY85 | 11.72% | 25.45% | 2.22% | 60.61% |
| MsWRKY86 | 23.51% | 11.01% | 2.08% | 63.39% |
| MsWRKY87 | 24.51% | 9.42% | 1.95% | 64.12% |
| MsWRKY88 | 30.39% | 13.92% | 4.41% | 51.28% |
| MsWRKY89 | 40.39% | 9.77% | 3.26% | 46.58% |
| MsWRKY90 | 17.45% | 11.07% | 3.00% | 68.48% |
| MsWRKY91 | 29.94% | 11.23% | 3.53% | 55.30% |
