# Supplementary material for: Genomic identification and expression profiling of WRKY genes in alfalfa (Medicago sativa) elucidate their responsiveness to seed vigor
Source: BMC Plant Biol. 2023 Nov 16;23:568. doi: 10.1186/s12870-023-04597-x (PMC10652462; doi:10.1186/s12870-023-04597-x)
Supplement: Supplementary file 8 — Additional file 8: Table S8. Expression (FPKM) of MsWRKY genes in different tissues. [file 12870_2023_4597_MOESM8_ESM.docx]

**Table S8: Expression (FPKM) of *MsWRKY* genes in different tissues**

| **gene id** | **Root** | **nodule** | **Elonged stem** | **Pre-elonged stem** | **Leaf** | **Flower** | **Seed** | | | |
| --- | --- | --- | --- | --- | --- | --- | --- | --- | --- | --- |
|  |  |  |  |  |  |  | **6 h** | **12 h** | **24 h** | **36 h** |
| *MsWRKY1* | 53.4277 | 3.65574 | 0.084707667 | 0.313655333 | 1.136188 | 0.2292533 | 1.40881 | 0.60449 | 0.2554 | 0.72078 |
| *MsWRKY2* | 11.9248 | 3.89135 | 2.849257 | 2.219567333 | 1.6074923 | 0 | 13.3655 | 1.23832 | 0.46743 | 1.42815 |
| *MsWRKY3* | 0.17565 | 0 | 2.626843 | 2.379738333 | 2.3907673 | 17.481263 | 0.03258 | 0.04552 | 0.19572 | 3.99544 |
| *MsWRKY4* | 0 | 0 | 0 | 1.793664333 | 9.2692947 | 3.1412513 | 0 | 0 | 0 | 0 |
| *MsWRKY5* | 26.7801 | 4.08135 | 11.73600933 | 8.027825333 | 22.381288 | 14.455858 | 0.28671 | 0 | 0 | 0 |
| *MsWRKY6* | 4.34446 | 1.27849 | 10.98507 | 7.958479 | 4.2559107 | 3.3168933 | 0 | 0 | 0 | 0 |
| *MsWRKY7* | 82.4534 | 47.0556 | 21.74460667 | 23.498586 | 11.651417 | 27.935026 | 19.9128 | 10.9361 | 21.5968 | 56.8469 |
| *MsWRKY8* | 38.3215 | 18.9634 | 31.861148 | 41.59944933 | 11.193583 | 18.880057 | 12.7668 | 19.0732 | 11.5586 | 20.7865 |
| *MsWRKY9* | 0 | 0 | 0 | 0 | 0 | 0 | 0.27635 | 0.50946 | 0 | 0 |
| *MsWRKY10* | 0 | 0 | 4.114109 | 3.337173667 | 2.9568143 | 2.3447427 | 0 | 0 | 0.07416 | 0 |
| *MsWRKY11* | 31.0698 | 28.2361 | 10.48033533 | 22.02014233 | 15.776253 | 5.6481807 | 11.2543 | 5.41404 | 9.83053 | 8.50163 |
| *MsWRKY12* | 137.427 | 97.8867 | 2.850237667 | 9.405161 | 24.452793 | 3.201404 | 2.25338 | 3.23005 | 7.91327 | 20.7509 |
| *MsWRKY13* | 49.0605 | 6.70784 | 2.315011667 | 7.735048 | 25.709042 | 2.849151 | 11.888 | 3.43568 | 3.22462 | 5.66313 |
| *MsWRKY14* | 33.3384 | 5.80707 | 1.823928667 | 5.598291667 | 17.173431 | 1.7742067 | 9.55844 | 2.18184 | 2.39406 | 6.81716 |
| *MsWRKY15* | 56.1403 | 8.83778 | 2.925132 | 8.675463 | 32.068593 | 3.3417387 | 28.3951 | 7.17014 | 6.7315 | 15.3568 |
| *MsWRKY16* | 0 | 0 | 0 | 0 | 0 | 0 | 0 | 0 | 0 | 0 |
| *MsWRKY17* | 0 | 0 | 0 | 0 | 0 | 0 | 0.13041 | 0 | 0.29119 | 0 |
| *MsWRKY18* | 33.5794 | 7.43532 | 1.911103 | 3.720756333 | 0 | 18.06889 | 3.10274 | 1.83155 | 2.94131 | 5.51609 |
| *MsWRKY19* | 11.5137 | 9.51516 | 1.748492667 | 3.024759333 | 2.523078 | 7.060512 | 0.30003 | 0.19244 | 0.44301 | 1.00034 |
| *MsWRKY20* | 19.9956 | 1.08935 | 0.382441667 | 2.148622333 | 6.998028 | 0.6170223 | 0.76954 | 0.24072 | 0.36627 | 1.14578 |
| *MsWRKY21* | 3.1378 | 0.32701 | 0.166841 | 0 | 0 | 0 | 0 | 0 | 0 | 0 |
| *MsWRKY22* | 6.43936 | 0.15708 | 0 | 0 | 0 | 0 | 0.0947 | 0 | 0.432 | 0 |
| *MsWRKY23* | 0 | 0 | 0 | 0 | 0 | 0 | 0 | 0 | 0 | 0.07245 |
| *MsWRKY24* | 4.49025 | 0.32308 | 0 | 0 | 0 | 0 | 0.02779 | 0 | 0 | 0 |
| *MsWRKY25* | 72.6385 | 20.637 | 3.861316333 | 9.843237667 | 9.9085193 | 1.8228497 | 0.03594 | 0.13206 | 4.42283 | 11.6686 |
| *MsWRKY26* | 2.07147 | 0.60711 | 0.228777667 | 0.039712667 | 0.3399187 | 0.1133613 | 0.08448 | 0.1543 | 0.21924 | 0.98105 |
| *MsWRKY27* | 394.641 | 45.17 | 21.84749767 | 21.97765367 | 75.118176 | 8.7855313 | 2.5787 | 0.06377 | 1.65684 | 4.6992 |
| *MsWRKY28* | 1.05925 | 0.55004 | 0.456260667 | 0.510185333 | 0.1378283 | 0.4459037 | 2.85483 | 2.68675 | 1.23752 | 1.04567 |
| *MsWRKY29* | 3.95449 | 2.5712 | 3.645995333 | 4.614566667 | 3.009649 | 2.164184 | 5.31211 | 5.4124 | 4.59284 | 4.94549 |
| *MsWRKY30* | 51.7816 | 10.2828 | 6.669450333 | 10.641115 | 25.712649 | 2.558493 | 24.1851 | 7.25675 | 9.06198 | 11.7946 |
| *MsWRKY31* | 139.801 | 65.0491 | 98.86101033 | 76.730881 | 76.315903 | 35.258328 | 3.35047 | 2.22242 | 2.91377 | 4.5136 |
| *MsWRKY32* | 2.13157 | 0.055 | 12.86116133 | 11.028905 | 0.9941083 | 1.289541 | 0.8852 | 1.59115 | 3.47602 | 2.83811 |
| *MsWRKY33* | 5.20923 | 0 | 0.655507 | 4.545713333 | 4.505435 | 2.9315003 | 0 | 0 | 0 | 0 |
| *MsWRKY34* | 4.95068 | 0 | 1.433370667 | 0.667713 | 1.113348 | 2.2131073 | 0.64399 | 0 | 0 | 0.07242 |
| *MsWRKY35* | 5.91188 | 1.75702 | 0 | 0.244115333 | 0 | 0 | 0 | 0 | 0 | 0.05926 |
| *MsWRKY36* | 135.382 | 18.7972 | 40.588489 | 51.884243 | 106.33484 | 34.632675 | 131.937 | 47.2143 | 28.7618 | 22.1981 |
| *MsWRKY37* | 17.4964 | 3.42261 | 0.320881 | 0.909325667 | 2.2344647 | 0.5897413 | 2.85428 | 0.35997 | 0.20277 | 0.52511 |
| *MsWRKY38* | 0 | 0 | 0 | 0 | 0 | 0 | 0 | 0 | 0 | 0 |
| *MsWRKY39* | 7.58669 | 6.05947 | 6.56606 | 9.529369667 | 5.6762027 | 4.065712 | 5.0499 | 6.09803 | 5.7586 | 5.92917 |
| *MsWRKY40* | 19.7285 | 5.78131 | 6.817340333 | 15.12765233 | 20.186142 | 3.3600743 | 0.32693 | 3.69448 | 4.46835 | 3.84023 |
| *MsWRKY41* | 12.6252 | 2.57928 | 3.077837 | 9.504328333 | 14.661638 | 2.1191133 | 0.11875 | 0.06709 | 0.09472 | 0.51023 |
| *MsWRKY42* | 18.8718 | 1.37954 | 0.274733667 | 0 | 0 | 0 | 0.18229 | 0.04711 | 3.08347 | 4.54377 |
| *MsWRKY43* | 4.6797 | 14.4514 | 1.538162333 | 5.030419667 | 4.6835063 | 1.6862923 | 2.34958 | 0.67684 | 0.04368 | 1.72972 |
| *MsWRKY44* | 74.5854 | 29.1155 | 0.312655 | 2.702158333 | 6.8409617 | 0.2284457 | 1.02529 | 3.36188 | 7.88315 | 8.98743 |
| *MsWRKY45* | 5.69439 | 3.41019 | 9.444919 | 6.108887 | 8.7874593 | 5.6554983 | 0.02542 | 0 | 0.03495 | 0.43457 |
| *MsWRKY46* | 6.71914 | 10.6902 | 6.329216667 | 7.426236 | 4.9685053 | 7.003106 | 0.60398 | 0.38612 | 0.2486 | 0.31356 |
| *MsWRKY47* | 4.98818 | 0 | 3.006765 | 0.147675333 | 2.168121 | 0.377783 | 0.44021 | 0.79862 | 11.0559 | 4.02838 |
| *MsWRKY48* | 204.727 | 46.5365 | 18.01877133 | 22.93527433 | 46.762147 | 7.8878313 | 0.42194 | 0.49244 | 2.28041 | 7.71939 |
| *MsWRKY49* | 10.2838 | 8.66538 | 4.940139 | 7.143609 | 2.137452 | 1.9304947 | 1.91929 | 1.13316 | 1.6128 | 1.61502 |
| *MsWRKY50* | 12.579 | 4.62569 | 2.389093667 | 3.101317667 | 2.798798 | 0.6227273 | 10.9441 | 9.28348 | 3.78387 | 4.06457 |
| *MsWRKY51* | 3.03924 | 56.7797 | 3.859046333 | 13.07576733 | 4.2192117 | 4.0742637 | 0.34613 | 0.31126 | 0.09309 | 0.05796 |
| *MsWRKY52* | 9.81797 | 9.50379 | 20.017147 | 15.94824667 | 23.788134 | 10.644388 | 0.27691 | 0.03711 | 0.04681 | 0.36973 |
| *MsWRKY53* | 0.88383 | 0.18764 | 0.106653 | 0.041054 | 0.090767 | 0.0491003 | 0.81471 | 0.18226 | 0.00579 | 0.24643 |
| *MsWRKY54* | 0 | 0 | 2.234822333 | 3.019654333 | 5.584865 | 1.5171967 | 0.08819 | 0.27866 | 0.73819 | 0.2179 |
| *MsWRKY55* | 0 | 0 | 1.918912333 | 2.440069333 | 2.5757233 | 0.2429427 | 0.32121 | 0.1909 | 1.15384 | 0.75908 |
| *MsWRKY56* | 0 | 0 | 0.2302 | 0.736074333 | 0.1791803 | 0 | 0.02836 | 0.09557 | 0.30021 | 0.37441 |
| *MsWRKY57* | 0.09818 | 0.35731 | 1.365566 | 3.611604667 | 3.0527023 | 0.5684823 | 0.05546 | 0.36776 | 0.2313 | 0.21756 |
| *MsWRKY58* | 55.554 | 14.0363 | 12.30469933 | 28.76381633 | 32.79083 | 10.203655 | 48.1766 | 9.96478 | 3.49261 | 4.81026 |
| *MsWRKY59* | 0.07835 | 0.57324 | 0 | 0.041984667 | 0 | 0.1184853 | 0.13405 | 0.18974 | 0.01769 | 0.01777 |
| *MsWRKY60* | 17.2453 | 12.8209 | 19.34405333 | 28.77214633 | 12.691363 | 7.8010107 | 5.53124 | 12.1702 | 12.7817 | 20.1741 |
| *MsWRKY61* | 0 | 0.18123 | 0.044035333 | 0 | 0 | 0.0639947 | 0 | 0.03881 | 0.12461 | 0.04374 |
| *MsWRKY62* | 0 | 0 | 0 | 0 | 0 | 0 | 0 | 0 | 0 | 0 |
| *MsWRKY63* | 0 | 0 | 0 | 0 | 0 | 0 | 0 | 0 | 0 | 0 |
| *MsWRKY64* | 0 | 371.881 | 0 | 0 | 0 | 0 | 0 | 0 | 0 | 0 |
| *MsWRKY65* | 0 | 0 | 0 | 0 | 0 | 0 | 0 | 0.01242 | 0 | 0 |
| *MsWRKY66* | 4.6749 | 58.0254 | 0 | 0 | 0 | 0 | 0.03523 | 0.02022 | 0.12964 | 0.39456 |
| *MsWRKY67* | 17.6347 | 11.1309 | 16.392311 | 18.52266367 | 22.575457 | 12.136859 | 10.8583 | 14.2469 | 9.96502 | 15.9129 |
| *MsWRKY68* | 9.55007 | 8.93038 | 9.128247333 | 8.473603 | 9.582375 | 12.610795 | 8.65291 | 7.69165 | 6.02895 | 5.41045 |
| *MsWRKY69* | 0 | 0 | 0 | 0 | 0 | 0 | 0 | 0 | 0 | 0 |
| *MsWRKY70* | 1.44393 | 0.44001 | 2.221807667 | 0 | 0.1262027 | 0.472197 | 0 | 0.132 | 0.6024 | 7.28075 |
| *MsWRKY71* | 117.755 | 26.0753 | 5.234908333 | 8.170811667 | 7.7276893 | 1.932703 | 3.4478 | 1.28755 | 0.87143 | 4.24768 |
| *MsWRKY72* | 104.567 | 54.0984 | 170.912196 | 155.7193347 | 195.25882 | 101.08743 | 0.50674 | 0.2458 | 0.78769 | 2.86431 |
| *MsWRKY73* | 49.3916 | 24.9437 | 29.59714067 | 19.92332067 | 35.141568 | 14.556704 | 0.03657 | 0.06617 | 0 | 0.14383 |
| *MsWRKY74* | 19.7902 | 8.14245 | 8.668678667 | 15.78966233 | 17.066003 | 19.324248 | 5.35336 | 5.12879 | 2.80547 | 4.47665 |
| *MsWRKY75* | 3.82433 | 4.2488 | 0.479516 | 1.411673333 | 0 | 0.0938233 | 0 | 0 | 0 | 0 |
| *MsWRKY76* | 0.05767 | 0 | 0 | 0 | 0 | 0 | 0.04251 | 0 | 0.00222 | 0 |
| *MsWRKY77* | 0 | 0 | 0 | 0 | 0 | 0.4196113 | 0.01866 | 0.03187 | 0.07021 | 0.18282 |
| *MsWRKY78* | 0.29957 | 0 | 1.168760667 | 2.556591 | 1.5459047 | 10.136531 | 0.63479 | 0.19521 | 0.61099 | 8.12745 |
| *MsWRKY79* | 35.9992 | 5.40838 | 15.042556 | 37.88587433 | 20.400656 | 5.0644593 | 14.3785 | 29.25 | 34.4587 | 39.9658 |
| *MsWRKY80* | 17.5575 | 13.8595 | 9.343129333 | 20.414473 | 10.200286 | 6.6203893 | 5.69513 | 2.59494 | 1.02346 | 2.35067 |
| *MsWRKY81* | 4.09888 | 1.31819 | 0.084184333 | 0 | 1.3396493 | 0.096055 | 0.27749 | 0.08476 | 0 | 0.11216 |
| *MsWRKY82* | 1.86639 | 0 | 1.951524667 | 9.232268 | 88.319179 | 4.7082447 | 0.15899 | 0.10631 | 0.47246 | 0.35507 |
| *MsWRKY83* | 0.2471 | 0.45693 | 29.47293533 | 44.94701733 | 0.7653457 | 0 | 0 | 0.02383 | 0 | 0.07962 |
| *MsWRKY84* | 21.2646 | 3.45436 | 3.25808 | 2.088198333 | 1.2748053 | 0.228209 | 9.24567 | 13.7888 | 8.15364 | 6.18506 |
| *MsWRKY85* | 0 | 0 | 0 | 0 | 0 | 0 | 0.05629 | 0.01158 | 0 | 0 |
| *MsWRKY86* | 71.147 | 24.2296 | 29.29410233 | 38.89243433 | 29.167052 | 23.85176 | 44.3641 | 26.3533 | 25.5388 | 30.4494 |
| *MsWRKY87* | 25.3092 | 1.86252 | 0.049574333 | 0.286300667 | 0.4868147 | 0.165128 | 0.53225 | 0.16827 | 0.08263 | 0.59583 |
| *MsWRKY88* | 1.86409 | 0.27899 | 1.408423333 | 1.732688333 | 1.1880333 | 1.947184 | 3.33146 | 0.31496 | 0.1943 | 0.70692 |
| *MsWRKY89* | 1.15168 | 0 | 0.421022333 | 0.75733 | 3.1801577 | 0.607423 | 1.00269 | 2.07412 | 0.85853 | 4.21395 |
| *MsWRKY90* | 28.2099 | 18.6388 | 21.076315 | 24.17532733 | 18.725875 | 19.743245 | 24.6652 | 13.9619 | 7.12302 | 6.81549 |
| *MsWRKY91* | 5.89242 | 0.6188 | 0.850559333 | 0.511119667 | 1.1652913 | 0.3339013 | 0.62807 | 0.29311 | 0.35367 | 2.23598 |
