# Supplementary material for: Genomic identification and expression profiling of WRKY genes in alfalfa (Medicago sativa) elucidate their responsiveness to seed vigor
Source: BMC Plant Biol. 2023 Nov 16;23:568. doi: 10.1186/s12870-023-04597-x (PMC10652462; doi:10.1186/s12870-023-04597-x)
Supplement: Supplementary file 9 — Additional file 9: Table S9. Sequences of primers used in RT-qPCR. [file 12870_2023_4597_MOESM9_ESM.docx]

**Table S9: Sequences of primers used in RT-qPCR**

| **Gene Name** | **Forward primer sequence (5′ → 3′)** | **Reverse primer sequence (5′ → 3′)** |
| --- | --- | --- |
| *MsWRKY06* | CAAAAGATGGCAGATGCTGAGGAT | CATGACACCAGTATGACGAGGTCG |
| *MsWRKY17* | CAACAGTTTGGCTATGGAA | TTTAGACCTTGTCGCTTAT |
| *MsWRKY35* | ACTTGGGTTCACATAACAA | TTTCAGAATAAGGGAGGTT |
| *MsWRKY41* | AAATCGGAACAGGAGACAA | CGGTTTGGAGCCATACTTC |
| *MsWRKY52* | GGCTAATGACGACGGCTAC | AGGATCGCGTTCAACTTTT |
| *MsWRKY54* | GGAGAAAGTATGGGCAGAA | TGTGGATGCCAATGTATGT |
| *MsWRKY83* | CGGGCTTTATTCATTCTTG | TTCCCATCTTGGAGTTCAG |
| *MsWRKY89* | AAAGAAGGGCTATGGAAGG | TGTAGAGGGTGACAAGAAG |
| *Msactin* | GCAGCAGAACCAACAATCT | GACACCCATCACTCATCAA |
